# Transcriptome/Degradome-Wide Identification of *R. glutinosa* miRNAs and Their Targets: The Role of miRNA Activity in the Replanting Disease

**DOI:** 10.1371/journal.pone.0068531

**Published:** 2013-07-05

**Authors:** Ming Jie Li, Yan Hui Yang, Xin Jian Chen, Feng Qing Wang, Wen Xiong Lin, Yan Jie Yi, Lei Zeng, Shuo Ye Yang, Zhong Yi Zhang

**Affiliations:** 1 College of Life Sciences, Henan Agricultural University, Zhengzhou, China; 2 College of Bioengineering, Henan University of Technology, Zhengzhou, China; 3 College of Crop Sciences, Fujian Agriculture and Forestry University, Fuzhou, China; Universidad de Salamanca, Spain

## Abstract

*Rehmannia glutinosa*, a traditional Chinese medicine herb, is unable to grow normally in a soil where the same species has recently been cultivated. The biological basis of this so called “replanting disease” is unknown, but it may involve the action of microRNAs (miRNAs), which are known to be important regulators of plant growth and development. High throughput Solexa/Illumina sequencing was used to generate a transcript library of the *R. glutinosa* transcriptome and degradome in order to identify possible miRNAs and their targets implicated in the replanting disease. A total of 87,665 unigenes and 589 miRNA families (17 of which have not been identified in plants to date) was identified from the libraries made from a first year (FP) and a second year (SP) crop. A comparison between the FP and SP miRNAs showed that the abundance of eight of the novel and 295 of the known miRNA families differed between the FP and SP plants. Sequencing of the degradome sampled from FP and SP plants led to the identification of 165 transcript targets of 85 of the differentially abundant miRNA families. The interaction of some of these miRNAs with their target(s) is likely to form an important part of the molecular basis of the replanting disease of *R. glutinosa*.

## Introduction

The herbaceous species *Rehmannia glutinosa* L (*Scrophulariaceae*) is of some economic importance because extracts from its tuberous roots are medicinally active [1]. Although the plant is perennial, its productivity declines significantly after the first year [2], [3], a syndrome described as `replanting disease”, and thought to be due to the release of autotoxins into the soil either in the form of exudate from the roots or of leachate into the soil from above ground residue left after harvest [4], [5]. The phenomenon is referred to more generally as “allelopathic autotoxicity” [6].

Gene expression can be regulated at multiple levels. A recently discovered mode of post-transcriptional regulation involves microRNAs, small RNA molecules (averaging typically 21–24 nt in length) able to regulate the expression of specific genes by targeting their mRNAs for degradation [7]–[10]. The specificity of a given miRNA’s target is achieved via its complementarity with the sequence of its target mRNA. An increasing weight of evidence has shown that miRNAs are intimately involved in many adaptive responses to both abiotic and biotic stress [9], [11]. Given that allelopathic autotoxicity represents a form of stress, it is possible that miRNAs are involved in the replanting disease of *R. glutinosa.*

High throughput sequencing platforms have created numerous opportunities for the characterization of genomes and transcriptomes, especially in non-model organisms for which the full genome sequence has yet to be acquired [12]–[17]. The genomic resources available for *R. glutinosa* are very limited [18]. Here we describe the application of the Solexa/Illumina platform to obtain a global view of the *R. glutinosa* transcriptome, including the population of small RNAs (sRNAs) and the degradation products of mRNA (the “degradome”) present. In particular, the focus was to identify novel miRNAs in *R. glutinosa* by transcriptome, to detect those miRNAs which were differentially abundant in plants suffering from the replanting stress by sRNA sequencing and qRT-PCR, and to identify their target mRNAs by degradome analysis. The purpose was to reveal the possible functions of miRNA and their targets in forming of replanted disease in *R. glutinosa*.

## Results

### Transcriptome Sequencing and de novo Assembly Analysis

The root and leaf libraries each comprised ∼41 million 75bp paired end raw sequence reads, which were deposited at NCBI under the accession number SRX269425 and SRX269426, and this number was reduced to, respectively, ∼38 and 39 million Q20 (base quality more than 20) standard reads by applying a stringent quality check (Table A in File S1). Use of the assembly software SOAPdenovo (http://soap.genomics.org.cn/), developed specifically to process short reads, yielded 99,708 unigenes (mean length 348 bp) from the root and 94,544 (mean length 368 bp) from the leaf transcript library. Root and leaf libraries were further assembled and incorporated in an entire *R. glutinosa* transcriptome. The removal of partially overlapping sequences finally produced a set of 87,665 unigenes (mean length 554 bp), representing in all 41.82 Mbp of sequence (Table 1, Fig. A in File S2). The entire transcriptome was used to analyze *R. glutinosa* sRNA and degradome libraries.

**Table 1:** Length distribution of assembled Contig, Scaffold, Unigene and All-Unigene in *R. glutinosa*.

Nucleotides length (bp)	Contig		Scaffold		Unigene		All-Unigene
	R	L	R	L	R	L	
75–100	488,952	349,090	0	0	0	0	0
101–500	180,322	169,244	153,722	140,812	84,599	78,290	64,810
501–1000	9,405	9,959	11,214	11,784	11,096	11,696	15,293
1001–2000	2,118	2,157	3,460	3,837	3,449	3,815	6,041
2001–3000	185	255	481	645	481	639	1,205
>3000	31	45	88	104	83	104	316
Total	681,013	530,950	168,965	157,182	99,708	94,544	87,665
Minimum length (bp)	75	75	100	100	200	200	300
Maximum length (bp)	4,475	4,127	4,475	4,127	4,475	4,127	6,238
N50 (bp)	97	122	299	330	384	420	554
Average Lenth (bp)	127	137	256	271	348	368	477
Total Nucleotides length (bp)	86,286,882	72,965,314	43,244,769	42,519,022	34,719,506	34,806,569	41,829,880

### Sequencing and Analysis of sRNAs

**Figure 1:**
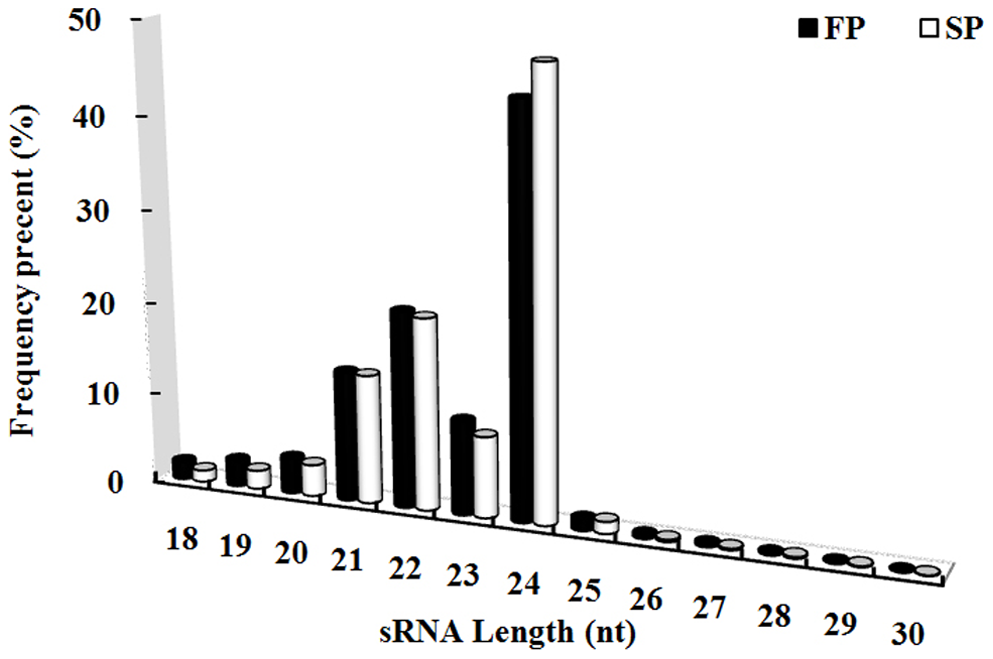
Length distribution of the FP and SP libraries.

The two sRNA libraries derived from FP and SP material produced, respectively, 17,723,851 and 18,123,606 raw reads. After removal of low-quality and corrupted adaptor sequences (reads<18 nt), a total of 14,658,978 and 15,649,864 clean reads corresponding to 6,798,635 and 7,917,831 unique reads remained for FP and SP libraries (Table B in File S1). Of the combined reads, 28.40% was FP-specific, 32.94% was SP-specific and 38.66% was present in both libraries (Fig. B in File S2). The length of most of these small RNAs lay in the range 21–24 nt, with 24 nt molecules predominating (Fig. 1).

### Identification of known miRNAs

The sRNA sequences were used to interrogate the relevant GenBank database (http://www.ncbi.nih.gov/Genbank/) using Rfam 10.1 software (http://rfam.sanger.ac.uk/), resulting in the annotation of 0.92% of the FP and 0.62% of the SP unique non-protein-coding sRNAs (rRNA, snRNA, snoRNA and tRNA). The remained sRNA sequences were then compared to those deposited in miRBase 19.0 (www.mirbase.org/), which on the basis of <3 mismatched nucleotides produced 546,181 (unique 1,913) hits in the FP library, and 314,312 (unique 1,434) in the SP library. These hits accounted for, respectively, 3.73% and 2.00% of the FP and SP sRNA sequence (Table 2). A total of 768 miRNAs, belonging to 572 already documented miRNA families, was identified across the two sRNA libraries. Based on their conservation, these known miRNAs were classified into three (conserved, non-conserved and *Rehmannia*-specific) groups (Table C in File S1) [19]. The group I was 27 miRNA families (126 members) for that have already been identified in both monocotyledonous and dicotyledonous species. Some of the miRNAs, notably miR156/157, miR159, miR172, miR166 and miR167, were highly abundant in both libraries, whereas others were present in much lower abundance. The group II, which exists only in no more than ten plant species (miRBase 19.0, http://www.mirbase.org/), was also detected 539 miRNA families (636 members) from *R. glutinosa*. Most of these miRNAs were of low to moderate abundance. While the copy number of miR2870, miR2937, miR4248 and miR918 was notably higher in the SP than in the FP library, that of miR1860, miR2123, miR2635 and miR948 was higher in the FP library. Finally, the group III of six *Rehmannia*-specific miRNAs was identified, all of which were present in low abundance in both libraries.

**Table 2:** Abundance of different sRNAs from SP and FP libraries.

Category	Unique sRNA number (percentage)		Total sRNA number (percentage)	
	FP	SP	FP	SP
Non-protein-coding RNAs	62,799(0.92)	48,749(0.62)	625,048(4.26)	483,397(3.08)
Known miRNAs	1,913(0.03)	1,434(0.02)	546,181(3.73)	314,312(2.00)
Mapped to transcriptome	3,956(0.06)	3,782(0.05)	9,946(0.07)	9,239(0.06)
Other sRNAs	6,729,967(98.99)	7,863,866(99.32)	13,477,812(91.94)	14,842,916(94.85)
Total	6,798,635(100.00)	7,917,831(100.00)	14,658,987(100.00)	15,649,864(100.00)

### Identification of Novel miRNAs

**Figure 2:**
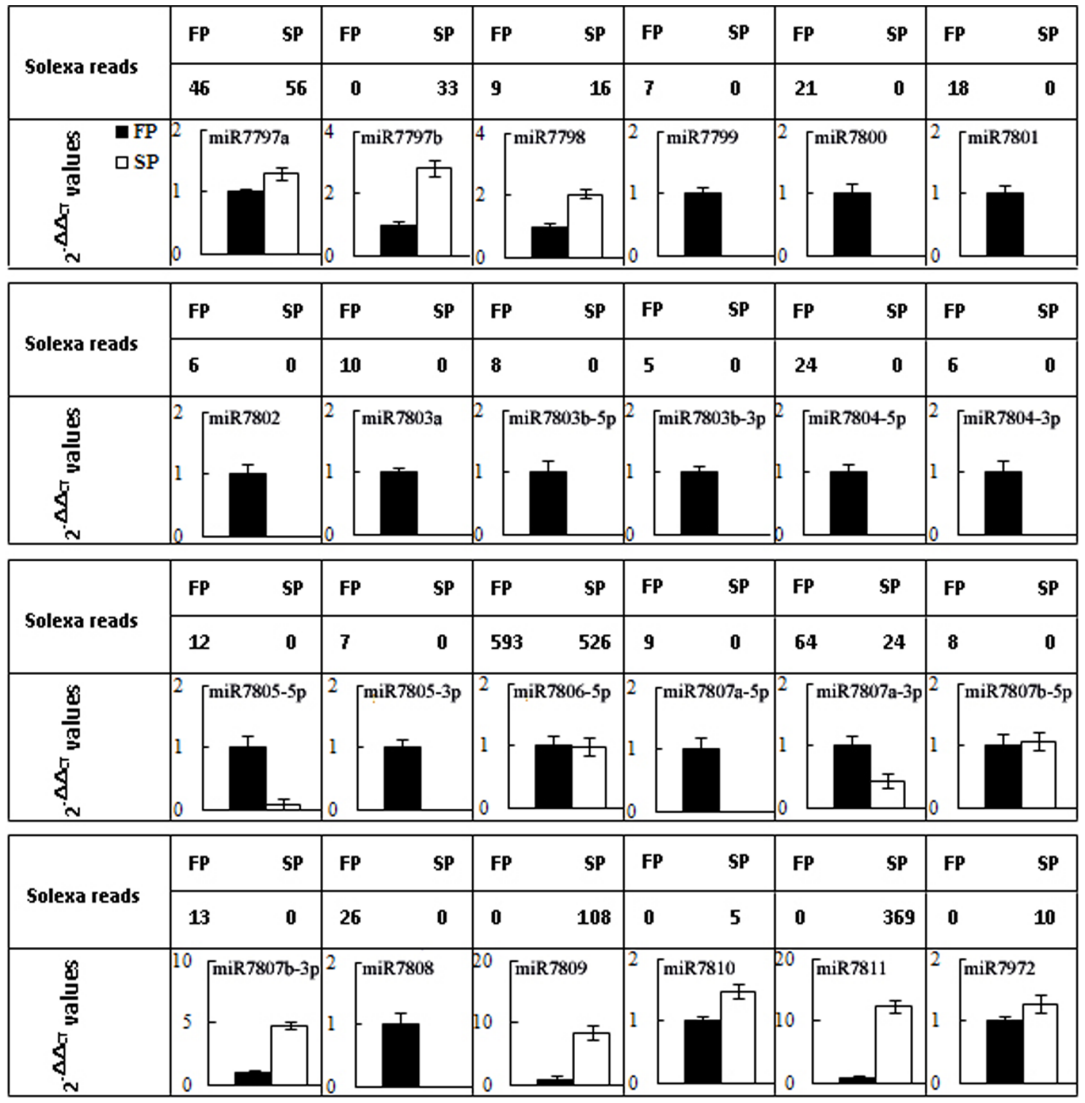
Verification of novel miRNAs by qRT-PCR in FP and SP *R. glutinosa*.

After removal of the annotated sRNA and known miRNAs, 13,487,758 (6,733,923 unique sequences) FP and 14,852,155 (7,867,648 unique sequences) SP sequences were remained in the libraries (Table 2). 9,946 (3,956 unique sequences) FP and 9,239 (3,782 unique sequences) SP sequences of these sRNAs were mapped onto the *R. glutinosa* 87,665 unigenes with a view to identifying their targets on the basis of the presence of the diagnostic hairpin structures flanking sequences [20], [21]. 41 unique sequences of 35 pre-miRNAs across the two libraries could be considered as candidate novel miRNAs (Table 3), which were not registered in miRBase 19.0. And six of them included miRNA* sequences in the libraries. The abundance of 34 of the presumed miRNAs was low (each being recovered <100 times). The presence of 24 of the 41 candidate miRNAs was verified using qRT-PCR analysis (Fig. 2, Fig. C in File S2). We took these 24 miRNAs (belonging to 17 miRNA families) genuine novel or specific miRNAs in *R. glutinosa* (File S3).

**Table 3:** Candidates of novel miRNAs from *R. glutinosa*.

miRNA ID	Precursor gene ID	Precursor length (nt)	Reads		Sequences	Energy(kcal mol^−1^)		qRT-PCRidentification
			FP	SP				
Z2	Unigene12916_All	144	6	0	ACAUUAGGACAAAUUAUCACG		−51.6	No
rgl-miR7797a	Unigene151_All	141	46	56	AAGACGGAAUCAAACCUCAA		−48.2	Yes
rgl-miR7797b	Unigene45997_All	126	0	33	UUUGAUUUCGUCUUACAUUUUUC		−19.37	Yes
Z5	Unigene20830_All	84	13	0	AGAGGAGUGUACCAUCACCGCC		−44.3	No
rgl-miR7798	Unigene21450_All	229	9	16	AGGGAGUGUUUGCAAAAACU		−67.1	Yes
Z8	Unigene26960_All	200	208	106	UUGUCAGGCUUGUUAUUCUCC		−68.6	No
Z9	Unigene30417_All	272	27	27	CGAGUAGCGCUAGAGGUCCAACA		−66.86	No
Z10	Unigene30676_All	107	58	0	AUCCAUGGGGAGAGGAAGCACA		−43.52	No
Z11	Unigene32348_All	72	81	0	CUUUUAUAAAGCUGUCGGGACA		−27.4	No
rgl-miR7799	Unigene34159_All	125	7	0	AGUGGAAUAGGAGAUCUCAA		−44.63	Yes
Z14	Unigene40078_All	91	17	14	AGGGGAAGGAUUUCAAAUGAC		−38.6	No
Z16	Unigene40998_All	92	6	0	AGAGGGAGUACUAUUGAAGGA		−36	No
rgl-miR7800	Unigene51345_All	95	21	0	UAUUUUUGUGUCGUUAUGGUC		−35.2	Yes
rgl-miR7801	Unigene51544_All	128	18	0	UACGAGAUGAAACACAGUUUG		−38.2	Yes
rgl-miR7802	Unigene51775_All	106	6	0	AGGGAGUGUUUGCAAUCACUAAA		−28.17	Yes
rgl-miR7803a	Unigene51839_All	152	10	0	UACGGAUAAUUGACACGUGUAUA		−62.4	Yes
rgl-miR7803b-5p	Unigene84940_All	117	8	0	GGAUGAUUGCCACGUGUAUA		−56.8	Yes
rgl-miR7803b-3p			5	0	UACACGUGUCAAUCAUCUAU			Yes
Z24	Unigene52051_All	113	135	0	GGCGAACUGCUCGAGCUGCU		−35.9	No
rgl-miR7804-5p	Unigene55578_All	160	24	0	AGGGGUGUUCAUCGAAUCGAAUU		−42.7	Yes
rgl-miR7804-3p			6	0	UUUAAUCGAAUGAACAUUUUAAA			No
Z27	Unigene57696_All	97	18	0	ACCGUUGAUGGUAUCAAAAUC		−47.4	No
rgl-miR7805-5p	Unigene64556_All	69	12	0	AAAUUUGGUGUAGUGAAUAGU		−38.7	Yes
rgl-miR7805-3p			7	0	UAUUCAUUUACACCAAAUUUGG			No
Z33	Unigene73437_All	184	109	50	GUAGCAUCAUCAAGAUUCACA		−68.3	No
rgl-miR7806	Unigene74190_All	126	593	526	UAGAAGAUGUCCACAUGAGCA		−38.27	Yes
rgl-miR7807a-5p	Unigene82131_All	138	9	0	AACUAUAUGAAAAUCUCAAUU		−41.4	Yes
rgl-miR7807a-3p			64	24	UUGGGAUUUGCAUACAGUUAC			Yes
rgl-miR7807b-5p	Unigene40448_All	144	8	0	UAACUAUAUGAAAAUCUCAAUU		−59.2	Yes
rgl-miR7807b-5p			13	0	UUGAGAUUUUCAUAUAGUUACU			Yes
Z38-5p	Unigene87020_All	117	162	0	ACUCGCAUCUACAAGAACAUA		−49.02	No
Z38-3p			69	0	UGUUCUUGUAAAUGGGUAGUUA			No
rgl-miR7808	Unigene891_All	315	26	0	AAGGAUGCUCGAUUCAGAAGAA		−76.69	Yes
Z40	Unigene891_All	248	9	0	UUCUUCUGAAUCGAGCAUCCUU		−42.14	No
C03	Unigene17031_All	168	0	8	CAACUCUGGAUAUUGAUUCCUCA		−30.45	No
rgl-miR7809	Unigene18424_All	115	0	108	UCCCAUUGCAUCAGCGGACACA		−31.82	Yes
C05	Unigene21450_All	229	0	10	AGGGAGUGUUUGCAAAAACU		−67.1	No
rgl-miR7810	Unigene26764_All	91	0	5	AGAGGAAGAGUUUUCUGGCUC		−26	Yes
rgl-miR7811	Unigene41058_All	86	0	369	UGAAUGGAGAUACGGAAUGAAGC		−22	Yes
rgl-miR7972	Unigene30260_All	207	0	10	UUGUCAGGCUUGUUAUUCUCC		−53.71	Yes
C21	Unigene49775_All	90	0	9	AGAGGUCUGAGGUUCGAUUUUCA		−22	No

### The Differential Abundance of miRNAs in FP and SP Plants

**Figure 3:**
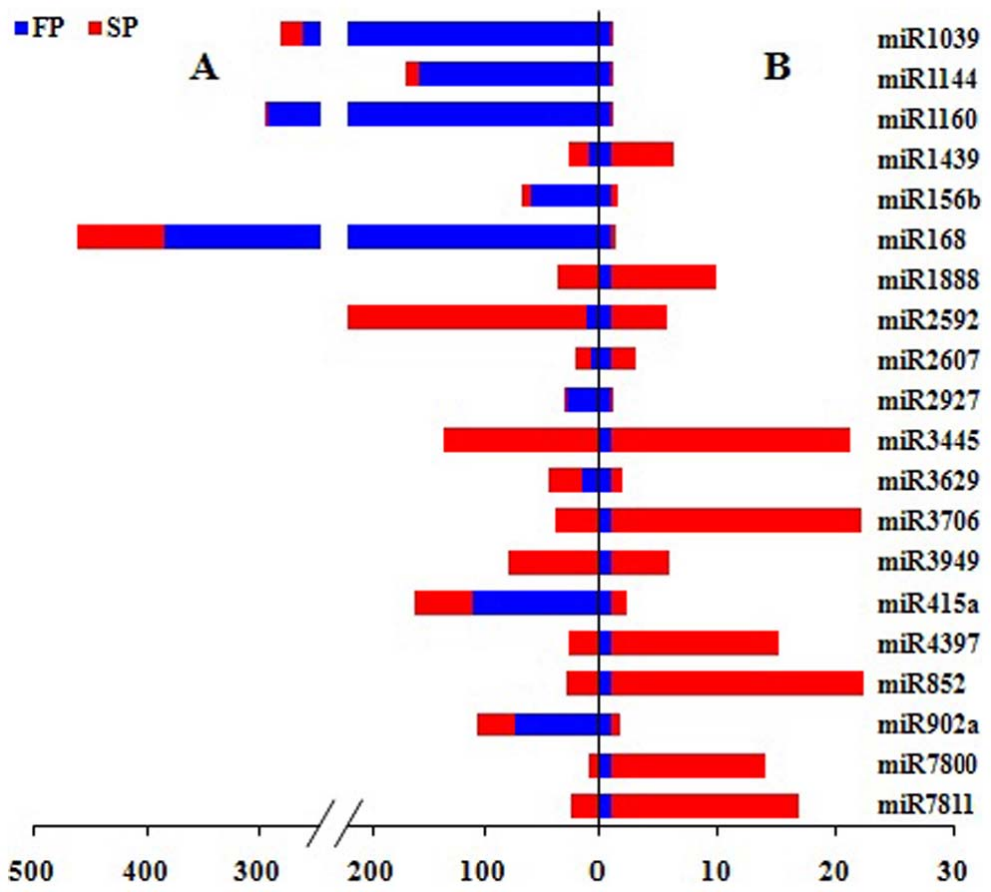
Comparison of partial differently expressed miRNAs in FP and SP R. *glutinosa* using high-throughput sequencing and qRT-PCR. A, the relative expression levels (normalized values) measured by high-throughput sequencing; B, the relative expression levels (2^−△△CT^) tested by qRT-PCR. Red, expressing levels in SP. Blue, expressing levels in FP.

A comparison of miRNA abundance in the FP and SP libraries revealed statistically different levels for 303 (eight *Rehmannia*-specific, 295 known) of the 589 families (p-value <0.01) (Table D in File S1). In all, 124 of these were more abundant in FP than in SP plants and the remainder vice versa. 54 and 62 miRNA families were specifically expressed and closed in SP library, respectively (Table 4). The result showed that miRNA expression levels could be altered in replanted *R. glutinosa*. A randomly chosen subset of 20 of the differentially abundant miRNAs present in moderate abundance was subjected to validation by qRT-PCR. The estimated abundance from the sequencing outcome and from the qRT-PCR analysis was consistent for 18 of the 20 (the exceptions were miR3269 and miR415a) (Fig. 3).

**Table 4:** Expressed difference of miRNA families in FP and SP *R. glutinosa*.

Types of expression	The number of expressed miRNA families		
	Known	Novel	Total
Total miRNA families	295	8	303
SP up-regulated	120	4	124
SP down-regulated	175	4	179
Only SP-expressed	51	3	54
SP-closed	58	4	62

### Degradome Sequencing Analysis

A total of ∼14.6 million (FP) and ∼21.1 million (SP) raw reads of 3′ cleavage fragments was obtained. These were reduced after manual editing to, respectively, ∼12.5 and 20.0 million clean reads (Table E in File S1), which were then filtered to exclude any structural RNAs (rRNA, tRNA, snRNA and snoRNA). This step removed only a small proportion (2,873 and 6,756, respectively) of the reads. The remaining reads were then aligned with the *R. glutinosa* transcriptome. In total, 367,290 (37.39%) and 769,336 (38.36%) unique reads from FP and SP degradome libraries, respectively, could be associated with the transcriptome (Table 5). The CleaveLand pipeline [22], [23] was used to identify degraded targets for each of the miRNA families which differed in abundance between FP and SP. The abundance of each sequence was plotted for each unigene target, and the degradation products were grouped into three categories (I–III) according to their relative abundance (File S4). In all, 165 target mRNAs were identified, involving 85 (including 33 up- and 52 down-regulated in the SP sRNA library) miRNA families (Table F in File S1). Among the miRNA families, 50 targeted a single transcript, and the highest number of targets cleaved by a single miRNA was nine (miR157). One mRNA (unigene35337_All), which putatively encodes a squamosa promoter binding protein, was possibly targeted by two miRNAs (miR156 and miR157). Of the 56 targets in the FP degradome library, 35 fell into category I and 12 each into categories II and III; for the SP libraries, 76 of the 137 targets fell into category I, 31 into category II and 30 into category III (Table 6). Analysis of the targets showed that the cleaved targets were differentially present between the two degradome libraries. These observations suggest that replanting promotes miRNA-driven cleavage in *R. glutinosa*.

**Table 5:** Abundance (percentage) of different RNAs from FP and SP *glutinosa* degradomes.

Category	Unique reads (Percentage)		Total reads (Percentage)	
	FP	SP	FP	SP
Total	982,398 (100.00)	1,984,865 (100.00)	12,497,249 (100.00)	19,992,843 (100.00)
rRNA	1,829 (0.19)	4,076 (0.21)	83,977 (0.67	142,510 (0.71)
tRNA	263 (0.03)	752 (0.04)	2,674 (0.02)	7,266 (0.04)
snRNA	386 (0.04)	1,064 (0.05)	4,978 (0.04)	9,793 (0.05)
snoRNA	395 (0.04)	864 (0.04)	4,007 (0.03)	7,570 (0.04)
Mapped to transcriptome	367,290 (37.39)	769,336 (38.76)	4,738,853 (37.92)	8,415,733 (42.09)
Other sRNAs	612,235 (62.32)	1,208,773 (60.90)	7,662,760 (61.32)	11,409,971 (57.07)

**Table 6:** Difference of cleaved targets between in FP and SP *R. glutinosa*.

Category of target	Number of targets		
	FP	SP	Unique total
I	35	76	91
II	12	31	38
III	12	30	36
Total	59	137	165

### Functional Analysis of miRNA Targets

**Figure 4:**
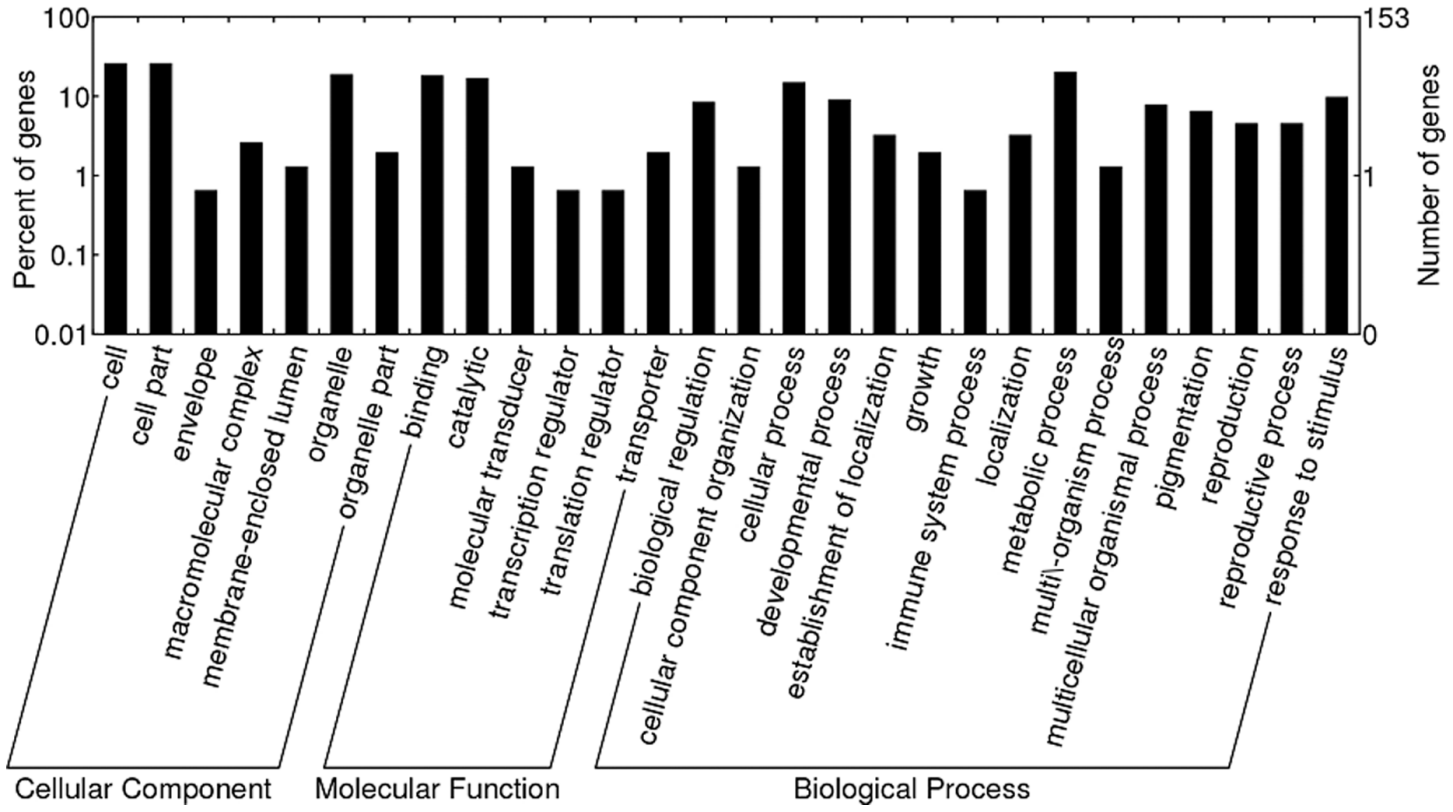
GO analysis of identified targets from the *R. glutinosa* degradome libraries.

A BlastX search of the Nr (non-redundant protein sequences) database showed that these miRNA targets shared homology with other plant proteins (Table F in File S1). The targets of the miRNA families which were more abundant in SP included genes encoding a WD-40 repeat family protein, a polyphenol oxidase, CBL-interacting protein kinase 18, a potassium ion transmembrane transporter 7, a histidine kinase 3B, MYB transcription factor 127 and an aspartic protease. These genes are all involved in plant growth and development. On the other hand, the targets of the miRNA families which were more abundant in FP included genes encoding a heat shock protein, a squamosa promoter-binding protein, a class III HD-Zip protein, AGO1-1 and RNA helicase, genes which are all involved in the stress response. When gene ontology categories were assigned to these targets (Fig. 4), six molecular function categories predominated, with the two most highly represented being “binding” and “catalytic”. Fifteen biological processes were identified, with the two most frequent being “cellular process” and “metabolic process”, followed by “development process” and “response to stimulus”. Finally there were seven major cellular component classes, with the two most abundant being “cell” and “cell part”. This analysis suggested that the miRNA targets were concentrated in biological regulation, response to stimuli, development and metabolic processes.

### Transcription Profiling of Selected miRNAs

**Figure 5:**
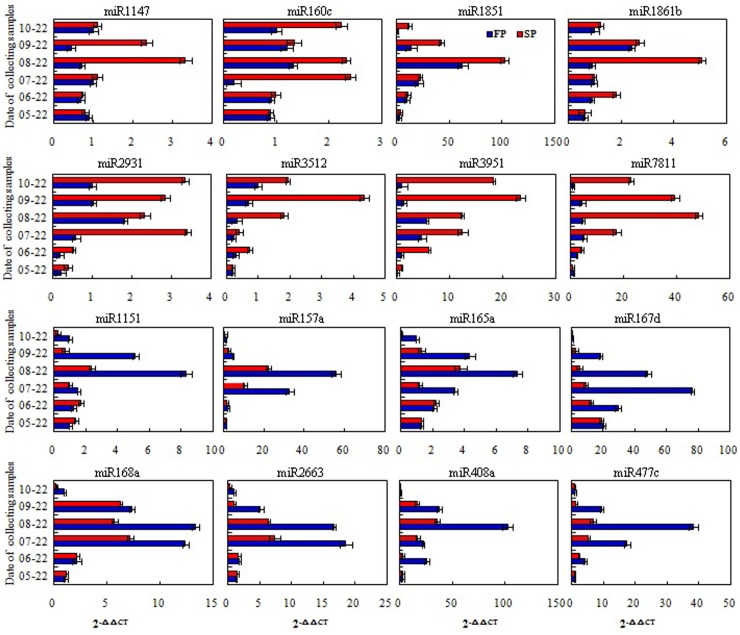
16 miRNA different expression patterns in FP and SP roots during developmental processes using qRT-PCR analysis. Red, expressing levels in SP. Blue, expressing levels in FP.

The temporal pattern of transcription of 16 selected miRNAs (eight of which were more abundant in FP and eight more abundant in SP) was obtained using qRT-PCR (Fig. 5). For each of the eight miRNAs selected as being more abundant in the SP root according to the sequencing data, transcript abundance was uniformly higher in the SP than in the FP root. The abundance of miR2931, miR3951 and miR7811 was particularly high throughout the four month sampling period, while that of miR1851, miR1147, miR160c, miR1861b and miR3512 appeared to be strongly induced only at certain measured times. Similarly, for each of the eight miRNAs selected as being more abundant in the FP root according to the sequencing data, transcript abundance was uniformly lower in the SP than in the FP roots. miR157a, miR167d, miR408a and miR477c were almost undetectable for a three month period, while miR1115, miR165a, miR168a and miR2663 were detectable at some, but not all of the sampling times.

## Discussion

### Transcriptome-wide Novel miRNAs Identification

Understanding the function of a given miRNA requires the recognition of its target mRNA(s). Little is yet known regarding the spectrum of miRNAs present in *R. glutinosa* [18], [24], particularly because only 1,500 EST sequences have as yet been deposited in GenBank. Here we have added greatly to this number by applying a high throughput sequencing platform, which has also been useful in characterizing the transcrptome in both the root and leaf. The number of miRNA sequences present in *R. glutinosa* has as a result been greatly expanded over what had been established prior to this study, and now numbers almost 800, of which the great majority (97%) have been identified previously in other plant species. Clearly, high throughput sequencing provides a highly effective means of acquiring miRNA sequence, especially in a species whose genome has yet to be properly characterized [25]–[28].

### Differential Abundance of miRNAs in FP and SP Plants

The Solexa/Illumina platform is able to provide an estimate of the abundance of individual miRNAs, based on the number of times that a specific sequence is recovered among the millions of sequence reads generated [29], [30]. This feature made it possible to recognize differences in the abundance of individual miRNAs in FP and SP plants. In the event, 303 miRNA families behaved differentially, with 124 families more abundant and 179 less abundant in SP than in FP. A number of these miRNAs may be involved in the regulation of the stress response [9], [31]. Of particular interest are the 54 families (51 of which also occur in other plants, and three which are specific to *R. glutinosa*) which were only present in SP. Some of these may well therefore be implicated in the replanting disease.

### The Identification of mRNA Targets by Analysis of the Degradome

Degradome sequencing was focused on the 165 targets identified for the 85 miRNA families present in differential abundance in the replanted *R. glutinosa*. Although there were 303 differentially abundant miRNAs in total, the other 218 miRNA families were not associated with any identifiable cleavage target. Possible reasons for this failure are first that the target was present at too low an abundance to have been recovered [31], [32], and second that at least some of these miRNAs are incapable of cleavage, acting instead by translational repression [33], as has been suggested elsewhere [31]. The predicted functions of the 165 targets were associated with growth, development and the stress response, along with certain other processes. Typical symptoms of the replanting disease of *R. glutinosa* include weak growth, the development of fibrous in preference to tuberous roots, the abnormal expansion of the tubers and precocious flowering and premature, etc [1], [6], [34]. The interesting question is whether any of these phenotypes could be determined by the action of any or some of the 85 differentially abundant miRNAs.

**Figure 6:**
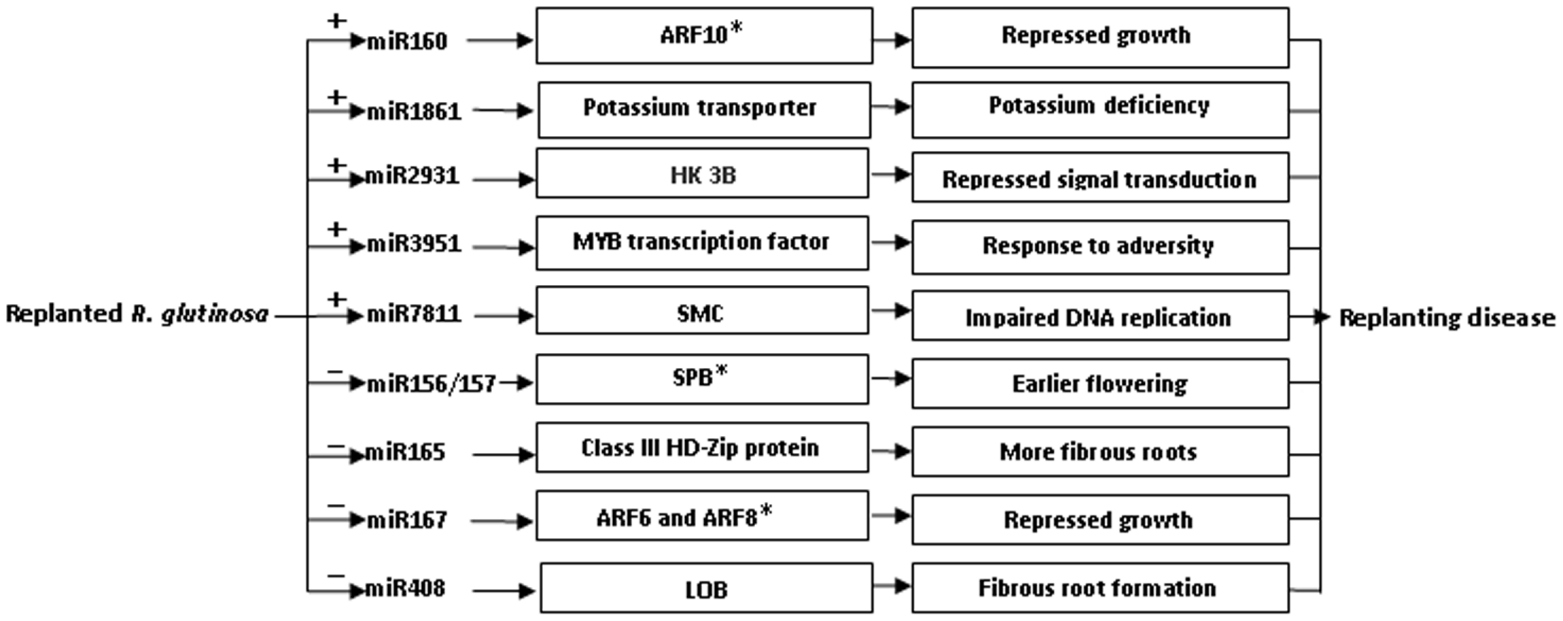
A hypothetical regulatory mechanism of miRNAs in replanted *R. glutinosa*. “+” positive regulation, “−” negative regulation, “*”, the result here is consistent with that of our previous study.

A hypothetical regulatory mechanism of miRNAs in the replanting disease is presented in Fig. 6. One of the targets of miR160 is an auxin response factor gene (*ARF10*), the product of which is heavily implicated in the development of the root cap [35]. If the higher abundance of miR160 in SP plants interfered with the production of ARF10, the expectation would be that a greater number of lateral roots would be formed. A target of miR1861 is the gene encoding potassium transporter 7, the product of which is required for, inter alia, the uptake of potassium from the soil [36], [37]. An excess presence of miR1861 in SP plants would thus be expected to compromise potassium uptake, and result in the abnormal expansion of tubers, a typical symptom of potassium deficiency. A target of miR2931 is a gene encoding histidine kinase 3B, which is a component of the eukaryotic machinery for signal transduction across the cellular membrane [38]. The down-regulation of this gene would therefore be expected to disturb normal signal transduction. A target of miRNA3951 is a gene encoding a MYB transcription factor, many of which act as major regulators of development, metabolism and the stress response [39], [40]. Its miRNA-driven down-regulation in SP plants may well therefore have major growth and developmental consequences. The target of miR7811 is a gene whose product is a member of the SMC (structural maintenance of chromosomes) family. Eukaryotic SMC proteins are the core components of the cohesin complex, which is responsible for sister chromatid and homolog cohesion during mitosis and meiosis [41]. Down-regulation of this gene in SP plants may therefore affect cell division. Other key targets of the miRNAs more abundant in SP than in FP included genes encoding a transducin family protein/WD-40 repeat family protein (miR1147), an ALY protein (miR1851) and an ABC transporter family protein (miR3512), all of which are key components of metabolism [42]–[44].

Among the targets of the miRNAs which were less abundant in the SP than in the FP plants were certain genes involved in the determination of flowering time, prematurity and the response to environmental stress. The product of genes encoding squamosa promoter-binding proteins (SPBs) (targeted by miR156/157) are known to regulate flowering time in *A. thaliana* and their over-expression accelerates flowering [12], [45], [46]. The reduced abundance of miR156/157 in SP plants implies the maintenance of a high level of SPB, which would tend to promote the shift to reproductive growth; the pleiotropic effect of this would be a reduction in vegetative and root growth. The genes targeted by miR165 included four which encode class III HD-Zip proteins; in the *A. thaliana* root meristem, these proteins are involved in xylem differentiation [47]. Their enhanced presence in SP plants might be expected to promote the formation of fibrous roots and to affect root expansion. miR167 targets genes encoding ARF6 and ARF8, which act as positive regulators of adventitious root formation [48], so an elevated presence of these proteins would tend to militate against the formation of tubers. miR408 targets a gene encoding a lateral organ boundary domain protein, which is important for lateral root morphogenesis in *Arabidopsis* [49], rice [50] and maize [51]. Since the abundance of miR408 was lower in SP than in FP plants, one might expect differences in the expression of the the *LOB* gene, leading to a greater degree of lateral root formation [52], with negative consequences for tuber formation. Several other targets of the reduced abundance miRNAs included a probable thiol methyltransferase 2 (miR1115), AGO 1 (miR168) and an RNA helicase (miR477); each of these gene products are components of the stress response [53], [54].

miRNAs regulate many cellular processes. Here, we have shown that they may well be involved in the replanting disease of *R. glutinosa*.

## Materials and Methods

### Plant Material and RNA Isolation

**Figure 7:**
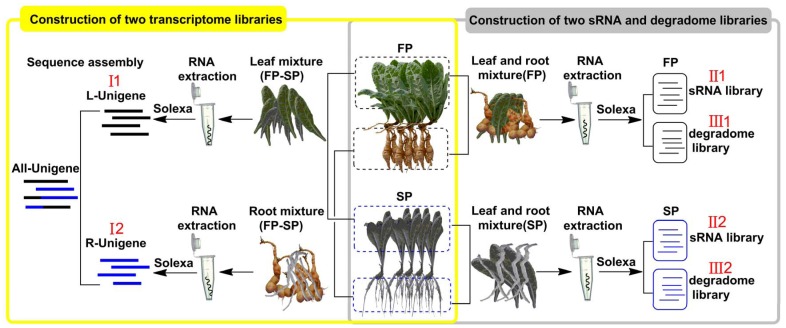
The schematic of *R. glutinosa* material composition for each library construction.

Our experimental plants of *R. glutinosa*, ‘Wen 85-5’, were grown in Wen Agricultural Institute, Jiaozuo City, Henan Province, China. The designed growing period was from the period from April 22, 2011 to November 30, 2011. A group of seedlings was grown in the field where *R. glutinosa* had not been planted for more than 10 years. The other group was grown in the field where the same cultivar had been grown in the previous year (planted on April 15, harvested on November 30, 2010). For convenience of description, we name the former group as first year plants (FP) and the latter group as second year plants (SP). *R. glutinosa* plants (five independent FP and same number SP) were collected at the tuberous root expansion stage (August 22, 2011). Six sequencing libraries belonging to three types (I, transcriptome, II, sRNA and III, degradome) were constructed in present study (Fig. 7), i.e. two transcriptome libraries, I1 (roots of FP and SP) and I2 (leaves of FP and SP), two sRNA libraries, II1 (roots and leaves of FP) and II2 (roots and leaves of SP), two degradome libraries, III1 (roots and leaves of FP) and III2 (roots and leaves of SP).

Total RNA from each sample was isolated using a TriZOL reagent (TaKaRa Co., Tokyo, Japan) following the manufacturer’s instructions and treated with RNase free DNase I (Qiagen). RNA concentrations were measured using a spectrophotometer and integrity was ensured through analysis on a 1.5% (w/v) agarose gel. Those RNA samples were also used in qRT-PCR verification.

For measure of miRNA different expression in different development stages, roots of FP and SP from five independent plants were collected every month from May 22 to October 22, 2011, and total RNAs were extracted with TriZOL reagent and subjected to qRT-PCR analysis.

### Transcriptome Sequencing and de novo Assembly Analysis

Beads with Oligo(dT) were used to isolate poly(A) mRNA after total RNA was collected from each sample. Fragmentation buffer was added for interrupting mRNA to short fragments. Taking these short fragments as templates, random hexamer-primer was used to synthesize the first-strand cDNA. The second-strand cDNA was synthesized using buffer, dNTPs, RNaseH and DNA polymerase I, respectively. Short fragments were purified with QiaQuick PCR extraction kit and resolved with EB buffer for end reparation and adding poly(A). After that, the short fragments are connected with sequencing adaptors. And, after the agarose gel electrophoresis, the suitable fragments are selected for the PCR amplification as templates. At last, the library could be sequenced using Illumina HiSeq™ 2000. Four fluorescently labeled nucleotides and a specialized polymerase were used to determine the clusters base by base in parallel. The raw reads were generated by the Illumina HiSeq™ 2000 system. Short reads after filtering dirty raw reads that only have 3′ adaptor fragments should be removed before data analysis, on which all following analysis are based. Transcriptome *de novo* assembly is carried out with short reads assembling program-SOAPdenovo [55]. If results of different databases conflict with each other, a priority order of (Nr) non-redundant protein, Swiss-Prot, KEGG and COG should be followed when deciding sequence direction of unigenes [15]. Genes were tentatively identified according to the best hits against known sequences.

### sRNA Sequencing and miRNAs Identification

To construct FP and SP sRNA libraries, total RNA from each plant was pooled, and then separated by 15% denaturing PAGE to recover the population of small RNAs (size range 18–30 nt) present. The small RNAs were ligated sequentially to 5′ and 3′ RNA/DNA chimeric oligonucleotide adaptors (Illumina), and the resulting ligation products were gel purified by 10% denaturing PAGE, and reverse transcribed. The cDNAs obtained in this way were sequenced by the Illumina HiSeq™ 2000 system. Known miRNAs were identified by Blastn searches against Genbank (www.ncbi.nlm.nih.gov), Rfam 10.1 (http://rfam.sanger.ac.uk/) and miRBase 19.0 (www.mirbase.org/) databases with default parameters (E-value cutoff was 10, Maximum no. of hits was 100, <3 mismatches nucleotides were allowed). Potentially novel sequences were identified by an alignment with the *R. glutinosa* transcriptome sequences using SOAP (http://soap.genomics.org.cn/) software [55]. Candidate pre-miRNAs were identified by folding the flanking sequences of distinct miRNAs using MIREAP (http://sourceforge.net/projects/mireap/), followed by a prediction of secondary structure by mFold v3.1 [56]. The criteria chosen for stem-loop hairpins were as follows [20], [21]: 100 nt maximum distance was allowed between miRNA and miRNA*, maximum free energy should be not less than 20 kcal mol^−1^, duplex asymmetry of miRNA and miRNA* was set in 7 nt, pairing number between miRNA and miRNA* was revised to 10 nt, mature bulge was less than 4 nt.

### Verification of Novel miRNAs and Analysis of miRNAs Different Expression

For reverse transcription (RT) reaction, polyA was first added to the 3′ end of the miRNAs using polyA polymerase, and cDNA was then synthesized using AMV reverse transcriptase (GeneCopoeia, Inc.), and then stored at −20°C, employing a 53 nt oligodT-adaptor sequence (GeneCopoeia, Inc.) as the primer.

For verification of novel miRNAs and analysis of miRNAs different expression, qRT-PCR was performed using an All-in-One™ miRNA Q-PCR detection kit (GeneCopoeia, Inc.) on a BIO-RAD iQ5 real-time PCR detection system (Bio-Rad laboratories, Inc.). Each 20 µl Q-PCR comprised 0.5 µl cDNA, 2 µl 2 µM miRNA forward primer (sequences given in Table G, H and I of File S1), 2 µl 2 µM reverse primer from a 53 nt oligodT-adaptor sequence (Universal Adaptor PCR Primer), 10 µl 2× All-in-One™ miRNA Q-PCR buffer and 5.5 µl nuclease-free water. The reactions were incubated at 95°C for 10 min, and then were cycled 36 times through 95°C/10s, 55°C/20s and 72°C/10s. After the reactions had been completed, the threshold was manually set and the threshold cycle (CT) was automatically recorded. 3 technical replicates were used for each tested sample. A 4 µl aliquot of each reaction product was subjected to 3% agarose electrophoresis. The relative expression level of the miRNAs was calculated using the 2^−△△CT^ method [57], and the data were normalized on the basis of 18s rRNA CT values.

### Degradome Sequencing, Target Identification and Analysis

FP and SP degradome libraries were constructed according to a published protocol [22], [58]. Briefly, RNA fragments with a poly(A) tail were isolated from total RNA of each example using the Oligotex mRNA mimi kit (Qiagen), and then a 5′ RNA adaptor with a *Mme*I restriction site at its 3′ end was added to the 5′ ends of the isolated poly(A) RNAs. After reverse transcription using oligo d(T) and PCR enrichment, the PCR products were purified and digested with *Mme*I. After ligating a double-stranded DNA adaptor to the 3′ end of the digested products, the ligated products were further purified and amplified, and then sequenced using the Illumina HiSeq™ 2000 system.

Raw sequencing reads were obtained using Illumina’s Pipeline v1.5 software to remove adaptor sequences and low quality sequencing reads. The extracted sequencing reads with the length of 20 and 21 nt were then used to identify potentially cleaved targets by the CleaveLand pipeline [22], [23]. The degradome reads were mapped to the *R. glutinosa* transcriptome sequences. The target was selected categorized as I, II and III as previous study [19], [22]. In addition, to easily analyze the miRNA targets and RNA degradation patterns, t-plots were built according to the distribution of signatures (and abundances) along the *R. glutinosa* transcriptome. All the identified targets were subjected to BlastX analysis (http://www.ncbi.nlm.nih.gov/BLAST/) to search for similarity, and then to GO analysis previously described [59].

### Statistical Analysis

Each result in this study is the mean of at least three replicated treatments and each treatment contained at least five roots, leaves and plants (including roots and leaves), respectively. Statistical analysis was performed to identify differentially expressed sRNAs between the libraries using a rigorous algorithm described previously [60]. For small RNAs, the SP (replant or allelopathy autotoxicity-stress) library-derived sequence reads were normalized to the high-quality reads of the control (the second plant, FP) library. The absolute value of log_2_ ratio≤1 was used as the threshold to judge the significant difference of miRNA expression [32].

## Supporting Information

File S1 **Additional tables.**
(DOC)

File S2 **Additional figures.**
(DOC)

File S3 **Secondary structure of novel miRNAs from** ***R. glutinosa*** **.**
(DOC)

File S4 **Target plots (t-plots) of selected 8 up- (A–H) and 8 down-regulated (I–P) miRNAs confirmed from the SP and FP degradome libraries, respectively.** Note: T-plot (top) and miRNA: mRNA alignments (bottom).
(DOC)

## References

[1] Wen XS, Yang SL, Wei JH, Zheng JH (2002) Textual research on planting history of *Rehmannia glutinosa* and its cultivated varieties. Chinese Traditional and Herbal Drugs 33: 946–949 (in Chinese)..

[2] Zhao YJ, Chen Z (1991) Effect of N, P and K supply on dry matter accumulation and nutrient contents of *Rehmannia glutinosa Libosch* . Jorunal of Chinese Medicinal Materials 14: 3–6 (in Chinese)..

[3] Yu FP, Yang L (1994) Preliminary study of *Rehmannia* mosaic virus. Acta Phytopathologica Sinica 24: 310–310 (in Chinese)..

[4] Du JF, Yin WJ, Zhang ZY, Hou J, Huang J, et al (2009) Autotoxicity and phenolic acids content in soils with different planting interval years of *Rehmannia glutinosa* . Chinese Journal of Ecology 28: 445–450 (in Chinese)..

[5] Yang YH, Zhang ZY, Fan HM, Zhao YD, Li MJ, et al. (2012) Construction and analysis of a different expression cDNA library in *Rehmannia glutinosa* plants subjected to continuous cropping. Acta Physiology plant DOI: 10.1007/s11738-012-1105-9.

[6] Li ZF, Yang YQ, Xie DF, Zhu LF, Zhang ZG, et al. (2012) Identification of autotoxic compounds in fibrous roots of Rehmannia *(Rehmannia glutinosa* Libosch.). PLoSOne 7, e28806. 10.1371/journal.pone.0028806 PMC3250401 22235251

[7] Reinhart BJ, Weinstein EG, Rhoades MW, Bartel B, Bartel DP (2002) MicroRNAs in plants. Genes & Development 16, 1616–1626. 10.1101/gad.1004402 PMC186362 12101121

[8] Bartel DP (2004) MicroRNAs: genomics, biogenesis, mechanism, and function. Cell 116, 281–297. 10.1016/s0092-8674(04)00045-5 14744438

[9] Lu S, Sun YH, Shi R, Clark C, Li L, et al. (2005) Novel and mechanical stress-responsive MicroRNAs in *Populus trichocarpa* that are absent from *Arabidopsis*. Plant Cell 17, 2186–2203. 10.1105/tpc.105.033456 PMC1182482 15994906

[10] Tang X, Bian S, Tang M, Lu Q, Li S, et al. (2012) MicroRNA-Mediated Repression of the Seed Maturation Program during Vegetative Development in *Arabidopsis*. PLoS Genetics 8, e1003091. 10.1371/journal.pgen.1003091 PMC3510056 23209442

[11] Zhan X, Wang B, Li H, Liu R, Kalia RK, et al. (2012) *Arabidopsis* proline-rich protein important for development and abiotic stress tolerance is involved in microRNA biogenesis. Proc Natl Acad Sci USA 109, 18198–18203. 10.1073/pnas.1216199109 PMC3497810 23071326

[12] Wang JW, Czech B, Weigel D (2009) MiR156-regulated SPL transcription factors define an endogenous flowering pathway in *Arabidopsis thaliana*. Cell 138, 738–749. 10.1016/j.cell.2009.06.014 19703399

[13] Parchman TL, Geist KS, Grahnen JA, Benkman CW, Buerkle CA (2010) Transcriptome sequencing in an ecologically important tree species: assembly, annotation, and marker discovery. BMC Genomics doi: 10.1186/1471-2164-11-180. 10.1186/1471-2164-11-180 PMC2851599 20233449

[14] Liu S, Li D, Li Q, Zhao P, Xiang Z, et al.. (2010) MicroRNAs of Bombyx mori identified by Solexa sequencing. BMC Genomics 11, 148–160. 10.1186/1471-2164-11-148 PMC2838851 20199675

[15] Xiang LX, He D, Dong WR, Zhang YW, Shao JZ (2010) Deep sequencing-based transcriptome profiling analysis of bacteria-challenged *Lateolabrax japonicus* reveals insight into the immune-relevant genes in marine fish. BMC Genomics Doi: 10.1186/1471-2164-11-472. 10.1186/1471-2164-11-472 PMC3091668 20707909

[16] Wang Z, Zhang J, Jia C, Liu J, Li Y, et al. (2012) *De Novo* characterization of the banana root transcriptome and analysis of gene expression under *Fusarium oxysporum f. sp.* Cubense tropical race 4 infection. BMC Genomics 13, 650–662. 10.1186/1471-2164-13-650 PMC3534498 23170772

[17] Xu DL, Long H, Liang JJ, Zhang J, Chen X, et al. (2012) *De novo* assembly and characterization of the root transcriptome of Aegilops variabilis during an interaction with the cereal cyst nematode. BMC Genomics doi: 10.1186/1471-2164-13-133. 10.1186/1471-2164-13-133 PMC3439707 22494814

[18] Yang Y, Chen X, Chen J, Xu H, Li J, et al. (2011) Differential miRNA expression in *Rehmannia glutinosa* plants subjected to continuous cropping. BMC Plant Bioligy doi:10.1186/1471-2229-11-53. 10.1186/1471-2229-11-53 PMC3078876 21439075

[19] Zhou ZS, Zeng HQ, Liu ZP, Yang ZM (2012) Genome-wide identification of Medicago truncatula microRNAs and their targets reveals their differential regulation by heavy metal. Plant, Cell & Environment 35, 86–99. 10.1111/j.1365-3040.2011.02418.x 21895696

[20] Jones-Rhoades MW, Bartel DP, Bartel B (2006) MicroRNAs and their regulatory roles in plants. Annual Review of Plant Biology 57, 19–53. 10.1146/annurev.arplant.57.032905.105218 16669754

[21] Meyers BC, Axtell MJ, Bartel B, Bartel DP, Baulcombe D (2008) Criteria for annotation of plant microRNAs. The Plant Cell 20, 3186–3190. 10.1105/tpc.108.064311 PMC2630443 19074682

[22] Addo-Quaye C, Eshoo TW, Bartel DP, Axtell MJ (2008) Endogenous siRNA and miRNA targets identified by sequencing of the *Arabidopsis* degradome. Current Biology 8, 758–762. 10.1016/j.cub.2008.04.042 PMC2583427 18472421

[23] Addo-Quaye C, Miller W, Axtell MJ (2009) CleaveLand, a pipeline for using degradome data to find cleaved small RNA targets. Bioinformatics. 25, 130–131. 10.1093/bioinformatics/btn604 PMC3202307 19017659

[24] Yang Y, Chen X, Chen J, Xu H, Li J, et al. (2011) Identification of novel and conserved microRNAs in *Rehmannia glutinosa* by Solexa sequencing. Plant Molecular Biology Report 29, 986–996.

[25] Szittya G, Moxon S, Santos DM, Jing R, Fevereiro MP, et al.. (2008) High-throughput sequencing of Medicago truncatula short RNAs identifies eight new miRNA families. BMC Genomics 9, 593–601. 10.1186/1471-2164-9-593 PMC2621214 19068109

[26] Kwak PB, Wang QQ, Chen XS, Qiu CX, Yang ZM (2009) Enrichment of a set of microRNAs during the cotton fiber development. BMC Genomics 10, 457–467. 10.1186/1471-2164-10-457 PMC2760587 19788742

[27] Zhao CZ, Xia H, Frazier TP, Yao YY, Bi YP, et al. (2010) Deep sequencing identifies novel and conserved microRNAs in peanuts (*Arachis hypogaea* L.) BMC Plant Biology 10, 3–14. 10.1186/1471-2229-10-3 PMC2826338 20047695

[28] Kim J, Park JH, Lim CJ, Lim JY, Ryu JY, et al.. (2012) Small RNA and transcriptome deep sequencing proffers insight into floral gene regulation in Rosa cultivars. BMC Genomics 13, 657. 10.1186/1471-2164-13-657 PMC3527192 23171001

[29] Ruan MB, Zhao YT, Meng ZH, Wang XJ, Yang WC (2009) Conserved miRNA analysis in *Gossypium hirsutum* through small RNA sequencing. Genomics. 94, 263–268. 10.1016/j.ygeno.2009.07.002 19628031

[30] Xu Q, Liu Y, Zhu A, Wu X, Ye J, et al.. (2010) Discovery and comparative profiling of microRNAs in a sweet orange red-flesh mutant and its wild type. BMC Genomics 11, 246–252. 10.1186/1471-2164-11-246 PMC2864249 20398412

[31] Kawashima CG, Yoshimoto N, Maruyama-Nakashita A, Tsuchiya YN, Saito K, et al.. (2009) Sulphur starvation induces the expression of microRNA-395 and one of its target genes but in different cell types. The Plant Journal 57, 313–321. 10.1111/j.1365-313X.2008.03690.x 18801012

[32] Zhou ZS, Zeng HQ, Liu ZP, Yang ZM (2012) Genome-wide identification of *Medicago truncatula* microRNAs and their targets reveals their differential regulation by heavy metal. Plant Cell & Environment 35, 86–99. 10.1111/j.1365-3040.2011.02418.x 21895696

[33] Brodersen P, Voinnet O (2006) The diversity of RNA silencing pathways in plants. Trends Genet 22, 268–280. 10.1016/j.tig.2006.03.003 16567016

[34] Li XE, Chen SL, Wei SQ, Wei JH, Lan J. (2006) Analysis on adaptive area of *Rehmannia glutinosa* L. and it’s class partition. China Journal of Chinese Materia Medica. 31, 344–346 (in Chinese).

[35] Wang JW, Wang LJ, Mao YB, Cai WJ, Xue HW, et al. (2005) Control of root cap formation by MicroRNA-targeted auxin response factors in *Arabidopsis*. Plant Cell 17, 2204–2216. 10.1105/tpc.105.033076 PMC1182483 16006581

[36] Ashley MK, Grant M, Grabov A (2006) Plant responses to potassium deficiencies: a role for potassium transport proteins. Journal of Experimental Botany 7, 425–436. 10.1093/jxb/erj034 16364949

[37] Grabov A (2007) Plant KT/KUP/HAK Potassium Transporters: Single Family – Multiple Functions. Annals of Botany 99, 1035–1041. 10.1093/aob/mcm066 PMC3243584 17495982

[38] Bilwes AM, Quezada CM, Croal LR, Crane BR, Simon MI (2001) Nucleotide binding by the histidine kinase CheA. Natural Structural Biology 8, 353–360. 10.1038/86243 11276258

[39] Dubos C, Stracke R, Grotewold E, Weisshaar B, Martin C, et al. (2010) MYB transcription factors in *Arabidopsis*. Cell 15, 1360–1385. 10.1016/j.tplants.2010.06.005 20674465

[40] "Chagne D, Lin-Wang K, Espley RV, Volz RK, How NM, et al.. (2012) An ancient duplication of apple MYB transcription factors is responsible for novel red fruit-flesh phenotypes. Plant Physiology doi: http://dx.doi.org/10.1104/pp.112. 10.1104/pp.112.206771 PMC3532254 23096157

[41] Pavlova SV, Zakiian SM (2003) SMC (structural maintenance of chromosomes) structural protein family and their role in chromatin reorganization. Genetika 39, 1301–1316. 14658334

[42] Shi DQ, Liu J, Xiang YH, Ye D, Sundaresan V, et al. (2005) SLOW WALKER1, essential for gametogenesis in *Arabidopsis*, encodes a WD40 protein involved in 18 S ribosomal RNA biogenesis. Plant Cell 17, 2340–2354. 10.1105/tpc.105.033563 PMC1182493 15980260

[43] Zhou Z, Luo M, Straesser K, Katahira J, Hurt E, et al.. (2000) The protein Aly links pre-messenger-RNA splicing to nuclear export in metazoans. Nature 407, 401–405. 10.1038/35030160 11014198

[44] Bruhn L, Munnerlyn A, Grosschedl R (2012) ALY, a context-dependent coactivator of LEF-1 and AML-1 is required for TCRα enhancer function. Genes & Development 11, 640–653. 10.1101/gad.11.5.640 9119228

[45] Xie K, Wu C, Xiong L (2006) Genomic organization, differential expression and interaction of SQUAMOSA promoter-binding-like transcription factors and microRNA156 in rice. Plant Physiology 142, 280–293. 10.1104/pp.106.084475 PMC1557610 16861571

[46] Wu G, Poething RS (2006) Temporal regulation of shoot development in *Arabidopsis thaliana* by miR156 and its target SPL3. Development 133, 3539–3547. 10.1242/dev.02521 PMC1610107 16914499

[47] Miyashima S, Koi S, Hashimoto T, Nakajima K (2011) Non-cell-autonomous microRNA165 acts in a dose-dependent manner to regulate multiple differentiation status in the *Arabidopsis* root. Development 138, 2303–2313. 10.1242/dev.060491 21558378

[48] Gutierrez L, Bussell JD, Pacurar DI, Schwambach J, Pacurar M, et al. (2009) Phenotypic plasticity of adventitious rooting in *Arabidopsis* is controlled by complex regulation of AUXIN RESPONSE FACTOR transcripts and microRNA abundance. Plant cell 21, 3119–3132. 10.1105/tpc.108.064758 PMC2782293 19820192

[49] Okushima Y, Overvoorde PJ, Arima K, Alonso JM, Chan A, et al. (2005) Functional genomics analysis of the AUXIN RESPONSE FACTOR gene family members in *Arabidopsis thaliana*: Unique and overlapping functions of ARF7 and ARF19. Plant Cell 17, 444–463. 10.1105/tpc.104.028316 PMC548818 15659631

[50] Inukai Y, Sakamoto T, Ueguchi-Tanaka M, Shibata Y, Gomi K, et al.. (2005) Crown rootless1, which is essential for crown root formation in rice, is a target of an AUXIN RESPONSE FACTOR in auxin signaling. Plant Cell 17, 1387–1396. 10.1105/tpc.105.030981 PMC1091762 15829602

[51] Bortiri E, Chuck G, Vollbrecht E, Rocheford T, Martienssen R, et al.. (2006) ramosa2 encodes a LATERAL ORGAN BOUNDARY domain protein that determines the fate of stem cells in branch meristems of maize. Plant Cell 18, 574–585. 10.1105/tpc.105.039032 PMC1383634 16399802

[52] Cao ZY, Geng BB, Xu S, Xuan W, Nie L, et al. (2011). BnHO1, a haem oxygenase-1 gene from *Brassica napus*, is required for salinity and osmotic stress-induced lateral root formation. Journal of Experimental Botany 62, 4675–4689. 10.1093/jxb/err190 PMC3170560 21673093

[53] Gazzani S, Li M, Maistri S, Scarponi E, Graziola M, et al.. (2009) Evolution of MIR168 paralogs in Brassicaceae. BMC Evolutionary Biology 9, 62. doi: 10.1186/1471-2148-9-62. 10.1186/1471-2148-9-62 PMC2664809 19309501

[54] Nagatoshi Y, Nakamura T (2009) *Arabidopsis* HARMLESS TO OZONE LAYER protein methylates a glucosinolate breakdown product and functions in resistance to Pseudomonas syringae pv. maculicola. The Journal Biological Chemistry 284, 19301–19309. 10.1074/jbc.M109.001032 PMC2740555 19419967

[55] Li R, Li Y, Kristiansen K, Wang J (2008) SOAP: short oligonucleotide alignment program. Bioinformatics 24, 713–714. 10.1093/bioinformatics/btn025 18227114

[56] Zuker M (2003) Mfold web server for nucleic acid folding and hybridization prediction. Nucleic Acids Research 31, 3406–3415. 10.1093/nar/gkg595 PMC169194 12824337

[57] Livak KJ, Schmittgen TD (2001) Analysis of relative gene expression data using real-time quantitative PCR and the 2 (-Delta Delta C (T)). Method 25, 402–408. 10.1006/meth.2001.1262 11846609

[58] German MA, Pillay M, Jeong DH, Hetawal A, Luo S, et al.. (2008) Global identification of microRNA-target RNA pairs by parallel analysis of RNA ends. Nature Biotechnology 26, 941–946. 10.1038/nbt1417 18542052

[59] Xie FL, Frazier TP, Zhang BH (2011) Identification, characterization and expression analysis of MicroRNAs and their targets in the potato (*Solanum tuberosum*). Gene 473, 8–22. 10.1016/j.gene.2010.09.007 20933063

[60] Audic S, Claverie JM (1997) The significance of digital gene expression profiles. Genome Research 7, 986–995. 10.1101/gr.7.10.986 9331369

