# Supplementary material for: Transcriptome/Degradome-Wide Identification of R. glutinosa miRNAs and Their Targets: The Role of miRNA Activity in the Replanting Disease
Source: PLoS One. 2013 Jul 5;8(7):e68531. doi: 10.1371/journal.pone.0068531 (PMC3702588; doi:10.1371/journal.pone.0068531)
Supplement: File S1 — Additional tables. (DOC) [file pone.0068531.s001.doc]

**File S1. Additional tables**

| Table A. Statistics of root and leaf reads data production. | | | |  |  |
| --- | --- | --- | --- | --- | --- |
| Samples | Total Reads | Total Nucleotides (nt) | Q20 percentage | N percentage | GC percentage |
| Root | 40,633,336 | 3,657,000,240 | 94.31 | 0.01 | 45.30 |
| Leaf | 41,400,002 | 3,726,000,180 | 94.54 | 0.00 | 46.58 |

| Table B. Statistics of reads data production from FP and SP libraries. | | |
| --- | --- | --- |
| **Read nubmer** | **FP** | **SP** |
| Raw reads | 17,723,851 | 18,123,606 |
| Removal reads (<18nt) | 3,064,873 | 2,473,742 |
| Clean reads | 14,658,978 | 15,649,864 |
| Unique clean reads | 6,798,635 | 7,917,831 |

| Table C. All known (conserved, non-conserved and *Rehmannia*-specific miRNAs) and their transcript abundance identified from FP and SP libraries. | | | | | | | | | |
| --- | --- | --- | --- | --- | --- | --- | --- | --- | --- |
| **miRNA family** | **miR-name** | **Length (bp)** | **Sequences** | **miRNA reads** | | **miRNA family abundance** | | **Percentage of each miRNA family reads in a library total reads (%)** | |
|  |  |  |  | **FP** | **SP** | **FP** | **SP** | **FP** | **SP** |
| **Conserved (Group I)** | | | | | | | | | |
| miR156 | miR156a | 20 | UGACAGAAGAGAGUGAGCAU | 13,778 | 6,893 | 14,969 | 7,207 | 2.77 | 1.34 |
|  | miR156b | 22 | CACGACAGAUAGAAAGCACAAU | 860 | 123 |  |  |  |  |
|  | miR156c | 21 | UUGACAGAAGAGAGAGAGCAC | 164 | 99 |  |  |  |  |
|  | miR156d | 22 | UGCUCAUUUCUCUUUCUGUCAG | 44 | 10 |  |  |  |  |
|  | miR156e | 22 | UUGACAGAAGAUAGAGAGCACA | 36 | 25 |  |  |  |  |
|  | miR156f | 21 | UUGACAGAAGAUAGAGGGCAC | 33 | 26 |  |  |  |  |
|  | miR156g | 20 | UGACAGAAGAGAGAGAGCAU | 22 | 7 |  |  |  |  |
|  | miR156h | 21 | UGACAGAAGAUAGAGAGCACA | 20 | 16 |  |  |  |  |
|  | miR156i | 21 | CUGACAGAAGAGAGUGAGCAU | 12 | 8 |  |  |  |  |
| miR157 | miR157a | 21 | UUGACAGAAGAUAGAGAGCAC | 136,153 | 27,475 | 138,648 | 28,060 | 25.68 | 5.20 |
|  | miR157b | 20 | UGACAGAAGAUAGAGAGCAC | 2,049 | 408 |  |  |  |  |
|  | miR157c | 21 | GCUCUCUAGUCUUCUGUCAUC | 406 | 143 |  |  |  |  |
|  | miR157d | 20 | UGACAGAAGAUAGAAAGCAC | 40 | 34 |  |  |  |  |
| miR158 | miR158a | 20 | UCCCAAAUGUAGACAAAGCA | 25 | 140 | 27 | 149 | 0.01 | 0.03 |
|  | miR158b | 22 | UUUGAAUCGGCAAUUUUGGAAA | 2 | 9 |  |  |  |  |
| miR159 | miR159a | 21 | UUUGGAUUGAAGGGAGCUCUA | 4,146 | 7,024 | 4,850 | 7,659 | 0.90 | 2.42 |
|  | miR159b | 21 | GAGCUCCUUGAAGUCCAAUAG | 633 | 845 |  |  |  |  |
|  | miR159c | 21 | GAGCUCCUUGAAGUCCAAUCG | 56 | 67 |  |  |  |  |
|  | miR159d | 21 | UUUGAGUGAAGGGCGUUUCUG | 15 | 23 |  |  |  |  |
| miR160 | miR160a | 21 | GCGUAUGAGGAGCGAAGCAUA | 328 | 1,476 | 718 | 2,102 | 0.13 | 0.67 |
|  | miR160b | 21 | GCGUACGAGGAGCCAAGCAUG | 324 | 453 |  |  |  |  |
|  | miR160c | 21 | GUGUACGAGGAGCCAAGCAUG | 28 | 126 |  |  |  |  |
|  | miR160d | 21 | GCGUACGUGGAGCCAAGCAUG | 20 | 26 |  |  |  |  |
|  | miR160e | 21 | GCGUACGAGGUGCCAAGCAUG | 18 | 21 |  |  |  |  |
| miR161 | miR161a | 23 | UCAAUGCAACUGAAAGAUACAUA | 4 | 13 | 8 | 22 | 0.00 | 0.01 |
|  | miR161b | 21 | UUGAAAGUGACUACAUCGGGG | 2 | 6 |  |  |  |  |
|  | miR161c | 24 | UCAAGCUAUUAGAGAAGUGACUAU | 1 | 3 |  |  |  |  |
| miR162 | miR162 | 21 | UCGAUAAGCCUCUGCAUCCAG | 1 | 0 |  |  |  |  |
| miR163 | miR163a | 22 | UUGUAAGAGACUUGGAGCUUAU | 42 | 67 | 49 | 130 | 0.01 | 0.04 |
|  | miR163b | 24 | UGAAGAGGGACUGCAUCUCGAUCA | 5 | 63 |  |  |  |  |
| miR164 | miR164a | 21 | UGGAGAAGCAGGGCACGUGCA | 7,966 | 5,845 | 10,513 | 7,702 | 1.95 | 2.44 |
|  | miR164b | 21 | CAUGUGCCCAUCUUCCCCAUC | 2,013 | 1,491 |  |  |  |  |
|  | miR164c | 21 | CAUGUGCUCUUCUUCCCCAUC | 463 | 316 |  |  |  |  |
|  | miR164d | 21 | UGGAGAAGCAGGGCACGUGCU | 59 | 41 |  |  |  |  |
|  | miR164e | 22 | UGGAGAAUGGAGGCGCACAUGU | 12 | 9 |  |  |  |  |
| miR165 | miR165a | 21 | UCGGACCAGGCUUCAUCCCCC | 91 | 39 | 118 | 48 | 0.02 | 0.02 |
|  | miR165b | 19 | GAAGUGUUCGGAUCGAGGC | 27 | 9 |  |  |  |  |
| miR166 | miR166a | 21 | UCGGACCAGGCUUCAUUCCCC | 62,457 | 36,859 | 75,898 | 47,545 | 14.06 | 15.04 |
|  | miR166b | 21 | UCGGACCAGGCUUCAUUCCUC | 12,356 | 9,873 |  |  |  |  |
|  | miR166c | 21 | GGAAUGUUGUCUGGCUCGAGG | 568 | 423 |  |  |  |  |
|  | miR166d | 19 | GGACCAGGCUUCAUUCCUC | 264 | 187 |  |  |  |  |
|  | miR166e | 18 | UCGGACCAGGCUCCAUUC | 123 | 99 |  |  |  |  |
|  | miR166f | 21 | UCUCGGACCAGGCUUCAUUCC | 107 | 86 |  |  |  |  |
|  | miR166g | 21 | GGGAUGUUGGCUGGCUCGACG | 23 | 18 |  |  |  |  |
| miR167 | miR167a | 21 | UGAAGCUGCCAGCAUGAUCUGG | 18,234 | 6,686 | 20,874 | 7,931 | 3.87 | 2.51 |
|  | miR167b | 23 | GGUCAUGCUCUGACAGCAUCACU | 968 | 402 |  |  |  |  |
|  | miR167c | 21 | UGAAGCUGCCAGCAUGAUCUC | 654 | 487 |  |  |  |  |
|  | miR167d | 21 | GUUCUAGUACGACCGUCGAAU | 498 | 237 |  |  |  |  |
|  | miR167e | 20 | GAUCAUGUGGUAGCUUCAUC | 445 | 58 |  |  |  |  |
|  | miR167f | 22 | UGAAGCUGCCAGCAUGAUCUCA | 49 | 42 |  |  |  |  |
|  | miR167g | 22 | UGAAGCUGCCAGCAUGAUCUGG | 26 | 19 |  |  |  |  |
| miR168 | miR168 | 21 | UCGCUUGGUGCAGGUCGGGAA | 5,613 | 1,188 | 5,613 | 1,188 | 1.04 | 0.38 |
| miR169 | miR169a | 20 | UAGCCAAGGAUUUUUGCGUG | 447 | 556 | 1,211 | 1,409 | 0.22 | 0.45 |
|  | miR169b | 21 | CAGCCAAGGAUGACUUGCCGG | 345 | 359 |  |  |  |  |
|  | miR169c | 21 | UAGCCAAGGAUGACUUGCCGG | 133 | 148 |  |  |  |  |
|  | miR169d | 20 | GGCGUCAUCCGUUGGCUAUC | 102 | 113 |  |  |  |  |
|  | miR169e | 20 | UAGCCAACGGAUGACGCCAA | 46 | 59 |  |  |  |  |
|  | miR169f | 20 | GGCGUCAUCCAUUGGCUAUC | 47 | 58 |  |  |  |  |
|  | miR169g | 21 | UAGCCAAGAAUGACUUGCCUG | 37 | 44 |  |  |  |  |
|  | miR169h | 21 | UAGCCAGGGAUGAUUUGCCGG | 21 | 26 |  |  |  |  |
|  | miR169i | 19 | GCAAGUUGUCUUUGGCUAU | 16 | 25 |  |  |  |  |
|  | miR169j | 21 | CAUGCCAGGACUGAUUUGCGG | 17 | 21 |  |  |  |  |
| miR170 | miR170 | 21 | AUAUAGGCCUAGUUCACUCAG | 145 | 438 | 145 | 438 | 0.03 | 0.14 |
| miR171 | miR171a | 21 | UGUUGGAACGGCUCAAUCAAA | 1,046 | 987 | 2,207 | 2,015 | 0.41 | 0.64 |
|  | miR171b | 21 | UGAUUGAGCCGUGCCAAUAUC | 468 | 442 |  |  |  |  |
|  | miR171c | 21 | AGAUAUUGGUGCGGUUCAAUU | 272 | 214 |  |  |  |  |
|  | miR171d | 21 | UGUUGGCUCGGCUCACUCAGA | 231 | 199 |  |  |  |  |
|  | miR171e | 18 | UAAUGAACCGAGGCAAAU | 164 | 138 |  |  |  |  |
|  | miR171f | 21 | AGGAUUGAGCCGCGUUUAAUC | 26 | 35 |  |  |  |  |
| miR172 | miR172a | 21 | AGAAUCUUGAUGAUGCUGCAU | 55,394 | 41,202 | 61,728 | 48,364 | 11.43 | 15.30 |
|  | miR172b | 20 | GAAUCUUGAUGAUGCUGCAU | 4,122 | 3,959 |  |  |  |  |
|  | miR172c | 21 | AGAAUCCUGAUGAUGCUGCAU | 1,756 | 2,824 |  |  |  |  |
|  | miR172d | 21 | CGCAUCUUGUUGAUGCUGCAU | 456 | 379 |  |  |  |  |
| miR390 | miR390a | 21 | AAGCUCAGGAGGGAUAGCGCC | 355 | 406 | 755 | 727 | 0.14 | 0.23 |
|  | miR390b | 21 | CGCUAUCCAUCCUGAGUUUCA | 300 | 321 |  |  |  |  |
| miR393 | miR393a | 21 | UCAUAAAGGGUUGCAUUGAUC | 12 | 7 | 20 | 13 | 0.00 | 0.00 |
|  | miR393b | 23 | CGCUAUCCAUCCUGAGUUUCAAU | 2 | 1 |  |  |  |  |
|  | miR393c | 21 | AACUUUGUGACUGAAAAAGAG | 2 | 2 |  |  |  |  |
|  | miR393d | 23 | AUCAUGCGAUCCCUUAGGAAU | 1 | 1 |  |  |  |  |
|  | miR393e | 21 | UCAUAAAGGGUUGCAUUGAUC | 2 | 1 |  |  |  |  |
|  | miR393f | 21 | AUCCAGAAGGAAUGAUUGAUC | 1 | 1 |  |  |  |  |
| miR394 | miR394a | 20 | UUGGCAUUCUGUCCACCUCC | 1,237 | 1,496 | 1,463 | 1,783 | 0.27 | 0.56 |
|  | miR394b | 21 | AGGUGGGCAUACUGCCAAAUG | 146 | 187 |  |  |  |  |
|  | miR394c | 20 | AGGUGGGCAUACUGCCAAAU | 45 | 54 |  |  |  |  |
|  | miR394d | 21 | UUGGCAUUCUGUCCACCUCCU | 23 | 26 |  |  |  |  |
|  | miR394e | 21 | AGGUGGAGACAUGUGUCAACU | 12 | 20 |  |  |  |  |
| miR395 | miR395a | 20 | GUGAAGAUUUGGUGGGAACU | 2,076 | 2,079 | 2,648 | 2,723 | 0.49 | 0.86 |
|  | miR395b | 19 | UGAAGAUUUGGUGGGAACU | 316 | 388 |  |  |  |  |
|  | miR395c | 21 | CUGAAGUGUUUGGGGGAACUC | 123 | 126 |  |  |  |  |
|  | miR395d | 23 | CUGAAGAGUUCUGGAAGAUCUGC | 59 | 65 |  |  |  |  |
|  | miR395e | 24 | GGUGAAGAUUUGGCGGAAACUCAU | 26 | 24 |  |  |  |  |
|  | miR395f | 22 | GGUGAAGUUUUGGUGGGAACUC | 24 | 18 |  |  |  |  |
|  | miR395g | 23 | AGUGACGUGUUUGUGAGUAACUC | 16 | 10 |  |  |  |  |
|  | miR395g | 21 | AUGAAGAUUUGGUGGAAACUC | 8 | 13 |  |  |  |  |
| miR396 | miR396a | 21 | UUCCACAGCUUUCUUGAACUG | 1,074 | 673 | 2,148 | 1,346 | 0.40 | 0.43 |
|  | miR396b | 21 | UUCCACAGCUUUCUUGAACUU | 587 | 366 |  |  |  |  |
|  | miR396c | 20 | UUCAAGAAAGCUGUGGGAAG | 397 | 248 |  |  |  |  |
|  | miR396d | 21 | GUUCAAUAAAGCUGUGGGAAG | 31 | 19 |  |  |  |  |
|  | miR396e | 21 | AGUUCAAGAAAGCUGUGGGAA | 31 | 20 |  |  |  |  |
|  | miR396f | 21 | GUUCAAUCAAGCUGUGGGAAG | 19 | 12 |  |  |  |  |
|  | miR396g | 21 | GUUCAAGAAAGCUGUGGGAAG | 9 | 8 |  |  |  |  |
| miR397 | miR397a | 21 | UUGAGUGCAGCGUUGAUGAUA | 99 | 2 | 151 | 14 | 0.03 | 0.00 |
|  | miR397c | 21 | UCAUUGAGUGCAGCGUUGAUG | 48 | 11 |  |  |  |  |
|  | miR397b | 20 | GUCGUUGAGUGCAGCGUUGA | 4 | 1 |  |  |  |  |
| miR398 | miR398a | 21 | GGGGCAACAUGAGAACAUAUA | 329 | 21 | 498 | 150 | 0.09 | 0.05 |
|  | miR398b | 24 | GUGGAGUGUCCAUAGAACAUCGGA | 75 | 56 |  |  |  |  |
|  | miR398c | 21 | CGAUUGAUAUAGAGGACACUG | 63 | 72 |  |  |  |  |
|  | miR398d | 24 | GCAGUAGUCAUAUGAGAACACGGA | 23 | 0 |  |  |  |  |
|  | miR398e | 20 | AGGGGCGACCUGAGAACAUG | 8 | 1 |  |  |  |  |
| miR399 | miR399a | 21 | UGCCAAAGGAGAGUUGCCCUA | 36 | 67 | 110 | 126 | 0.02 | 0.04 |
|  | miR399b | 21 | GCCAAAGGAGAGUUGCCCUAA | 23 | 18 |  |  |  |  |
|  | miR399c | 24 | UGACCAAGAGGAGAUUGCCCUGAU | 15 | 12 |  |  |  |  |
|  | miR399d | 20 | UGCCAAAGGAGAGUUGCCCU | 11 | 10 |  |  |  |  |
|  | miR399e | 22 | UGCCAAAGGUAGCAGCUCCCAA | 8 | 6 |  |  |  |  |
|  | miR399f | 21 | AGUGUAGUUCUCCUUUGGCAG | 8 | 5 |  |  |  |  |
|  | miR399g | 21 | AGCCAAAGACGAUUUUGCCCU | 4 | 4 |  |  |  |  |
|  | miR399h | 21 | UCUGCCAAAGGAGAUUUGCUC | 4 | 3 |  |  |  |  |
|  | miR399i | 19 | CGCCAAGGAAAGCUGCUUU | 1 | 1 |  |  |  |  |
| miR403 | miR403a | 19 | UUAGAUUCACGCACAAACU | 1,970 | 1,538 | 1,979 | 1,542 | 0.37 | 0.49 |
|  | miR403b | 20 | UUUUUUCUGCUACUCUAAUU | 9 | 4 |  |  |  |  |
| miR408 | miR408a | 21 | ACAGAGACGAGACAGAGCAUG | 12,495 | 408 | 12,879 | 663 | 2.39 | 0.21 |
|  | miR408b | 21 | CUGGGAACUUGCUAGAGAUGA | 236 | 173 |  |  |  |  |
|  | miR408c | 21 | ACAGGGACAAGACAGAGCAUG | 75 | 50 |  |  |  |  |
|  | miR408d | 21 | CAGGUACGAGACAGAGCAUGG | 35 | 13 |  |  |  |  |
|  | miR408e | 21 | AUGCACUGCCUCUUCCCUGGC | 30 | 14 |  |  |  |  |
|  | miR408f | 24 | CUGCACUGCCUCUUCCCUGGCAUC | 8 | 5 |  |  |  |  |
| **Non-conserved (Group II)** | | | | | | | | | |
| miR1023 | miR1023a | 24 | AGGAACUUGAGAGCCAGAGUGCAU | 110 | 178 | 143 | 235 | 0.03 | 0.07 |
|  | miR1023b | 21 | AGAAGACUUGAAGACAUGCAU | 24 | 42 |  |  |  |  |
|  | miR1023c | 21 | AGGGAAUCGGAUAGUAAUGUA | 5 | 9 |  |  |  |  |
|  | miR1023d | 24 | AGGGACAUCAGAGUGAGAGUGCAU | 4 | 6 |  |  |  |  |
| miR1024 | miR1024 | 22 | UUCAUGGUUGGAUUGUAGACUC | 7 | 0 | 7 | 0 | 0.00 | 0.00 |
| miR1026 | miR1026 | 21 | UGAAAUGACUUGAGAGGAGUA | 6,994 | 5,515 | 6,994 | 5,515 | 1.30 | 1.75 |
| miR1027 | miR1027 | 19 | UUUUAUUUUCUAUUCCAUC | 0 | 73 | 0 | 73 | 0.00 | 0.02 |
| miR1028 | miR1028 | 22 | UGCAUUGUAGGUUUAAUAGAGG | 7 | 58 | 7 | 58 | 0.00 | 0.02 |
| miR1030 | miR1030a | 21 | UCUGCAUUUGCACCUGCACCU | 46 | 15 | 54 | 19 | 0.01 | 0.01 |
|  | miR1030b | 21 | CCUGCACCUGACAUUGCACCA | 8 | 4 |  |  |  |  |
| miR1034 | miR1034 | 19 | UGACUUUGCAGCGCUUACU | 8 | 0 | 8 | 0 | 0.00 | 0.00 |
| miR1037 | miR1037 | 21 | AGCCUUUUAGGAUUGGGAGGG | 0 | 4 | 0 | 4 | 0.00 | 0.00 |
| miR1038 | miR1038 | 20 | UAGGUGCUUUUCUACUCAAA | 0 | 3 | 0 | 3 | 0.00 | 0.00 |
| miR1039 | miR1039 | 21 | GUGCGAGACGGUCUCAAGGAU | 3,805 | 329 | 3,805 | 329 | 0.70 | 0.10 |
| miR1040 | miR1040 | 21 | CGAACACUAAAUGAACAUGUUCA | 6 | 62 | 6 | 62 | 0.00 | 0.02 |
| miR1042 | miR1042a | 21 | GGUGAUGCAGGAACUAACAGG | 272 | 1 | 304 | 25 | 0.06 | 0.01 |
|  | miR1042b | 24 | UGCUCUAUUCUCCCACGGCCCCGG | 32 | 24 |  |  |  |  |
| miR1044 | miR1044 | 20 | UAGUGUACAUAUUUGUUUUC | 52 | 30 | 52 | 30 | 0.01 | 0.01 |
| miR1046 | miR1046a | 18 | UGAUUUCAUAUUUACAAG | 84 | 90 | 107 | 118 | 0.02 | 0.04 |
|  | miR1046b | 21 | UAGGAUUUACAUAUUUUCACA | 23 | 28 |  |  | 0.00 | 0.00 |
| miR1048 | miR1048 | 21 | UGGAAAUGAGUGUAUGACAAC | 122 | 127 | 122 | 127 | 0.02 | 0.04 |
| miR1051 | miR1051 | 20 | GGUUAAGUGAAGAAGAAGUA | 75 | 0 | 75 | 0 | 0.01 | 0.00 |
| miR1052 | miR1052 | 21 | UUACUUUUAUGAUUGGUUGUA | 28 | 3 | 28 | 3 | 0.01 | 0.00 |
| miR1053 | miR1053 | 24 | UUCCUACUUCUGAACAUAGCCACU | 1 | 0 | 1 | 0 | 0.00 | 0.00 |
| miR1057 | miR1057 | 24 | UUUUGUAGGUGCUAGCUGGGCGGU | 12 | 0 | 12 | 0 | 0.00 | 0.00 |
| miR1058 | miR1058 | 24 | AGAAGAUUCCAUCCGAAGUACGAC | 11 | 0 | 11 | 0 | 0.00 | 0.00 |
| miR1060 | miR1060 | 18 | UUUGCCAAGGAUUACAAG | 43 | 0 | 43 | 0 | 0.01 | 0.00 |
| miR1061 | miR1061a | 19 | AGAGUAGUAUGGAAUUAUG | 101 | 165 | 111 | 180 | 0.02 | 0.06 |
|  | miR1061b | 18 | UUAUUUCAUAAUACUCGA | 10 | 15 |  |  |  |  |
| miR1063 | miR1063 | 21 | AUAUUGAGAAUACUGCAUCUU | 764 | 0 | 764 | 0 | 0.14 | 0.00 |
| miR1070 | miR1070 | 19 | UGGCUUCUAAAAAACUUGC | 0 | 106 | 0 | 106 | 0.00 | 0.03 |
| miR1072 | miR1072 | 23 | UGCAUAUGUGAGAUUUGAACUUA | 3 | 12 | 3 | 12 | 0.00 | 0.00 |
| miR1073 | miR1073 | 22 | AGGGACUGUUAAUACAUUUCAU | 1,903 | 28 | 1,903 | 28 | 0.35 | 0.01 |
| miR1074 | miR1074 | 21 | AGGGAUGUGAUGUUGUGUUGA | 54 | 1,100 | 54 | 1,100 | 0.01 | 0.35 |
| miR1075 | miR1075 | 19 | UGUGCAGUCAUGUUUUCUC | 7 | 0 | 7 | 0 | 0.00 | 0.00 |
| miR1077 | miR1077 | 19 | AGGGAAGGACGUAACAGAC | 94 | 0 | 94 | 0 | 0.02 | 0.00 |
| miR1078 | miR1078 | 24 | UUUGGAUGAUGUGAAUUUGUGUAU | 100 | 138 | 100 | 138 | 0.02 | 0.04 |
| miR1079 | miR1079 | 24 | AACCAAAAGAAUAGGUAUUGUAGU | 7 | 4 | 7 | 4 | 0.00 | 0.00 |
| miR1080 | miR1080 | 24 | UUCAUAUCUUCAAACACACCUCUU | 4 | 0 | 4 | 0 | 0.00 | 0.00 |
| miR1081 | miR1081 | 21 | AGAGGUCUUGGCUUUGAUUUC | 190 | 173 | 190 | 173 | 0.04 | 0.05 |
| miR1082 | miR1082 | 19 | GUGUUGGGCUGGGCCGGCA | 330 | 1 | 330 | 1 | 0.06 | 0.00 |
| miR1083 | miR1083 | 22 | AGCCUGGAUCGAAGCACGCCGU | 104 | 0 | 104 | 0 | 0.02 | 0.00 |
| miR1085 | miR1085 | 19 | UUGAGAGUUUGUAGGGACG | 3,439 | 4,631 | 3,439 | 4,631 | 0.64 | 1.47 |
| miR1087 | miR1087 | 20 | UACAGAUUUUGUAGUGCAUU | 11 | 180 | 11 | 180 | 0.00 | 0.06 |
| miR1088 | miR1088 | 24 | AGAAGAAAAGAGAGAACACGCGAU | 16 | 9 | 16 | 9 | 0.00 | 0.00 |
| miR1089 | miR1089 | 21 | UGAACAUCUAGAGAUUUUUGC | 10 | 6 | 10 | 6 | 0.00 | 0.00 |
| miR1091 | miR1091 | 24 | ACGGAGUGAGGGAUUGGUAUUUGC | 7 | 5 | 7 | 5 | 0.00 | 0.00 |
| miR1092 | miR1092 | 21 | AGAAGCGAAUCCAUUGGUGUU | 2 | 4 | 2 | 4 | 0.00 | 0.00 |
| miR1097 | miR1097 | 24 | GUAGCAUUGUUGUAUGUUGGAUGA | 20 | 45 | 20 | 45 | 0.00 | 0.01 |
| miR1098 | miR1098 | 23 | UUGAUGACUCUUGUUGCUGAAAU | 3 | 0 | 3 | 0 | 0.00 | 0.00 |
| miR1100 | miR1100 | 24 | UGAUCUACGGUAUAGAACCCACUC | 2 | 0 | 2 | 0 | 0.00 | 0.00 |
| miR1101 | miR1101 | 20 | AGGCAUUCUCUCGAACAGGU | 17 | 34 | 17 | 34 | 0.00 | 0.01 |
| miR1103 | miR1103 | 23 | UGGAAAAAUGUGGUCAUUGCUUG | 17 | 34 | 17 | 34 | 0.00 | 0.01 |
| miR1105 | miR1105 | 20 | UUUGGAUGUGGAAGACGCUC | 127 | 0 | 127 | 0 | 0.02 | 0.00 |
| miR1106 | miR1106 | 20 | UUUAGAAGGUGGAUGUGUGA | 184 | 0 | 184 | 0 | 0.03 | 0.00 |
| miR1108 | miR1108 | 21 | UGACCUAGAGACCAUAACCCC | 57 | 82 | 57 | 82 | 0.01 | 0.03 |
| miR1109 | miR1109 | 22 | UAGAGGGAGGAUUUUGUGCUAA | 57 | 3,243 | 57 | 3,243 | 0.01 | 1.03 |
| miR1110 | miR1110 | 24 | GUAGGUCGGACAGUGGUCAAGGAA | 4 | 3 | 4 | 3 | 0.00 | 0.00 |
| miR1111 | miR1111 | 21 | UCAUGACUACAACAGGACCAG | 4 | 13 | 4 | 13 | 0.00 | 0.00 |
| miR1112 | miR1112 | 23 | AGAGAAUGCUAUAGACUGAGUCA | 36 | 26 | 36 | 26 | 0.01 | 0.01 |
| miR1114 | miR1114 | 20 | AAGGGACAAAAAAGGAGCAG | 37 | 168 | 37 | 168 | 0.01 | 0.05 |
| miR1115 | miR1115 | 21 | UGAGCUCGGCACUUUGGGAAG | 49 | 10 | 49 | 10 | 0.01 | 0.00 |
| miR1118 | miR1118a | 24 | CACUGACUUAUGAUAUUGGAGGGA | 262 | 41 | 286 | 60 | 0.05 | 0.02 |
|  | miR1118b | 24 | AAGCUGACAUAUGGAAUUGAGGGA | 24 | 19 |  |  |  |  |
| miR1120 | miR1120 | 24 | ACAUGACUAUAUAUUUGGGACGGA | 232 | 346 | 232 | 346 | 0.04 | 0.11 |
| miR1121 | miR1121 | 21 | AGUAGUGAUCUGAAGCGCCUU | 9 | 0 | 9 | 0 | 0.00 | 0.00 |
| miR1122 | miR1122 | 22 | UAAAAACAUCCAUGUAUCUAGA | 50 | 0 | 50 | 0 | 0.01 | 0.00 |
| miR1123 | miR1123 | 23 | UAUCCGUGAGACGGUCUCAUAUA | 370 | 457 | 370 | 457 | 0.07 | 0.14 |
| miR1125 | miR1125 | 24 | AACAACUGAACCAACUAGCGGCUG | 0 | 120 | 0 | 120 | 0.00 | 0.04 |
| miR1126 | miR1126 | 24 | UCAAAUCUGGACUAAUACGGAGAG | 10 | 40 | 10 | 40 | 0.00 | 0.01 |
| miR1127 | miR1127 | 21 | AAUCCUUCCCGUUGGAAUUAC | 2 | 0 | 2 | 0 | 0.00 | 0.00 |
| miR1133 | miR1133 | 24 | CAAUACUCCCUCUGUCCUGUAAAG | 2 | 3 | 2 | 3 | 0.00 | 0.00 |
| miR1134 | miR1134 | 24 | ACAACAAGAACUAGAGAAGAAGAU | 517 | 531 | 517 | 531 | 0.10 | 0.17 |
| miR1137 | miR1137 | 20 | UUGUCACGAAGUUGAGUCAU | 159 | 0 | 159 | 0 | 0.03 | 0.00 |
| miR1138 | miR1138 | 24 | GCUUAGACGGACAUCCUCUAAAUA | 2 | 45 | 2 | 45 | 0.00 | 0.01 |
| miR1139 | miR1139 | 24 | AAAUACACAUACACUAAGUAACUA | 13 | 13 | 13 | 13 | 0.00 | 0.00 |
| miR1143 | miR1143 | 19 | AGAGUCGUCCCCUUAGUGA | 6 | 0 | 6 | 0 | 0.00 | 0.00 |
| miR1144 | miR1144 | 21 | UGGAACCGGGCAGCUCGGAUG | 2,315 | 192 | 2,315 | 192 | 0.43 | 0.06 |
| miR1146 | miR1146 | 20 | AAUGGGCCGAUCGGGUAUCU | 5 | 0 | 5 | 0 | 0.00 | 0.00 |
| miR1147 | miR1147 | 19 | AUAUCGGCCAAGUGGCAGA | 0 | 141 | 0 | 141 | 0.00 | 0.04 |
| miR1148 | miR1148 | 23 | UGGAGAUCGGUUCUGUUCCGGCU | 15 | 13 | 15 | 13 | 0.00 | 0.00 |
| miR1150 | miR1150 | 21 | UGGACGGCAGACUGGGGCCAA | 343 | 496 | 343 | 496 | 0.06 | 0.16 |
| miR1153 | miR1153 | 21 | AGAUUGUAUAUCAGAUGGCUC | 153 | 11 | 153 | 11 | 0.03 | 0.00 |
| miR1154 | miR1154 | 20 | AUUAGUCAUCGCCAAGGCUA | 4 | 44 | 4 | 44 | 0.00 | 0.01 |
| miR1156 | miR1156 | 22 | UUUUCACGGGAGCUUCAGGCAC | 88 | 38 | 88 | 38 | 0.02 | 0.01 |
| miR1157 | miR1157 | 24 | AGCCACGGACCCGCUAUUUGAAUC | 5 | 0 | 5 | 0 | 0.00 | 0.00 |
| miR1158 | miR1158 | 18 | ACUUGGAGAGGUCACUUG | 0 | 50 | 0 | 50 | 0.00 | 0.02 |
| miR1159 | miR1159 | 19 | AAAUGCGAAUGGAGAUGGA | 659 | 50 | 659 | 50 | 0.12 | 0.02 |
| miR1160 | miR1160 | 21 | CGAGAAGGAAGACAGACGGAU | 4,297 | 5 | 4,297 | 5 | 0.80 | 0.00 |
| miR1162 | miR1162 | 22 | CGGCUUAAUUUGACUCAACACG | 0 | 131 | 0 | 131 | 0.00 | 0.04 |
| miR1163 | miR1163 | 19 | GGGGCAUGUGCAUGCCAGG | 299 | 2 | 299 | 2 | 0.06 | 0.00 |
| miR1164 | miR1164 | 24 | UGGUGCAAAGGACCUGAGUGGUGU | 55 | 0 | 55 | 0 | 0.01 | 0.00 |
| miR1166 | miR1166 | 20 | AUAGGUCCAGACUUCAUGGG | 0 | 16 | 0 | 16 | 0.00 | 0.01 |
| miR1168 | miR1168 | 21 | AGUGCACAAGGCUCAAGUCCA | 11 | 0 | 11 | 0 | 0.00 | 0.00 |
| miR1169 | miR1169 | 23 | UGUGGAUGCUUGCUUUGAUGGCU | 22 | 0 | 22 | 0 | 0.00 | 0.00 |
| miR1171 | miR1171 | 24 | GGAGUGGAGUGUAGUGGGGUGGGA | 5 | 8 | 5 | 8 | 0.00 | 0.00 |
| miR1172 | miR1172 | 22 | AGGAUUGCACAGCAGAGGAGGC | 50 | 0 | 50 | 0 | 0.01 | 0.00 |
| miR1173 | miR1173 | 20 | AUGGUUGCAAGAGAAACAUU | 12 | 0 | 12 | 0 | 0.00 | 0.00 |
| miR1211 | miR1211 | 23 | UGAUGACGACGUACUCUUCCUGC | 3 | 0 | 3 | 0 | 0.00 | 0.00 |
| miR1217 | miR1217 | 21 | AAUUUGAAGAUGAUGAACAAG | 264 | 4 | 264 | 4 | 0.05 | 0.00 |
| miR1222 | miR1222a | 21 | GUUGAGAGUUCGAUUGGUAUA | 9 | 382 | 36 | 431 | 0.01 | 0.14 |
|  | miR1222b | 19 | CAAGGAGUUCAACGGUACA | 13 | 26 |  |  |  |  |
|  | miR1222c | 20 | UUUAGUAGAGUCAUACACUU | 8 | 14 |  |  |  |  |
|  | miR1222d | 21 | UUGUAGACGUCAUGUACUCGG | 6 | 9 |  |  |  |  |
| miR1223 | miR1223a | 20 | UUUAGUAGAGUCAUACACUU | 15 | 39 | 17 | 81 | 0.00 | 0.03 |
|  | miR1223b | 21 | UUGUAGACGUCAUGUACUCGG | 0 | 35 |  |  |  |  |
|  | miR1223c | 21 | UUGUAGACGUUGCACCUCAUG | 2 | 7 |  |  |  |  |
| miR1309 | miR1309 | 23 | UGUGAGCCCUUUUGAGAUGGACA | 0 | 104 | 0 | 104 | 0.00 | 0.03 |
| miR1310 | miR1310 | 20 | GGCAUCGGGGGCGCAACGCC | 531 | 185 | 531 | 185 | 0.10 | 0.06 |
| miR1312 | miR1312 | 22 | UUUGUAGAAAAAUGGCCGGCAU | 6,746 | 7,175 | 6,746 | 7,175 | 1.25 | 2.27 |
| miR1313 | miR1313 | 21 | UACACCUGAAUUAUUGUCUCG | 33 | 0 | 33 | 0 | 0.01 | 0.00 |
| miR1314 | miR1314 | 24 | UCGGCCUAUGAGAUGUAUGAGACA | 47 | 28 | 47 | 28 | 0.01 | 0.01 |
| miR1315 | miR1315 | 19 | UGGAGGCUGCACGUUGCCA | 0 | 17 | 0 | 17 | 0.00 | 0.01 |
| miR1316 | miR1316 | 24 | UCCAUACUACAACCAUUGGACUGA | 6 | 0 | 6 | 0 | 0.00 | 0.00 |
| miR1317 | miR1317 | 23 | GAAAUUAUAUUGGGACGUAUCUG | 2 | 0 | 2 | 0 | 0.00 | 0.00 |
| miR1318 | miR1318 | 18 | UCAGGAAGGAGACACCGA | 650 | 38 | 650 | 38 | 0.12 | 0.01 |
| miR1319 | miR1319 | 23 | AGAACCGGCUCUGAAUAUAUAUA | 3 | 17 | 3 | 17 | 0.00 | 0.01 |
| miR1320 | miR1320a | 21 | AGUAAAAUUUAUUCCGUUCAA | 5 | 5 | 7 | 8 | 0.00 | 0.00 |
|  | miR1320b | 19 | AGGAAGGAGGAUUUUAUGG | 2 | 3 |  |  |  |  |
| miR1424 | miR1424 | 24 | AUUGCACAGACUGAUGUUGAUUGU | 2 | 0 | 2 | 0 | 0.00 | 0.00 |
| miR1425 | miR1425 | 23 | UAGGAUUCAACCCUUACUGCUGA | 2 | 0 | 2 | 0 | 0.00 | 0.00 |
| miR1426 | miR1426 | 22 | AGAAUCUUGAUGAUGUUCUACA | 5 | 8 | 5 | 8 | 0.00 | 0.00 |
| miR1428 | miR1428a | 21 | AAGACAAUGGCCAUGAAUCUG | 229 | 15 | 277 | 45 | 0.05 | 0.01 |
|  | miR1428b | 22 | UAAGAAAAGCCAAUUGAAUUUG | 36 | 22 |  |  |  |  |
|  | miR1428c | 19 | UCAGAUAAUGACAUAAUUG | 12 | 8 |  |  |  |  |
| miR1429 | miR1429 | 19 | UAUAUACAAUCCGUGCAUC | 9 | 12 | 9 | 12 | 0.00 | 0.00 |
| miR1432 | miR1432 | 24 | GAGGUGUCAACUGCUCUGAAGGCA | 59 | 0 | 59 | 0 | 0.01 | 0.00 |
| miR1433 | miR1433 | 18 | UGGCGCUCCUCGGCUAGC | 53 | 41 | 53 | 41 | 0.01 | 0.01 |
| miR1435 | miR1435 | 18 | AUACUUAUCAAACUUUUU | 97 | 7 | 97 | 7 | 0.02 | 0.00 |
| miR1436 | miR1436 | 24 | ACUAUAUAUUUGGGACGGAGGGAG | 906 | 182 | 906 | 182 | 0.17 | 0.06 |
| miR1438 | miR1438 | 24 | AGGGAUACAUUUUAUAAUUUUGAA | 14 | 36 | 14 | 36 | 0.00 | 0.01 |
| miR1439 | miR1439 | 23 | UAAUUGGGACGGAGUGAGUAUUA | 117 | 269 | 117 | 269 | 0.02 | 0.09 |
| miR1441 | miR1441 | 19 | CGGAUGUAGGAAAAGGUUU | 0 | 76 | 0 | 76 | 0.00 | 0.02 |
| miR1442 | miR1442 | 20 | AAUUAUGUAACUAGAUGUGU | 94 | 93 | 94 | 93 | 0.02 | 0.03 |
| miR1444 | miR1444 | 20 | UCGCAAUUCGUCAAAUGUUC | 194 | 11 | 194 | 11 | 0.04 | 0.00 |
| miR1445 | miR1445 | 22 | UCCCUUGGUAGAGCAGAAAGAA | 537 | 635 | 537 | 635 | 0.10 | 0.20 |
| miR1446 | miR1446 | 21 | UUCCUGAACUGCUUCCCUGAA | 0 | 64 | 0 | 64 | 0.00 | 0.02 |
| miR1447 | miR1447 | 24 | CAGAAUUGCAGAAGACCUUGGAUU | 13 | 0 | 13 | 0 | 0.00 | 0.00 |
| miR1449 | miR1449 | 24 | AUGAGGGCACGAUCAAAAUAACUC | 7 | 126 | 7 | 126 | 0.00 | 0.04 |
| miR1507 | miR1507 | 24 | AGGUUGUGAUUGGAUACGAAAGAA | 25 | 7 | 25 | 7 | 0.00 | 0.00 |
| miR1508 | miR1508a | 21 | UAGACAGAGGGAAUAAAGUUG | 574 | 79 | 616 | 117 | 0.11 | 0.04 |
|  | miR1508b | 23 | AAGGAAAAGGAAAUAGCCAGUUG | 42 | 38 |  |  |  |  |
| miR1509 | miR1509a | 21 | UUGAAUUGUGAAAAUACGGUG | 48 | 49 | 70 | 78 | 0.01 | 0.02 |
|  | miR1509b | 24 | UAUCAAUCUGGAAAUAUACGGUGU | 12 | 13 |  |  |  |  |
|  | miR1509c | 21 | UUAGGAAGAGAAAUCACGGUU | 10 | 16 |  |  |  |  |
| miR1510 | miR1510a | 23 | AAGAGGAUUAGGUAUAAACAAAC | 20 | 2 | 36 | 3 | 0.01 | 0.00 |
|  | miR1510b | 21 | UGUGUUUUACUCUAUUUCCCC | 16 | 1 |  |  |  |  |
|  | miR1511 | 18 | AACCUGGCUCUGAUACCA | 3,423 | 3,206 | 3,423 | 3,206 | 0.63 | 1.01 |
|  | miR1512 | 22 | CAAUCUGAAAAUUCCAAAGUAU | 42 | 6 | 42 | 6 | 0.01 | 0.00 |
| miR1514 | miR1514a | 21 | UUCAUUUUUAAAUAUGCAAUA | 11 | 8 | 19 | 13 | 0.00 | 0.00 |
|  | miR1514b | 21 | UUCUUUUUAAAAUCAGACAGA | 8 | 5 |  |  |  |  |
| miR1515 | miR1515 | 22 | UCAUUUUUGCGUGCAGUGAUCC | 21 | 5 | 21 | 5 | 0.00 | 0.00 |
| miR1517 | miR1517 | 22 | AUCUGGUCAACUCGUUCGAAGA | 2 | 0 | 2 | 0 | 0.00 | 0.00 |
| miR1518 | miR1518 | 24 | UGUGUUGUAAAGAUGAAAUGACUC | 7 | 45 | 7 | 45 | 0.00 | 0.01 |
| miR1519 | miR1519 | 24 | UAGGAGUGUUACAAAAUUAGUCAU | 21 | 0 | 21 | 0 | 0.00 | 0.00 |
| miR1520 | miR1520a | 24 | AGGAGAUGAUACAUGCACAGUCCA | 500 | 970 | 1,924 | 2,791 | 0.36 | 0.88 |
|  | miR1520b | 24 | AUGUUGAUUAUUACGAUGACGGUA | 452 | 587 |  |  |  |  |
|  | miR1520c | 24 | AUCAGAUGAAUGACACGUGUACAA | 448 | 556 |  |  |  |  |
|  | miR1520d | 24 | AAUCAGAACAUGAACACGAAGAAA | 377 | 474 |  |  |  |  |
|  | miR1520f | 24 | AUCGGAAGAAUGACACGUGUACAA | 41 | 56 |  |  |  |  |
|  | miR1520e | 24 | GAAAUCAGAACAUGAACACGGAAA | 37 | 49 |  |  |  |  |
|  | miR1520f | 24 | UACACGAACAUGACACGAUGACAU | 22 | 30 |  |  |  |  |
|  | miR1520g | 24 | AAGGAAACGUGACAAUAACAACAA | 19 | 26 |  |  |  |  |
|  | miR1520h | 24 | UUCACAUCUGGUCUGGGCAUGAAA | 17 | 24 |  |  |  |  |
|  | miR1520i | 24 | GAUUGACCAACACGAACACGACAC | 11 | 19 |  |  |  |  |
| miR1522 | miR1522 | 19 | UUGUAUGGCUUAAUGAAAU | 677 | 95 | 677 | 95 | 0.13 | 0.03 |
| miR1523 | miR1523 | 19 | AGGGGAUAAAGUGAGACUA | 319 | 147 | 319 | 147 | 0.06 | 0.05 |
| miR1526 | miR1526 | 22 | CGGAAGAGGAUAAAUUAUACAA | 225 | 169 | 225 | 169 | 0.04 | 0.05 |
| miR1528 | miR1528 | 24 | AUUCAUUAGAUCGACAUAUAUUAU | 40 | 5 | 40 | 5 | 0.01 | 0.00 |
| miR1531 | miR1531 | 21 | UCUCAAUCUGGAAGACUUGUC | 0 | 37 | 0 | 37 | 0.00 | 0.01 |
| miR1533 | miR1533 | 18 | AUAAUAAAUAUAGAUAUA | 200 | 66 | 200 | 66 | 0.04 | 0.02 |
| miR1534 | miR1534 | 20 | UAAUUUUCGGUAAAUUGCAU | 65 | 14 | 65 | 14 | 0.01 | 0.00 |
| miR1535 | miR1535 | 19 | GUUGUUCUGUGGUGAGGUU | 31 | 34 | 31 | 34 | 0.01 | 0.01 |
| miR1536 | miR1536 | 23 | AAGUAGAGAGAAAUGUGUUUUAG | 37 | 0 | 37 | 0 | 0.01 | 0.00 |
| miR1846 | miR1846a | 22 | UAUCCGACGUACGCAGGGAGGC | 1 | 27 | 4 | 37 | 0.00 | 0.01 |
|  | miR1846b | 24 | AGGAGGAGGCUCGGGGCCGACUGA | 2 | 6 |  |  |  |  |
|  | miR1846c | 21 | GCAAUGAGGGGCCGGGACCAA | 1 | 4 |  |  |  |  |
| miR1850 | miR1850a | 21 | UGCAAAGGUUUGGAGAUUGGG | 56 | 11 | 68 | 19 | 0.01 | 0.01 |
|  | miR1850b | 22 | UUGAUGUGGAACUAAACGGUAG | 12 | 8 |  |  |  |  |
| miR1851 | miR1851 | 20 | GGGUCUGGGAUGGAUUUGGC | 0 | 80 | 0 | 80 | 0.00 | 0.03 |
| miR1852 | miR1852 | 24 | AUGAUGGAUUUCAAAUGACUAGGU | 710 | 1,060 | 710 | 1,060 | 0.13 | 0.34 |
| miR1854 | miR1854 | 24 | AUCUGGAUGAAAUUUGUAGAUGGA | 14 | 15 | 14 | 15 | 0.00 | 0.00 |
| miR1855 | miR1855 | 24 | AGCACUGUGAGAUACACCAAGAGA | 90 | 344 | 90 | 344 | 0.02 | 0.11 |
| miR1856 | miR1856 | 18 | UAUGCGAAGACGGACGAA | 88 | 331 | 88 | 331 | 0.02 | 0.10 |
| miR1857 | miR1857 | 21 | UGGUUUUUUUGAGCAGCGCGG | 46 | 74 | 46 | 74 | 0.01 | 0.02 |
| miR1858 | miR1858 | 20 | AGAGGAGGAGGAGGAGGGGC | 135 | 0 | 135 | 0 | 0.03 | 0.00 |
| miR1860 | miR1860 | 21 | AGAAAACAAGCAUUCCGAACU | 0 | 95 | 0 | 95 | 0.00 | 0.03 |
| miR1861 | miR1861a | 24 | CAAGUCUUGUGAGGAAGAAUGAGC | 62 | 72 | 62 | 72 | 0.01 | 0.02 |
|  | miR1861b | 22 | CGAACUUGAACAAGAACUGCAG | 27 | 90 | 27 | 90 | 0.01 | 0.03 |
|  | miR1861c | 22 | CGAGUGUUGUGGCAAGAAUGAC | 16 | 21 | 16 | 21 | 0.00 | 0.01 |
| miR1862 | miR1862 | 24 | AUAAGUUUGUAUUAUUUUUGGGAC | 5 | 28 | 5 | 28 | 0.00 | 0.01 |
| miR1863 | miR1863a | 23 | AGGCUCUGGAUACCAUGAAGAUU | 56 | 52 | 94 | 83 | 0.02 | 0.03 |
|  | miR1863b | 21 | GAGCUCUGAUACCAUAACUGU | 23 | 19 |  |  |  |  |
|  | miR1863c | 22 | AGAAACUUGGCUGACAUGCACU | 15 | 12 |  |  |  |  |
| miR1864 | miR1864 | 23 | UUGAGUAACGUGCAUGAUAAGGU | 0 | 1 | 0 | 1 | 0.00 | 0.00 |
| miR1865 | miR1865 | 21 | UCUCGUGAUGGUGUUUUCGAC | 0 | 1 | 0 | 1 | 0.00 | 0.00 |
| miR1866 | miR1866 | 24 | UUGAAAUUCUGAAAAACUUUCUUG | 8 | 4 | 8 | 4 | 0.00 | 0.00 |
| miR1867 | miR1867 | 23 | UGUUUUUUUGUAGAGAGAGGGGU | 0 | 23 | 0 | 23 | 0.00 | 0.01 |
| miR1868 | miR1868 | 24 | ACGGAAAAAGCAGGGACGCAGCCA | 10 | 7 | 10 | 7 | 0.00 | 0.00 |
| miR1869 | miR1869 | 23 | UGAGAACUAAUCGGAGGUAGGUA | 136 | 90 | 136 | 90 | 0.03 | 0.03 |
| miR1870 | miR1870 | 23 | AUUGGGCUAACUUCAGAUGACCA | 141 | 24 | 141 | 24 | 0.03 | 0.01 |
| miR1871 | miR1871 | 23 | AGGCUCUGAUACCAGUUGGGGUU | 757 | 6 | 757 | 6 | 0.14 | 0.00 |
| miR1872 | miR1872 | 23 | GAACUGUAGUAUGGACAGGUAUA | 68 | 7 | 68 | 7 | 0.01 | 0.00 |
| miR1873 | miR1873 | 22 | UCAACUGGUAUCAGAGCCUGAA | 60 | 29 | 60 | 29 | 0.01 | 0.01 |
| miR1874 | miR1874 | 22 | CAGGAUGAGGCUGUAACCCGUG | 32 | 26 | 32 | 26 | 0.01 | 0.01 |
| miR1875 | miR1875 | 23 | AACAAUGUAGAUGUGCAACAGAA | 725 | 37 | 725 | 37 | 0.13 | 0.01 |
| miR1877 | miR1877 | 24 | AGCAUGACAUGUGAAUGAUAGAUG | 148 | 178 | 148 | 178 | 0.03 | 0.06 |
| miR1878 | miR1878 | 24 | AACUUAACUAGGACACUUAGAAGA | 20 | 192 | 20 | 192 | 0.00 | 0.06 |
| miR1879 | miR1879 | 21 | GUGUUUGGUUUAGGGAUGGCG | 57 | 54 | 57 | 54 | 0.01 | 0.02 |
| miR1886 | miR1886 | 19 | UGAGGAAGGAGAUGAAGAC | 57 | 54 | 57 | 54 | 0.01 | 0.02 |
| miR1888 | miR1888 | 21 | UAAGUUAGAUUUAUGAAGGAU | 0 | 559 | 0 | 559 | 0.00 | 0.18 |
| miR1917 | miR1917 | 18 | UUAAUAAAAUUGUAAAGU | 451 | 6 | 451 | 6 | 0.08 | 0.00 |
| miR2079 | miR2079 | 19 | AGAGUAUGAUGUUAUGACG | 9 | 21 | 9 | 21 | 0.00 | 0.01 |
| miR2080 | miR2080 | 18 | UAUACAAUUUGCAGAUGC | 119 | 0 | 119 | 0 | 0.02 | 0.00 |
| miR2081 | miR2081 | 21 | AGCUAAAUUAUUUGUCUGAUU | 6 | 6 | 6 | 6 | 0.00 | 0.00 |
| miR2082 | miR2082 | 19 | GUGUGUCCGCUUUUUCGUU | 1 | 0 | 1 | 0 | 0.00 | 0.00 |
| miR2083 | miR2083 | 24 | AGAUUGACGACGUGUACAAGAUGA | 31 | 68 | 31 | 68 | 0.01 | 0.02 |
| miR2086 | miR2086 | 24 | GAACAUGACAUGACACGAACGGAA | 40 | 62 | 40 | 62 | 0.01 | 0.02 |
| miR2087 | miR2087 | 25 | AGAAGUAAUAGAACCGGCUGACUAC | 59 | 76 | 59 | 76 | 0.01 | 0.02 |
| miR2088 | miR2088 | 21 | UAGACCAGAUUACAUUGGACC | 368 | 692 | 368 | 692 | 0.07 | 0.22 |
| miR2089 | miR2089 | 24 | AGGAUUGGGAUGAAAUAGUAAAAU | 5 | 0 | 5 | 0 | 0.00 | 0.00 |
| miR2090 | miR2090 | 24 | AACUCUGAUCUAGAAGUCUUGUGU | 35 | 8 | 35 | 8 | 0.01 | 0.00 |
| miR2093 | miR2093 | 20 | GUGGCAUAAUCGGAAGAACA | 500 | 970 | 500 | 970 | 0.09 | 0.31 |
| miR2095 | miR2095 | 24 | CUGAAAUUUUACUAGAUGAACAUG | 0 | 23 | 0 | 23 | 0.00 | 0.01 |
| miR2097 | miR2097 | 24 | AGAGAACUGAGGACGGGAGAGAAG | 5 | 5 | 5 | 5 | 0.00 | 0.00 |
| miR2098 | miR2098 | 22 | CGGUCUGUCAAGUCGGAUGUGA | 3 | 0 | 3 | 0 | 0.00 | 0.00 |
| miR2099 | miR2099 | 24 | UGAAGAUGUUUGUAAAGCUUAUAA | 24 | 8 | 24 | 8 | 0.00 | 0.00 |
| miR2100 | miR2100 | 24 | AACUGGCUGAUUUGAGGCGGAGGG | 7 | 0 | 7 | 0 | 0.00 | 0.00 |
| miR2101 | miR2101 | 24 | AGUUUGUAACUCAAGUGGUAUUGU | 1,334 | 77 | 1,334 | 77 | 0.25 | 0.02 |
| miR2105 | miR2105 | 22 | UGUGUGUUGUGAAUGAUUGUAU | 55 | 6 | 55 | 6 | 0.01 | 0.00 |
| miR2108 | miR2108 | 24 | AUUAAUGUGUAUGUGUUUGGGUCG | 3 | 32 | 3 | 32 | 0.00 | 0.01 |
| miR2111 | miR2111 | 21 | UAAUCUGCAUCCUGAGGUUUA | 8 | 0 | 8 | 0 | 0.00 | 0.00 |
| miR2112 | miR2112 | 21 | CGAAAUCGAGAUAUCAAAUGU | 3 | 0 | 3 | 0 | 0.00 | 0.00 |
| miR2118 | miR2118a | 21 | UUCCAACUCCACCCAUUCCUA | 148 | 1 | 212 | 21 | 0.04 | 0.01 |
|  | miR2118b | 22 | UUCCUGAGCCUUCUGUUUCCUA | 49 | 12 |  |  |  |  |
|  | miR2118c | 24 | AUUCCUAAUGUUAUUCCAUUCCUA | 15 | 8 |  |  |  |  |
| miR2119 | miR2119 | 23 | UACAAUGGGAGAUGGUAGGGGAA | 51 | 0 | 627 | 0 | 0.12 | 0.00 |
| miR2120 | miR2120 | 23 | AAAGACUUUAGUGACGUGUUGUU | 552 | 0 |  |  |  |  |
| miR2123 | miR2123 | 24 | AAGAAGAUCAACGGUGUGCAACAC | 24 | 0 |  |  |  |  |
| miR2199 | miR2199 | 19 | UGAUAACUCGACGGAUCGC | 957 | 686 | 957 | 686 | 0.18 | 0.22 |
| miR2275 | miR2275a | 24 | AGAGUUGGAACGGGAAGCAAACUC | 66 | 97 | 123 | 203 | 0.02 | 0.06 |
|  | miR2275b | 23 | AGAAUUAUAAGGCAACUAGAACC | 23 | 49 |  |  |  |  |
|  | miR2275c | 21 | AGAUUAGACGGGACUUGGACA | 25 | 36 |  |  |  |  |
|  | miR2275d | 22 | AGAGAUUUGGAGGGAAAAAACU | 12 | 21 |  |  |  |  |
| miR2586 | miR2586 | 22 | CGAGGAAUGUCGUGCUUGCAUC | 3 | 39 | 3 | 39 | 0.00 | 0.01 |
| miR2590 | miR2590 | 24 | ACCUAAAGUGAUUGUAUUGUGCCA | 1 | 1 | 1 | 1 | 0.00 | 0.00 |
| miR2591 | miR2591 | 24 | GUGGACUACUACGGUACUACCUGC | 2 | 0 | 2 | 0 | 0.00 | 0.00 |
| miR2592 | miR2592 | 24 | AAAUGACUUGAGUGAUGUGUGCUU | 135 | 3,413 | 135 | 3,413 | 0.03 | 1.08 |
| miR2593 | miR2593 | 24 | UUAGAAUAGAAGAUGAACCUAAAU | 100 | 16 | 100 | 16 | 0.02 | 0.01 |
| miR2595 | miR2595 | 18 | CAUUUUCUUCUUUAUACU | 190 | 48 | 190 | 48 | 0.04 | 0.02 |
| miR2600 | miR2600 | 18 | AAUUGCAAUCACAAGGCC | 74 | 0 | 74 | 0 | 0.01 | 0.00 |
| miR2602 | miR2602 | 23 | UGGACAGUGAAUUGCCACCUCAG | 6 | 0 | 6 | 0 | 0.00 | 0.00 |
| miR2605 | miR2605 | 18 | ACUUAUUUAUAUGAAAUA | 59 | 0 | 59 | 0 | 0.01 | 0.00 |
| miR2607 | miR2607 | 18 | AUGUGAUUAUUGAUUGUG | 72 | 233 | 72 | 233 | 0.01 | 0.07 |
| miR2608 | miR2608 | 24 | AGUUGACAUAUAUCAUUACCUCAU | 23 | 0 | 23 | 0 | 0.00 | 0.00 |
| miR2610 | miR2610 | 24 | AAGAUGAGACUUGUAGAUGGCUUA | 2,704 | 1,566 | 2,704 | 1,566 | 0.50 | 0.50 |
| miR2611 | miR2611 | 19 | UAUUUGUCUGUUGGAUCAA | 12 | 117 | 12 | 117 | 0.00 | 0.04 |
| miR2616 | miR2616 | 24 | AGUUCGGUAUGGUUCGGGACGGAU | 0 | 436 | 0 | 436 | 0.00 | 0.14 |
| miR2619 | miR2619 | 24 | AACAUGAGGAGGCUUUUUGUAAUU | 140 | 0 | 140 | 0 | 0.03 | 0.00 |
| miR2620 | miR2620 | 21 | UCUGAAGACACCAGCUCUGAC | 0 | 87 | 0 | 87 | 0.00 | 0.03 |
| miR2621 | miR2621 | 19 | AGCUGGGCUAGAAAUUGUC | 36 | 0 | 36 | 0 | 0.01 | 0.00 |
| miR2624 | miR2624 | 21 | CGAAAGACAGUGGUUGCGGUU | 3 | 0 | 3 | 0 | 0.00 | 0.00 |
| miR2628 | miR2628 | 21 | CAUAAAAGAUGAACUGAGUAA | 186 | 131 | 186 | 131 | 0.03 | 0.04 |
| miR2630 | miR2630 | 18 | UGGUUUUGCCUUAUAUUU | 49 | 2 | 49 | 2 | 0.01 | 0.00 |
| miR2631 | miR2631 | 23 | UGACACGACACGAUAGCACGACU | 1,099 | 1,399 | 1,099 | 1,399 | 0.20 | 0.44 |
| miR2635 | miR2635 | 18 | AUAUUCUCACAUGACUAG | 315 | 0 | 315 | 0 | 0.06 | 0.00 |
| miR2637 | miR2637 | 24 | AAAUAUCUUCCUCAUGACUCCUGA | 0 | 155 | 0 | 155 | 0.00 | 0.05 |
| miR2638 | miR2638 | 18 | AUAUAAUAUGUGCAGUGG | 91 | 4 | 91 | 4 | 0.02 | 0.00 |
| miR2639 | miR2639 | 19 | UUGUCGGCUUACGUAAUUG | 17 | 0 | 17 | 0 | 0.00 | 0.00 |
| miR2641 | miR2641 | 18 | UUUUGAUCUUUUCGUUUA | 0 | 123 | 0 | 123 | 0.00 | 0.04 |
| miR2642 | miR2642 | 19 | AGAGUAUCUUCAAAUCAGU | 37 | 0 | 37 | 0 | 0.01 | 0.00 |
| miR2643 | miR2643 | 19 | UUUUGCUCAGAAAUUAAGA | 51 | 50 | 51 | 50 | 0.01 | 0.02 |
| miR2644 | miR2644 | 21 | CUAUUUCAGAUUGAUGGAGUU | 7 | 0 | 7 | 0 | 0.00 | 0.00 |
| miR2645 | miR2645 | 23 | UUUGUAGGAAGAUGAGUAUAUAU | 17 | 12 | 17 | 12 | 0.00 | 0.00 |
| miR2646 | miR2646 | 24 | AUGACAUGUAUACGUGAUGAUGUC | 101 | 149 | 101 | 149 | 0.02 | 0.05 |
| miR2648 | miR2648 | 24 | UGAGCCAAUGGGAGACAACAAGAU | 5 | 0 | 5 | 0 | 0.00 | 0.00 |
| miR2651 | miR2651 | 21 | UUUGAUUGGAUGGCUUGCAUU | 25 | 17 | 25 | 17 | 0.00 | 0.01 |
| miR2654 | miR2654 | 19 | AUUGAGGGACGAAAGUGUG | 0 | 132 | 0 | 132 | 0.00 | 0.04 |
| miR2655 | miR2655 | 23 | AGUUUAGGUCCUUUAACUUUUGA | 0 | 23 | 0 | 23 | 0.00 | 0.01 |
| miR2657 | miR2657 | 24 | UAGUUAUUUUAUCUAUUUUGUUGU | 0 | 1 | 0 | 1 | 0.00 | 0.00 |
| miR2658 | miR2658 | 20 | AUGGACAUUGUAUAUGAGAC | 126 | 28 | 126 | 28 | 0.02 | 0.01 |
| miR2660 | miR2660 | 21 | UAAGAUCAUCAGCAUAAACCA | 0 | 138 | 0 | 138 | 0.00 | 0.04 |
| miR2661 | miR2661 | 19 | UGGGUUUGAGAAAGUGGGC | 83 | 33 | 83 | 33 | 0.02 | 0.01 |
| miR2662 | miR2662 | 21 | GAGAAAAAUGUAAGACGGAAU | 6,008 | 0 | 6,008 | 0 | 1.11 | 0.00 |
| miR2663 | miR2663 | 20 | UUAAGAGGGCGUUUCAAAUU | 68 | 3 | 68 | 3 | 0.01 | 0.00 |
| miR2664 | miR2664 | 21 | AUUUGUGUUUGGGUUGAAGUC | 1 | 2 | 1 | 2 | 0.00 | 0.00 |
| miR2665 | miR2665 | 19 | UGUUUCAGGUAAGAAGUUG | 9 | 0 | 9 | 0 | 0.00 | 0.00 |
| miR2666 | miR2666 | 21 | UAAAGUCAGGAUUAUCAAGGA | 68 | 152 | 68 | 152 | 0.01 | 0.05 |
| miR2670 | miR2670 | 22 | CCAAGAAGUUGCUGCACUAGUU | 113 | 99 | 113 | 99 | 0.02 | 0.03 |
| miR2672 | miR2672 | 23 | UUAAGUCGAAACAAUGGGUACUA | 68 | 0 | 68 | 0 | 0.01 | 0.00 |
| miR2673 | miR2673 | 22 | CCUCUUCCUUCUUCAUUUUCCA | 9 | 0 | 9 | 0 | 0.00 | 0.00 |
| miR2675 | miR2675 | 21 | CGAGGCAUACAUUGAAGGAUU | 28 | 5 | 28 | 5 | 0.01 | 0.00 |
| miR2677 | miR2677 | 24 | UUUAUUGAAAUUGCUAUUAUGAAU | 2 | 0 | 2 | 0 | 0.00 | 0.00 |
| miR2678 | miR2678 | 21 | UGGAAAUUGUCGCAGUGUCUC | 4 | 93 | 4 | 93 | 0.00 | 0.03 |
| miR2862 | miR2862 | 19 | UAACGGCUUAGAUUUGUCC | 103 | 0 | 103 | 0 | 0.02 | 0.00 |
| miR2864 | miR2864 | 23 | CUGUUAUUGCAUCUGGAUAGGUA | 15 | 20 | 15 | 20 | 0.00 | 0.01 |
| miR2866 | miR2866 | 19 | AUCUAGUUUUGUCAACAUC | 7 | 0 | 7 | 0 | 0.00 | 0.00 |
| miR2868 | miR2868 | 18 | UUCUUUGUUUAGUAGAAA | 68 | 31 | 68 | 31 | 0.01 | 0.01 |
| miR2870 | miR2870 | 20 | AAUACAUUUCGGGAGACAAA | 0 | 137 | 0 | 137 | 0.00 | 0.04 |
| miR2873 | miR2873 | 23 | AAAUUGGACUAUACAUUUGGAAC | 222 | 133 | 222 | 133 | 0.04 | 0.04 |
| miR2875 | miR2875 | 23 | AUUUUCAGUCAUUACUAGUUAUA | 32 | 0 | 32 | 0 | 0.01 | 0.00 |
| miR2878 | miR2878 | 24 | AUACAUGUACAAAUUUCGAGGAUG | 28 | 3 | 28 | 3 | 0.01 | 0.00 |
| miR2879 | miR2879 | 24 | AGCGAGAUAGUUUAAAAUAAUGAC | 16 | 24 | 16 | 24 | 0.00 | 0.01 |
| miR2905 | miR2905 | 20 | UACUGUAGUGACACAGCGCA | 4 | 317 | 4 | 317 | 0.00 | 0.10 |
| miR2911 | miR2911 | 20 | GCCGGGGGACGGACUGGGAA | 1,133 | 823 | 1,133 | 823 | 0.21 | 0.26 |
| miR2912 | miR2912 | 20 | UCUAGAACUGAGGAUGGGAC | 231 | 272 | 231 | 272 | 0.04 | 0.09 |
| miR2916 | miR2916 | 22 | UGGGGGCUCGAAGACGAUCAGA | 671 | 334 | 671 | 334 | 0.12 | 0.11 |
| miR2920 | miR2920 | 22 | AAGAUCAACAAUUAAAUUUCAA | 174 | 5 | 174 | 5 | 0.03 | 0.00 |
| miR2922 | miR2922 | 20 | AAAUAAGUGAUGACGAAAUC | 0 | 32 | 0 | 32 | 0.00 | 0.01 |
| miR2923 | miR2923 | 24 | AACAAAAAUAUAAGUAUACAACAA | 36 | 62 | 36 | 62 | 0.01 | 0.02 |
| miR2925 | miR2925 | 22 | AUGGGCGGCCGCGUGGCUUGGU | 19 | 0 | 19 | 0 | 0.00 | 0.00 |
| miR2926 | miR2926 | 18 | AGGACUCGACGUUGGUGA | 24 | 10 | 24 | 10 | 0.00 | 0.00 |
| miR2927 | miR2927 | 23 | UGUGUCACGUCGACGGAGCCCUG | 422 | 3 | 422 | 3 | 0.08 | 0.00 |
| miR2928 | miR2928 | 18 | AAGGAGACAACAUUUUUU | 261 | 158 | 261 | 158 | 0.05 | 0.05 |
| miR2931 | miR2931 | 19 | AUUUAUUGUUCGAUGAAAA | 13 | 541 | 13 | 541 | 0.00 | 0.17 |
| miR2933 | miR2933 | 24 | AGAAAUCAGAGAGGACUAAUUCGC | 3 | 4 | 3 | 4 | 0.00 | 0.00 |
| miR2936 | miR2936 | 24 | CUGUGAGAAGAAGAGAACCAGAUG | 36 | 9 | 36 | 9 | 0.01 | 0.00 |
| miR2937 | miR2937 | 23 | AGAAGAAGCUGUUGAAGGAGGAC | 0 | 43 | 0 | 43 | 0.00 | 0.01 |
| miR2938 | miR2938 | 21 | AUCUUCUGAGAAGGGUUCGAG | 122 | 144 | 122 | 144 | 0.02 | 0.05 |
| miR2949 | miR2949 | 19 | UGAAAAUUGUCAAAAGUUA | 46 | 47 | 46 | 47 | 0.01 | 0.01 |
| miR2950 | miR2950a | 24 | GGUGUGCACGGGACUUGGAGAUCA | 216 | 165 | 477 | 355 | 0.09 | 0.11 |
|  | miR2950b | 23 | UGUGUGUGCAGGGUGUGAAUAUA | 261 | 190 |  |  |  |  |
| miR3434 | miR3434 | 21 | UUCGGAGUUAUCAGCCAUGGA | 101 | 127 | 101 | 127 | 0.02 | 0.04 |
| miR3434 | miR3434 | 18 | UCUAAAUCAGAGAAAACC | 23 | 29 | 23 | 29 | 0.00 | 0.01 |
| miR3435 | miR3435 | 21 | UGGACAUAAUUCUUCAAGUCA | 39 | 21 | 39 | 21 | 0.01 | 0.01 |
| miR3437 | miR3437 | 24 | AAAAAACAAGAUCGCCAUUCGGAU | 3 | 10 | 3 | 10 | 0.00 | 0.00 |
| miR3438 | miR3438 | 20 | UACAAGGAUUUAGCUAAAGC | 0 | 55 | 0 | 55 | 0.00 | 0.02 |
| miR3440 | miR3440 | 24 | UGGAUUGGUUCAAAUGAGACCGCA | 211 | 38 | 211 | 38 | 0.04 | 0.01 |
| miR3441 | miR3441 | 19 | UUCAUAGCCUCUUUGAGAA | 85 | 120 | 85 | 120 | 0.02 | 0.04 |
| miR3442 | miR3442 | 22 | UUCAGAGUUCUGACAUGGUGUA | 2,289 | 3,021 | 2,289 | 3,021 | 0.42 | 0.96 |
| miR3443 | miR3443 | 22 | UUGCCUUGGAGAUGGUAGAGUU | 0 | 19 | 0 | 19 | 0.00 | 0.01 |
| miR3444 | miR3444 | 24 | UUGUAGGACUCGACUGAAGAUCGA | 78 | 73 | 78 | 73 | 0.01 | 0.02 |
| miR3445 | miR3445 | 20 | UUUGGAGGUGAGUUGUUUGC | 1 | 2,128 | 1 | 2,128 | 0.00 | 0.67 |
| miR3446 | miR3446 | 22 | AUGAAGCUAGCACUGUGGCAGG | 0 | 208 | 0 | 208 | 0.00 | 0.07 |
| miR3447 | miR3447 | 18 | CGUGACGGACAAAUAAAG | 438 | 5 | 438 | 5 | 0.08 | 0.00 |
| miR3448 | miR3448 | 24 | UUGAGGAUUUUUUGUGUGGUGGCU | 134 | 105 | 134 | 105 | 0.02 | 0.03 |
| miR3449 | miR3449 | 23 | AGAUAGUAAACGAAUACUGGAUA | 191 | 98 | 191 | 98 | 0.04 | 0.03 |
| miR3451 | miR3451 | 21 | CGCGAUCCGGGAGGUUCACAA | 285 | 0 | 285 | 0 | 0.05 | 0.00 |
| miR3453 | miR3453 | 24 | GGCGGAAGAUGAGGAGAAGUGGUG | 7 | 0 | 7 | 0 | 0.00 | 0.00 |
| miR3454 | miR3454 | 19 | UUAAUCGGAGCUAGGCAGA | 1,536 | 1,235 | 1,536 | 1,235 | 0.28 | 0.39 |
| miR3454 | miR3454 | 18 | CUCAUCGGAGCUAGGCAG | 1,023 | 957 | 1,023 | 957 | 0.19 | 0.30 |
| miR3458 | miR3458 | 24 | GGGAGCUUUCUGAUUAGACGCAUA | 7 | 10 | 7 | 10 | 0.00 | 0.00 |
| miR3460 | miR3460 | 23 | GGGAACCUGCAGCUGGAUAGAGG | 1,437 | 1,471 | 1,437 | 1,471 | 0.27 | 0.47 |
| miR3461 | miR3461 | 19 | UCUGACUCGCUGAGCAGAA | 1,171 | 0 | 1,171 | 0 | 0.22 | 0.00 |
| miR3462 | miR3462 | 20 | GUUCCGGUUCUGGACGAGGC | 697 | 606 | 697 | 606 | 0.13 | 0.19 |
| miR3463 | miR3463 | 20 | AGCAGCGGAGCUGGAAGGGG | 101 | 52 | 101 | 52 | 0.02 | 0.02 |
| miR3464 | miR3464 | 20 | UGGGUAUGGGUUGGAGAUGG | 686 | 44 | 686 | 44 | 0.13 | 0.01 |
| miR3465 | miR3465 | 19 | CAACUGAAGACUAGAAGGA | 11,671 | 77 | 11,671 | 77 | 2.16 | 0.02 |
| miR3466 | miR3466 | 21 | UUCCGGAAGACACCACCACAA | 17 | 21 | 17 | 21 | 0.00 | 0.01 |
| miR3467 | miR3467 | 19 | UGCGGCUGAGAAGACCAGA | 231 | 0 | 231 | 0 | 0.04 | 0.00 |
| miR3468 | miR3468 | 22 | AGAGCGAAAAGGACGAGGAAGA | 26 | 22 | 26 | 22 | 0.00 | 0.01 |
| miR3509 | miR3509 | 24 | AUACUUGAAUCCAGACUUAGAUGA | 0 | 20 | 0 | 20 | 0.00 | 0.01 |
| miR3512 | miR3512 | 21 | UGGCAAAUGAUGACAAAAUAG | 16 | 103 | 16 | 103 | 0.00 | 0.03 |
| miR3513 | miR3513 | 23 | UAAAUUUCUGAGCUUGAUGCAUC | 15 | 111 | 15 | 111 | 0.00 | 0.04 |
| miR3514 | miR3514 | 24 | AGGUAUUCUGUAAGUAACUGGUGA | 17 | 35 | 17 | 35 | 0.00 | 0.01 |
| miR3515 | miR3515 | 20 | AAUUGUAGAAAAUAAUGGUA | 20 | 41 | 20 | 41 | 0.00 | 0.01 |
| miR3519 | miR3519 | 21 | CAAUUCUAUGACUGCAUUUCA | 519 | 785 | 519 | 785 | 0.10 | 0.25 |
| miR3520 | miR3520 | 24 | AGGGAUGGUGAAUAUGCUUCUCAU | 46 | 5 | 46 | 5 | 0.01 | 0.00 |
| miR3521 | miR3521 | 20 | UGGUGACUUGUAUACAUAUG | 27 | 9 | 27 | 9 | 0.01 | 0.00 |
| miR3522 | miR3522 | 24 | UGAGACCAAUGAACCAGAACUGAC | 44 | 31 | 44 | 31 | 0.01 | 0.01 |
| miR3522 | miR3522 | 21 | UGAGACGGAAAAGACAGCUGA | 36 | 25 | 36 | 25 | 0.01 | 0.01 |
| miR3623 | miR3623 | 24 | UGGAUGGCUUCGGAGAAUUUGCAA | 1,148 | 3 | 1,148 | 3 | 0.21 | 0.00 |
| miR3624 | miR3624 | 19 | AUUAGCUGCUGUGUUUAGA | 55 | 36 | 55 | 36 | 0.01 | 0.01 |
| miR3624 | miR3624 | 19 | UCAGCGGCAGCAAUAUACU | 41 | 27 | 41 | 27 | 0.01 | 0.01 |
| miR3625 | miR3625 | 23 | CGGGAGAUGAUGAUACUGUAAGC | 247 | 98 | 247 | 98 | 0.05 | 0.03 |
| miR3627 | miR3627 | 22 | UCUGUCCAGGAGAGCACGGCCU | 0 | 24 | 0 | 24 | 0.00 | 0.01 |
| miR3629 | miR3629 | 18 | CCAUUUUCUCGAGCCAAC | 187 | 481 | 187 | 481 | 0.03 | 0.15 |
| miR3629 | miR3629 | 24 | AGGCUGCUGGAGAUGAAAUGUAGA | 0 | 19 | 0 | 19 | 0.00 | 0.01 |
| miR3630 | miR3630 | 20 | UGGGAAUCUCUCUGAUGCUA | 9 | 9 | 9 | 9 | 0.00 | 0.00 |
| miR3631 | miR3631 | 22 | CUUGGAUGAUGUCUACAUAAGU | 266 | 458 | 266 | 458 | 0.05 | 0.14 |
| miR3633 | miR3633 | 18 | AGAAUGAUGGUUAGAAGG | 266 | 23 | 266 | 23 | 0.05 | 0.01 |
| miR3634 | miR3634 | 24 | AGGCAUAUGUUGACGCGAAUCAGA | 2 | 5 | 2 | 5 | 0.00 | 0.00 |
| miR3636 | miR3636 | 21 | UCUGUCGUGAGAAGAAUCGAG | 137 | 111 | 137 | 111 | 0.03 | 0.04 |
| miR3637 | miR3637 | 24 | AUUUUUCGUAUUGUGUUUUGUCAG | 3 | 93 | 3 | 93 | 0.00 | 0.03 |
| miR3638 | miR3638 | 24 | ACAACAAGACAUGAUAAGGACACC | 0 | 28 | 0 | 28 | 0.00 | 0.01 |
| miR3639 | miR3639 | 24 | AUUGACUUUGAUAGGCUUAAAUAG | 112 | 203 | 112 | 203 | 0.02 | 0.06 |
| miR3640 | miR3640 | 22 | CACAUGAUUGGUAUGCUUUUUU | 77 | 0 | 77 | 0 | 0.01 | 0.00 |
| miR3693 | miR3693 | 23 | UAGGGUUGCUCAUGAACUAGCUC | 43 | 0 | 43 | 0 | 0.01 | 0.00 |
| miR3695 | miR3695 | 24 | UUUGUCAGGCCUGGUCGUUUGGGU | 1 | 0 | 1 | 0 | 0.00 | 0.00 |
| miR3697 | miR3697 | 24 | UAGCGACCCUGACUUUAACGAUGG | 2 | 0 | 2 | 0 | 0.00 | 0.00 |
| miR3699 | miR3699 | 24 | GACAGAAGAAGACUCAUUGGAUCA | 472 | 359 | 472 | 359 | 0.09 | 0.11 |
| miR3700 | miR3700 | 23 | GAUCGCACACAACUGAAGGUACA | 9 | 0 | 9 | 0 | 0.00 | 0.00 |
| miR3702 | miR3702 | 20 | AAUCGUCUUGUGCUUAUUGG | 5 | 0 | 5 | 0 | 0.00 | 0.00 |
| miR3704 | miR3704 | 24 | AGGAUCUAGGCGGGUUGGAAUAAA | 61 | 0 | 61 | 0 | 0.01 | 0.00 |
| miR3705 | miR3705 | 18 | GUCAGUGUUUGUCUGGAC | 96 | 0 | 96 | 0 | 0.02 | 0.00 |
| miR3706 | miR3706 | 18 | UAUAGAGAAAUGGUAAGA | 0 | 574 | 0 | 574 | 0.00 | 0.18 |
| miR3707 | miR3707 | 21 | AGUGUUCUGCCAAAUCCUUGA | 3 | 0 | 3 | 0 | 0.00 | 0.00 |
| miR3710 | miR3710 | 23 | UGGGACAACCUCAGGGGCCUCCA | 10 | 0 | 10 | 0 | 0.00 | 0.00 |
| miR3711 | miR3711 | 22 | CGGCGCUAGAAGGAGGAUCCAU | 8 | 65 | 8 | 65 | 0.00 | 0.02 |
| miR3712 | miR3712 | 21 | UCUGAUCAAGAUUCAGACUAC | 15 | 19 | 15 | 19 | 0.00 | 0.01 |
| miR3932 | miR3932 | 21 | AACUUUGUGACUGAAAAAGAG | 496 | 44 | 496 | 44 | 0.09 | 0.01 |
| miR3933 | miR3933 | 24 | AGAGAGACAAAAGUGACGACUCAG | 36 | 6 | 36 | 6 | 0.01 | 0.00 |
| miR3946 | miR3946 | 22 | UGAGAAAAAGAGAAGAAGAGCA | 79 | 14 | 79 | 14 | 0.01 | 0.00 |
| miR3948 | miR3948 | 21 | UGGAUGUGGGAGGGGUAGGUG | 62 | 5 | 62 | 5 | 0.01 | 0.00 |
| miR3949 | miR3949 | 22 | UGAUGAUGAGGCAGAAAAUGAG | 37 | 1,200 | 37 | 1,200 | 0.01 | 0.38 |
| miR3950 | miR3950 | 19 | UUUUUCCAUACUGAUUUCU | 150 | 88 | 150 | 88 | 0.03 | 0.03 |
| miR3951 | miR3951 | 24 | UAGAUAAUCAGAUAGAGAAAACAG | 166 | 1,835 | 166 | 1,835 | 0.03 | 0.58 |
| miR3954 | miR3954 | 18 | UGGACAAACUCACGGUCA | 190 | 18 | 190 | 18 | 0.04 | 0.01 |
| miR400 | miR400 | 24 | GACUGGUAUAAUAAUUCAUUGAAG | 15 | 4 | 15 | 4 | 0.00 | 0.00 |
| miR402 | miR402a | 22 | UUCAGGCCUAUCAAAUCUCUGC | 4 | 3 | 6 | 4 | 0.00 | 0.00 |
|  | miR402b | 19 | ACGGAACCGUAGACCACGG | 2 | 1 |  |  |  |  |
| miR407 | miR407 | 22 | UUUAUUAUGCAUAUCUUUUGGU | 9 | 100 | 9 | 100 | 0.00 | 0.03 |
| miR413 | miR413 | 18 | AUAGUUUCUUUUGUUUAC | 60 | 16 | 60 | 16 | 0.01 | 0.01 |
| miR414 | miR414 | 20 | UCAUCAUCAUCAUCAUCUGC | 29 | 0 | 29 | 0 | 0.01 | 0.00 |
| miR415 | miR415a | 21 | AACUGAGCAGCAACCAGAACA | 1,618 | 790 | 1,674 | 822 | 0.31 | 0.26 |
|  | miR415b | 21 | AAUAGAAUCAGAAGCUGAGAG | 56 | 32 |  |  |  |  |
| miR417 | miR417 | 24 | AGAAGGUGACUGAGAUUUGUUCGA | 147 | 136 | 147 | 136 | 0.03 | 0.04 |
| miR418 | miR418 | 23 | UAAUGAGAUGAUGAUACGUGACA | 30 | 40 | 30 | 40 | 0.01 | 0.01 |
| miR419 | miR419a | 18 | UGAUGAAGACGACGAUGA | 2,091 | 274 | 2,136 | 311 | 0.40 | 0.10 |
|  | miR419b | 19 | UGGAAUGAUGAGAUGUUG | 45 | 37 |  |  |  |  |
| miR420 | miR420 | 19 | AAACUAGAUCACGGAAGCA | 38 | 12 | 38 | 12 | 0.01 | 0.00 |
| miR4221 | miR4221 | 22 | UUUUCAUUCUGUUGUAUUUUGC | 23 | 41 | 23 | 41 | 0.00 | 0.01 |
| miR4222 | miR4222 | 24 | UUUCAUUCAAUUUGUUGGACCAGC | 2 | 9 | 2 | 9 | 0.00 | 0.00 |
| miR4223 | miR4223 | 24 | ACUGGAAUUUGAUUAGAAGAAACA | 139 | 15 | 139 | 15 | 0.03 | 0.00 |
| miR4224 | miR4224 | 19 | CAACUGACUUCUCAACUUU | 8 | 0 | 8 | 0 | 0.00 | 0.00 |
| miR4225 | miR4225 | 24 | AAUGCGAUGGUUAAAACGAACGAC | 9 | 22 | 9 | 22 | 0.00 | 0.01 |
| miR4226 | miR4226 | 24 | AUACAAGAUGAUGCAUGAGCAAUA | 15 | 238 | 15 | 238 | 0.00 | 0.08 |
| miR4228 | miR4228 | 23 | AUAGCUUGUGAACGCGGUCGGUU | 5 | 7 | 5 | 7 | 0.00 | 0.00 |
| miR4231 | miR4231 | 19 | UAAAUCUUUUGUGCCAUCG | 52 | 59 | 52 | 59 | 0.01 | 0.02 |
| miR4232 | miR4232 | 24 | UACUCUUUAUUAGGAUUGUGGUGC | 2 | 0 | 2 | 0 | 0.00 | 0.00 |
| miR4233 | miR4233 | 24 | CAUCAUCAUCAUCAUCAACUGCAC | 19 | 40 | 19 | 40 | 0.00 | 0.01 |
| miR4234 | miR4234 | 24 | AAAUUUAUAAAUCAGUUGGCAAGU | 7 | 0 | 7 | 0 | 0.00 | 0.00 |
| miR4235 | miR4235 | 21 | ACGACAGCAGCUACAACCCCU | 41 | 114 | 41 | 114 | 0.01 | 0.04 |
| miR4237 | miR4237 | 24 | AAUUACGUAAACAUAUCAUAAUCA | 1 | 4 | 1 | 4 | 0.00 | 0.00 |
| miR4238 | miR4238 | 24 | UGUUUGCAAACUCUAAAUCCCCAA | 7 | 0 | 7 | 0 | 0.00 | 0.00 |
| miR4243 | miR4243 | 19 | UGAAAUUGUGUAUUUGUAC | 0 | 75 | 0 | 75 | 0.00 | 0.02 |
| miR4244 | miR4244 | 21 | UUGUGGAUCCAUGGAGAUUGA | 2 | 4 | 2 | 4 | 0.00 | 0.00 |
| miR4245 | miR4245 | 21 | ACAACCUUUUAUCUGACAAAU | 156 | 0 | 156 | 0 | 0.03 | 0.00 |
| miR4246 | miR4246 | 24 | AAAACACAAUUUUCGAUUGCGUAA | 4 | 8 | 4 | 8 | 0.00 | 0.00 |
| miR4248 | miR4248 | 22 | AAAUUUUAUUUUUGGACAAAUC | 0 | 95 | 0 | 95 | 0.00 | 0.03 |
| miR4249 | miR4249 | 24 | UGGAAUUUGAAACUAGUUGAGGUA | 20 | 27 | 20 | 27 | 0.00 | 0.01 |
| miR4250 | miR4250 | 18 | UCCAAAGGCAAGAACAAA | 1,081 | 31 | 1,081 | 31 | 0.20 | 0.01 |
| miR426 | miR426 | 19 | UUUUGGAAAUUUUCUUCAG | 8 | 0 | 8 | 0 | 0.00 | 0.00 |
| miR4341 | miR4341 | 24 | GGUGGAAAGUUUAACAAUGACGGA | 15 | 0 | 15 | 0 | 0.00 | 0.00 |
| miR4342 | miR4342 | 23 | AAGGUGAUUAGAAUUAGGAUGGU | 5 | 20 | 5 | 20 | 0.00 | 0.01 |
| miR4343 | miR4343 | 23 | AGAAACGUAGGAUCAAUGUUGAU | 82 | 100 | 82 | 100 | 0.02 | 0.03 |
| miR4344 | miR4344 | 24 | AAGUAGAAUUGCAGAAGACUUGCU | 111 | 209 | 111 | 209 | 0.02 | 0.07 |
| miR4345 | miR4345 | 24 | AAAAGACGGAACUAUAAAAAGAUU | 43 | 70 | 43 | 70 | 0.01 | 0.02 |
| miR4346 | miR4346 | 24 | GAAAGACCAAGAAGAGACAGCUGU | 843 | 1,274 | 843 | 1,274 | 0.16 | 0.40 |
| miR4347 | miR4347 | 23 | AAGACUGUCUUACGGAUCAGGAU | 43 | 0 | 43 | 0 | 0.01 | 0.00 |
| miR4348 | miR4348 | 24 | AAAACUGUGUAAGAUGGUCUCAUU | 30 | 13 | 30 | 13 | 0.01 | 0.00 |
| miR4349 | miR4349 | 22 | UAUUGGUGUAGAGAUAGAAAGA | 6 | 4 | 6 | 4 | 0.00 | 0.00 |
| miR4350 | miR4350 | 23 | UGCAAAGGCACAAGAGACAGACA | 0 | 103 | 0 | 103 | 0.00 | 0.03 |
| miR4351 | miR4351 | 22 | UUUGGGAUUCAGUCUGGAGAUG | 3,453 | 0 | 3,453 | 0 | 0.64 | 0.00 |
| miR4352 | miR4352 | 24 | UAUAAAUUAGGAUUCGUAAGACGG | 33 | 22 | 33 | 22 | 0.01 | 0.01 |
| miR4358 | miR4358 | 24 | AGUGCAUGAACUAUAUUGGCCUAG | 0 | 25 | 0 | 25 | 0.00 | 0.01 |
| miR4359 | miR4359 | 24 | ACACUAGUGACUCUAACAACGGUU | 63 | 123 | 63 | 123 | 0.01 | 0.04 |
| miR4360 | miR4360 | 24 | CAGUUGCACGGUGCGACGGAUUGC | 101 | 3 | 101 | 3 | 0.02 | 0.00 |
| miR4364 | miR4364 | 24 | CCGGAUGGACACGCGAAGAAGGUU | 2 | 0 | 2 | 0 | 0.00 | 0.00 |
| miR4365 | miR4365 | 24 | AGUAGGAUUCUUCGCGAGAUCGCA | 0 | 8 | 0 | 8 | 0.00 | 0.00 |
| miR4366 | miR4366 | 24 | CAUAUUAUGUAGUAGAUUUGAUGG | 1 | 139 | 1 | 139 | 0.00 | 0.04 |
| miR4368 | miR4368 | 24 | AAAGACGGUAAUUACUAGUAAUCA | 183 | 318 | 183 | 318 | 0.03 | 0.10 |
| miR4369 | miR4369 | 23 | GGUCAAGCUGAUCAAGGAACGGA | 0 | 18 | 0 | 18 | 0.00 | 0.01 |
| miR437 | miR437a | 19 | AAACAUAUAGAAGUCGAUU | 345 | 315 | 372 | 334 | 0.07 | 0.11 |
|  | miR437b | 19 | AAAGUAGCGAAGUUUCCUU | 15 | 12 |  |  |  |  |
|  | miR437c | 21 | AAAAGCUAGAGAAGUUUGCUG | 8 | 6 |  |  |  |  |
|  | miR437d | 22 | GAACUUAAAGAACGCUUGACUU | 4 | 1 |  |  |  |  |
| miR4370 | miR4370 | 24 | AGUAGCUCGGUACGAUUUUCGCUA | 8 | 16 | 8 | 16 | 0.00 | 0.01 |
| miR4371 | miR4371a | 22 | GAUUUGAAGACGGAAUAUCCAU | 167 | 18 | 218 | 27 | 0.04 | 0.01 |
|  | miR4371b | 23 | AAUGAUGCAGAUGACAAGGAAGG | 43 | 6 |  |  |  |  |
|  | miR4371c | 25 | AGUGAGAGCGAUGAGUAGACGGAGU | 8 | 3 |  |  |  |  |
| miR4372 | miR4372 | 23 | AAAAAUCGGGACGUGACGAGUCA | 8 | 5 | 8 | 5 | 0.00 | 0.00 |
| miR4373 | miR4373 | 24 | AAUUUGAGGUACGGUACGAUUGAC | 67 | 66 | 67 | 66 | 0.01 | 0.02 |
| miR4374 | miR4374 | 24 | UAAGACGGUCGCAUGAUUCAACGA | 0 | 27 | 0 | 27 | 0.00 | 0.01 |
| miR4376 | miR4376 | 22 | ACGCAGGAGAGAUGAUGCCCGU | 4,060 | 3,062 | 4,060 | 3,062 | 0.75 | 0.97 |
| miR4378 | miR4378 | 24 | AUAGGACUUUACUUGAUAUGGUGA | 19 | 27 | 19 | 27 | 0.00 | 0.01 |
| miR4379 | miR4379 | 22 | UAGAUGUGACUGGUGAGAGGCC | 85 | 16 | 85 | 16 | 0.02 | 0.01 |
| miR4380 | miR4380a | 21 | CGGAUGUUGAUGAUGCUGCAU | 10 | 7 | 15 | 10 | 0.00 | 0.00 |
|  | miR4380b | 24 | UCUGGUUAUACGGAUUAGUUAGAU | 5 | 3 |  |  |  |  |
| miR4381 | miR4381 | 23 | UAUGUGACAGAAACGAGAUCAAG | 767 | 558 | 767 | 558 | 0.14 | 0.18 |
| miR4382 | miR4382 | 24 | UAUUUACACAUAGAUUUCAUGCAU | 7 | 24 | 7 | 24 | 0.00 | 0.01 |
| miR4383 | miR4383 | 23 | UUGGACCAGCAGUUGAACCGGUC | 4 | 0 | 4 | 0 | 0.00 | 0.00 |
| miR4384 | miR4384 | 24 | AAUCAGACACAUGCAUCGAGACGA | 13 | 82 | 13 | 82 | 0.00 | 0.03 |
| miR4385 | miR4385 | 22 | AAUACGUUGUAGAAAUGAUGGU | 644 | 1,072 | 644 | 1,072 | 0.12 | 0.34 |
| miR4386 | miR4386 | 23 | UGAAGAGUUCUGGAAGAUCUGCA | 0 | 219 | 0 | 219 | 0.00 | 0.07 |
| miR4387 | miR4387a | 24 | AAUCCAAGACCUGAUGACGUGACA | 17 | 25 | 23 | 34 | 0.00 | 0.01 |
|  | miR4387b | 22 | AAGGUGAUGGCAUCACAUCUGG | 5 | 6 |  |  |  |  |
|  | miR4387c | 24 | AAUGUGAUGAUUAGGAUGAUGAGU | 1 | 3 |  |  |  |  |
| miR4388 | miR4388 | 24 | AGAACUUAUGGGACCAAAUUGCAC | 42 | 16 | 42 | 16 | 0.01 | 0.01 |
| miR4390 | miR4390 | 23 | UCGGUCCUGUCGGGUUUCGGGUU | 8 | 1 | 8 | 1 | 0.00 | 0.00 |
| miR4391 | miR4391 | 24 | CUGGCAAAGAACCAAGAGGAGAAG | 67 | 159 | 67 | 159 | 0.01 | 0.05 |
| miR4392 | miR4392 | 22 | UACUGGAAAAUUGAUUUCGGUA | 3,669 | 3,220 | 3,669 | 3,220 | 0.68 | 1.02 |
| miR4393 | miR4393a | 24 | UGACGAAAAGGAGCGGAAAACC | 14 | 54 | 19 | 62 | 0.00 | 0.02 |
|  | miR4393b | 22 | UUGAAAAGGGCACAAGAAGACU | 5 | 8 |  |  |  |  |
| miR4395 | miR4395 | 22 | UGGAUUCGAGAUGGGCUUGAUG | 9 | 0 | 9 | 0 | 0.00 | 0.00 |
| miR4396 | miR4396 | 24 | UGAGAUUAUAAGACGAUGCGUGAC | 191 | 232 | 191 | 232 | 0.04 | 0.07 |
| miR4397 | miR4397 | 18 | UGCAAGAUGUGGCGAAUU | 0 | 414 | 0 | 414 | 0.00 | 0.13 |
| miR4398 | miR4398 | 23 | UGCAGUGGAAUAGAAGACGAAAC | 1,966 | 2,239 | 1,966 | 2,239 | 0.36 | 0.71 |
| miR4400 | miR4400 | 21 | AUUCGGAAAAUUGGAAGACGC | 971 | 879 | 971 | 879 | 0.18 | 0.28 |
| miR4403 | miR4403 | 24 | ACGACACGAUAGCACGACACGAAC | 2,778 | 3,443 | 2,778 | 3,443 | 0.51 | 1.09 |
| miR4404 | miR4404 | 23 | GAUUCUGGAAGACUGAAGGAUAA | 104 | 0 | 104 | 0 | 0.02 | 0.00 |
| miR4406 | miR4406 | 23 | UUUAAUUUCUGGGAACCGGUGUA | 7 | 38 | 7 | 38 | 0.00 | 0.01 |
| miR4407 | miR4407 | 22 | CAGAAGGAAGCAGCAACUUGAC | 2 | 4 | 2 | 4 | 0.00 | 0.00 |
| miR4408 | miR4408 | 23 | UAACCAUUGGAUUAAGGUUGGUA | 0 | 21 | 0 | 21 | 0.00 | 0.01 |
| miR441 | miR441 | 24 | UACCAUCAAGAUAAAUCGUGGGAU | 28 | 15 | 28 | 15 | 0.01 | 0.00 |
| miR4411 | miR4411 | 24 | UUUUGUACACAAAAUUUGUCGGGU | 18 | 14 | 18 | 14 | 0.00 | 0.00 |
| miR4412 | miR4412 | 23 | AGCGGCAGAUAGAUACCCACAAC | 143 | 957 | 143 | 957 | 0.03 | 0.30 |
| miR4413 | miR4413 | 21 | AAGAUAAUUGUAAAGUGCAUG | 186 | 119 | 186 | 119 | 0.03 | 0.04 |
| miR4414 | miR4414 | 20 | AGCUGCCGACUCGUUGGUUC | 2 | 16 | 2 | 16 | 0.00 | 0.01 |
| miR4415 | miR4415 | 19 | CAGUUGUGAUGCGUCAAUG | 15 | 60 | 15 | 60 | 0.00 | 0.02 |
| miR442 | miR442 | 24 | GACAGUGUAAGAUUGUGAGACGAU | 27 | 32 | 27 | 32 | 0.01 | 0.01 |
| miR443 | miR443 | 24 | AUCCAAUACAAUAGAAUAUGAGAU | 57 | 183 | 57 | 183 | 0.01 | 0.06 |
| miR444 | miR444 | 21 | UUCAGUUGUUGGUCCAAGCAU | 40 | 6 | 40 | 6 | 0.01 | 0.00 |
| miR447 | miR447 | 22 | AGGGGACGAGAUAUGUUUGUUG | 601 | 3 | 601 | 3 | 0.11 | 0.00 |
| miR472 | miR472 | 21 | ACGGUCUAAUAGGCAAAAAUC | 148 | 0 | 148 | 0 | 0.03 | 0.00 |
| miR473 | miR473 | 21 | AUCUCCCUCAAGGGCUUCUGG | 21 | 13 | 21 | 13 | 0.00 | 0.00 |
| miR474 | miR474 | 24 | AAAAGUUGCGGUUUUGGUCCUGGG | 0 | 46 | 0 | 46 | 0.00 | 0.01 |
| miR475 | miR475a | 21 | UGACAAUGUCCAAGAUUAAUG | 22 | 7 | 32 | 13 | 0.01 | 0.00 |
|  | miR475b | 19 | UAACAGAGUCCAUUUAUUA | 10 | 6 |  |  |  |  |
| miR476 | miR476a | 20 | UAGUAAUCGUUCUUACAAA | 0 | 20 | 0 | 25 | 0.00 | 0.01 |
|  | miR476b | 24 | AUCAGUAAUUCUCUUUGGCAGAAA | 0 | 5 |  |  |  |  |
| miR477 | miR477a | 21 | AGAAGCCCUUUGGGGGAGAGG | 151 | 52 | 239 | 106 | 0.04 | 0.03 |
|  | miR477b | 21 | AAUCUCCCUCAAGGGCUUCUG | 53 | 41 |  |  |  |  |
|  | miR477c | 22 | CCCUCAAAGGCUUCCAAUACUC | 32 | 12 |  |  |  |  |
|  | miR477d | 22 | CUUCUGCCUUCAACGGCUUCUC | 3 | 1 |  |  |  |  |
| miR478 | miR478a | 24 | UGAGACGAGCUUUAUUUUUAGAGA | 8 | 4 | 10 | 5 | 0.00 | 0.00 |
|  | miR478b | 23 | UGACCGAGCUGCUAUUUUUGGAA | 2 | 1 |  |  |  |  |
| miR479 | miR479 | 22 | UGUGGUAUUGCUUUGGCUCAUC | 130 | 190 | 130 | 190 | 0.02 | 0.06 |
| miR480 | miR480 | 22 | AUCUCUACAACAUGACGUUAAC | 533 | 5 | 533 | 5 | 0.10 | 0.00 |
| miR481 | miR481 | 22 | AGGACCUACCUUAACAUUAAAC | 6 | 0 | 6 | 0 | 0.00 | 0.00 |
| miR482 | miR482a | 22 | UUUCCAACUCCACCCAUUCCUA | 157 | 42 | 270 | 120 | 0.05 | 0.04 |
|  | miR482b | 24 | AGUGGGAGGUGGUGUAUUUAGAAG | 46 | 33 |  |  |  |  |
|  | miR482c | 24 | AGAAUUUGGUGACAUGGCUGCUGA | 35 | 27 |  |  |  |  |
|  | miR482d | 24 | AGUGGGAGGUGGUGUACUAAGAAG | 24 | 12 |  |  |  |  |
|  | miR482e | 19 | UUUCCUAUUCCUCCCAUAC | 8 | 6 |  |  |  |  |
| miR528 | miR528 | 21 | UGGAAGGGGCAUGCAGAGGAG | 4 | 1 | 4 | 1 | 0.00 | 0.00 |
| miR529 | miR529a | 21 | AGAACCGUAGAAGUACAGCUU | 45 | 31 | 99 | 72 | 0.02 | 0.02 |
|  | miR529b | 21 | CGAAGCACAGGAGCUCAGUCC | 23 | 20 |  |  |  |  |
|  | miR529c | 24 | AGAAGAGAGAGAGCACGUAAACUU | 14 | 10 |  |  |  |  |
|  | miR529d | 21 | AAAAGGAGAGAGCACAUCUUG | 10 | 7 |  |  |  |  |
|  | miR529e | 19 | AGAAGAGAGUAGACAGCUU | 5 | 3 |  |  |  |  |
|  | miR529f | 21 | CAGAAGAGAGUGAGCACAGGC | 2 | 1 |  |  |  |  |
| miR530 | miR530a | 21 | UGCAUUUGCACCUGCACCUUC | 240 | 119 | 252 | 127 | 0.05 | 0.04 |
|  | miR530b | 24 | AGGUGGCAGACUGGCAGAUGGCAA | 12 | 8 |  |  |  |  |
| miR531 | miR531 | 18 | CUCGAGGGGCUGGUACCG | 0 | 178 | 0 | 178 | 0.00 | 0.06 |
| miR533 | miR533 | 21 | CCACAGUUGUAGCAAGCUCUC | 5 | 1 | 5 | 1 | 0.00 | 0.00 |
| miR535 | miR535 | 21 | UGACAAGAGACAGAGCACGUU | 240 | 13 | 240 | 13 | 0.04 | 0.00 |
| miR536 | miR536 | 19 | UCAAGUCAGCUGUGUGAUC | 861 | 122 | 861 | 122 | 0.16 | 0.04 |
| miR773 | miR773 | 21 | GAUCGUUUGACAAACAGUUCA | 34 | 5 | 34 | 5 | 0.01 | 0.00 |
| miR774 | miR774a | 24 | UGAGGCAUGAAGAUAUGGAUGAAU | 20,226 | 18,252 | 20,298 | 18,297 | 3.76 | 5.79 |
|  | miR774b | 20 | CAUGCAUAUUUUCAUCUGGA | 45 | 32 |  |  |  |  |
|  | miR774c | 23 | UGAGACGGAGAUAUGGGUAGACA | 23 | 12 |  |  |  |  |
|  | miR774d | 21 | AGAAUGGUGACUAAUACUUUU | 4 | 1 |  |  |  |  |
| miR777 | miR777 | 24 | UACGCAUACUGAGGUUCUUGCUUU | 2 | 5 | 2 | 5 | 0.00 | 0.00 |
| miR778 | miR778 | 22 | UGGCUUGGUUGUGAAGUACACG | 92 | 0 | 92 | 0 | 0.02 | 0.00 |
| miR779 | miR779 | 24 | GAUUGGAAAUUUCGGCUUGACAAU | 16 | 10 | 16 | 10 | 0.00 | 0.00 |
| miR782 | miR782 | 19 | ACAACUCUUUGGAUGUUUU | 0 | 32 | 0 | 32 | 0.00 | 0.01 |
| miR783 | miR783 | 22 | GUGCCUUUGGCUGGUCAUUUUC | 2 | 0 | 2 | 0 | 0.00 | 0.00 |
| miR808 | miR808 | 24 | AUGAAGUGGGAAAAUGUAAGACGG | 983 | 190 | 983 | 190 | 0.18 | 0.06 |
| miR809 | miR809 | 24 | AUGAAGGUGUGAGAGAGUUAGAAU | 4 | 4 | 4 | 4 | 0.00 | 0.00 |
| miR812 | miR812 | 24 | ACGGAAUUAAAGUUCGUGAGACGG | 60 | 15 | 60 | 15 | 0.01 | 0.00 |
| miR814 | miR814 | 24 | CACUUCCAAAGUACAACGAAUAAU | 2 | 3 | 2 | 3 | 0.00 | 0.00 |
| miR815 | miR815 | 24 | AAGCGGAGACUGAGCGAGAUUGGG | 23 | 59 | 23 | 59 | 0.00 | 0.02 |
| miR816 | miR816 | 21 | AGUGACAUAAUAUUAUACAAC | 111 | 0 | 111 | 0 | 0.02 | 0.00 |
| miR818 | miR818 | 24 | AAUCGACUAUAUAUUUGGGACGGA | 1,142 | 2,012 | 1,142 | 2,012 | 0.21 | 0.64 |
| miR819 | miR819 | 20 | GAGGUUAAAGACUUUCAAGC | 42 | 0 | 42 | 0 | 0.01 | 0.00 |
| miR821 | miR821a | 18 | AAGUAUAACAUAAAUGUG | 44 | 11 | 59 | 21 | 0.01 | 0.01 |
|  | miR821b | 21 | AAGUAAUGCAAAAUAAAAGUU | 12 | 9 |  |  |  |  |
|  | miR821c | 20 | AGUCACAACAACACAAGAUG | 3 | 1 |  |  |  |  |
| miR822 | miR822 | 21 | UGCGGGAAGCAUUUGCACAUG | 2 | 0 | 2 | 0 | 0.00 | 0.00 |
| miR823 | miR823 | 24 | AGGUCUGUUGAUCAUAUUAGGUAU | 22 | 269 | 22 | 269 | 0.00 | 0.09 |
| miR824 | miR824 | 21 | AGGCUCAUUUGGGAGAAGGGA | 64 | 950 | 64 | 950 | 0.01 | 0.30 |
| miR825 | miR825 | 21 | UCUUCAAUGAAGUGCAUGAAC | 138 | 202 | 138 | 202 | 0.03 | 0.06 |
| miR827 | miR827a | 21 | UUAGAUGAUCAUCAACAAACA | 70 | 42 | 114 | 80 | 0.02 | 0.03 |
|  | miR827b | 20 | UUUUUGAUUGUCACUAUAUC | 25 | 21 |  |  |  |  |
|  | miR827c | 21 | UUAGAUGAUCAUCAACAAACU | 12 | 10 |  |  |  |  |
|  | miR827d | 22 | UGAGGAUACCAUCAACAAACAA | 7 | 7 |  |  |  |  |
| miR828 | miR828a | 22 | AGAUGCUCAUUUGGGCAAGCAA | 1 | 1 | 3 | 2 | 0.00 | 0.00 |
|  | miR828b | 21 | UCUUGCAUCAAAUGAAUAUUA | 2 | 1 |  |  |  |  |
| miR829 | miR829a | 18 | AUUUGAAUCUGAUUUGCA | 22 | 37 | 43 | 74 | 0.01 | 0.02 |
|  | miR829b | 19 | CAAAUAAACUGAAAGGUAC | 10 | 18 |  |  |  |  |
|  | miR829c | 22 | CUCUGAUACCAAUUGAAGGAAU | 7 | 13 |  |  |  |  |
|  | miR829d | 21 | CAAAUUGGAAGCUUCAAGGAC | 4 | 6 |  |  |  |  |
| miR830 | miR830 | 18 | UACUAUUUUGAAAAACUG | 148 | 12 | 148 | 12 | 0.03 | 0.00 |
| miR831 | miR831 | 24 | AGGAAGAGGGACCAAUGAGAUGAA | 47 | 29 | 47 | 29 | 0.01 | 0.01 |
| miR833 | miR833a | 24 | GUGUUGUUGUACUCGGUCUGACGU | 6 | 9 | 11 | 15 | 0.00 | 0.00 |
|  | miR833b | 22 | UUAGACUGAGUCAUCAAACAAG | 5 | 6 |  |  | 0.00 | 0.00 |
| miR835 | miR835 | 22 | UUCUUGGCAUAGUUCUUUUUUC | 215 | 137 | 215 | 137 | 0.04 | 0.04 |
| miR837 | miR837 | 24 | AUGAACACGAACAAAAAAUGAUGG | 123 | 178 | 123 | 178 | 0.02 | 0.06 |
| miR838 | miR838 | 21 | UCCAAGAAUGGAAUGCAAGGC | 1 | 36 | 1 | 36 | 0.00 | 0.01 |
| miR839 | miR839 | 24 | GAAGCAUGAGCAGGAUUAGGUGAG | 17 | 12 | 17 | 12 | 0.00 | 0.00 |
| miR840 | miR840 | 24 | ACUACUGAAGAUGGCCUAAACAAC | 67 | 56 | 67 | 56 | 0.01 | 0.02 |
| miR841 | miR841a | 24 | UAGAGACCACUUGAAGACAUUGAA | 149 | 162 | 172 | 178 | 0.03 | 0.06 |
|  | miR841b | 20 | CACCGAGCCACUGGACUGAA | 23 | 16 |  |  |  |  |
| miR842 | miR842 | 19 | AUAUGAAUCCGACCUGACG | 397 | 6 | 397 | 6 | 0.07 | 0.00 |
| miR844 | miR844 | 19 | GAUAAGAUUGCUAUAAACU | 49 | 92 | 49 | 92 | 0.01 | 0.03 |
| miR845 | miR845a | 20 | UAGCUCUGAUACCAAUUUAA | 6,755 | 7,216 | 7,601 | 8,285 | 1.41 | 2.62 |
|  | miR845b | 24 | AGGCUCUGAUACCAGGAUUCCGAG | 431 | 592 |  |  |  |  |
|  | miR845c | 24 | UGGUCUCUGAAUACCAACUGAUGC | 390 | 438 |  |  |  |  |
|  | miR845d | 24 | UGCUCUGAUACCAAACUGAUACGG | 25 | 39 |  |  |  |  |
| miR846 | miR846a | 21 | UUUGAAUUGAAGAGUUGAAUU | 193 | 72 | 240 | 105 | 0.04 | 0.03 |
|  | miR846b | 19 | UUCGUGAUUUCAAUUCAAA | 47 | 33 |  |  |  |  |
| miR847 | miR847 | 19 | UCGAUGAAGAAGGAAUGAA | 29 | 0 | 29 | 0 | 0.01 | 0.00 |
| miR848 | miR848 | 24 | CUGACGAUGGGAUAACAGAAUAGA | 393 | 531 | 393 | 531 | 0.07 | 0.17 |
| miR851 | miR851 | 23 | UGUGGUAUGGAACAAAGACAACA | 3 | 3 | 3 | 3 | 0.00 | 0.00 |
| miR852 | miR852 | 24 | AGGAAUCAAAGGCAGCUUAUCAUU | 3 | 418 | 3 | 418 | 0.00 | 0.13 |
| miR854 | miR854 | 22 | GAUGAGGAUAGUAGAGGAUGGA | 43 | 29 | 43 | 29 | 0.01 | 0.01 |
| miR855 | miR855 | 22 | AGAAAGGCGAAGGAAAAUGGAA | 15 | 5 | 15 | 5 | 0.00 | 0.00 |
| miR856 | miR856 | 23 | UGAAUGUGAUUGGUAGGUAUAAA | 77 | 130 | 77 | 130 | 0.01 | 0.04 |
| miR857 | miR857a | 23 | AAACUUGUAAGCAUAACAAAAUA | 9 | 13 | 19 | 36 | 0.00 | 0.01 |
|  | miR857b | 22 | UGUUGUAUGAUGAAGGUGUGAU | 5 | 12 |  |  |  |  |
|  | miR857c | 19 | AAAUUGCACAAUACAAAAU | 3 | 6 |  |  |  |  |
|  | miR857d | 21 | AGAACUGUCACCAUACAAAUU | 2 | 5 |  |  |  |  |
| miR858 | miR858 | 21 | UUCGUUGUCUGUUCGACCUUA | 411 | 572 | 411 | 572 | 0.08 | 0.18 |
| miR859 | miR859 | 21 | UGAUUUUACCAAAGAUGAUGA | 37 | 90 | 37 | 90 | 0.01 | 0.03 |
| miR860 | miR860 | 20 | AUGAAUAGUGGACUAUAUAU | 6,403 | 5,835 | 6,403 | 5,835 | 1.19 | 1.85 |
| miR861 | miR861 | 20 | GAGGAUCUUCUGUCAAGAAC | 23 | 24 | 23 | 24 | 0.00 | 0.01 |
| miR862 | miR862 | 24 | UCCAAUAGUCAGACGCGAUGUGCU | 404 | 184 | 404 | 184 | 0.07 | 0.06 |
| miR863 | miR863 | 24 | UAUGUCUUCGUUGAUUCUUCGAAU | 1 | 126 | 1 | 126 | 0.00 | 0.04 |
| miR864 | miR864 | 18 | UCAGGUAGAUGACUCAAC | 1,704 | 1,112 | 1,704 | 1,112 | 0.32 | 0.35 |
| miR865 | miR865 | 21 | AUGAAUUGAAUACGAAUUGAG | 33 | 13 | 33 | 13 | 0.01 | 0.00 |
| miR866 | miR866a | 18 | ACAACACCACUUUGAAGA | 364 | 0 | 373 | 0 | 0.07 | 0.00 |
|  | miR866b | 22 | UCAAGGAACGGAAUUUUCUAAA | 9 | 0 |  |  |  |  |
| miR867 | miR867 | 20 | UUGAACAUGGAUGUAUGGAA | 14 | 136 | 14 | 136 | 0.00 | 0.04 |
| miR869 | miR869 | 21 | CCUGAGGUUUGAGCUAAUUAC | 278 | 1 | 278 | 1 | 0.05 | 0.00 |
| miR893 | miR893 | 22 | AGCUUGGGACUUGUACUGGGAA | 38 | 33 | 38 | 33 | 0.01 | 0.01 |
| miR894 | miR894 | 20 | GUUUCACGUCGGGUUCACCA | 431 | 289 | 431 | 289 | 0.08 | 0.09 |
| miR898 | miR898 | 18 | UUCUGUGCAUACGUAGUA | 0 | 61 | 0 | 61 | 0.00 | 0.02 |
| miR899 | miR899 | 24 | AACUGAAAGAUACAUGCAAGUUCG | 145 | 205 | 145 | 205 | 0.03 | 0.06 |
| miR900 | miR900 | 21 | UUAAUCUUAGUACCUGGGAAG | 90 | 48 | 90 | 48 | 0.02 | 0.02 |
| miR902 | miR902a | 18 | AGACAGUCUGCAUAUAGC | 1,057 | 519 | 1,173 | 590 | 0.22 | 0.19 |
|  | miR902b | 24 | AGAAGGGUACUACACAUACAACAA | 32 | 24 |  |  |  |  |
|  | miR902c | 21 | AUAUGAUGCAGAUACUUAACU | 41 | 31 |  |  |  |  |
|  | miR902d | 23 | ACGAAGGUCUGCAUUUCAUGGUA | 43 | 16 |  |  |  |  |
| miR904 | miR904 | 24 | UUUGUCAAUGUACUUGAGUGGGCA | 25 | 21 | 25 | 21 | 0.00 | 0.01 |
| miR905 | miR905 | 20 | AGGUUCCCUAGGAUAUGGCC | 21 | 0 | 21 | 0 | 0.00 | 0.00 |
| miR908 | miR908 | 22 | UGACCGUUUGACAGCAGACAUC | 839 | 1,060 | 839 | 1,060 | 0.16 | 0.34 |
| miR909 | miR909 | 21 | UUCGCAAACAUGACCCGAAUC | 6 | 6 | 6 | 6 | 0.00 | 0.00 |
| miR911 | miR911 | 21 | ACAAUGUAGUACGGUUCCUUU | 0 | 28 | 0 | 28 | 0.00 | 0.01 |
| miR913 | miR913 | 23 | AGGGACUCGCAGGCUGCGCAAGA | 18 | 0 | 18 | 0 | 0.00 | 0.00 |
| miR916 | miR916 | 19 | AGAGGUCAUCGGUUCGAUC | 20 | 119 | 20 | 119 | 0.00 | 0.04 |
| miR918 | miR918 | 23 | UACCUGAAUAGCGGACAUCGUGA | 0 | 23 | 0 | 23 | 0.00 | 0.01 |
| miR919 | miR919 | 20 | AAUCGAGGCUGAACGGAGAU | 380 | 0 | 380 | 0 | 0.07 | 0.00 |
| miR946 | miR946 | 20 | UUUGGAUGAGGAAGGGUUAU | 522 | 296 | 522 | 296 | 0.10 | 0.09 |
| miR948 | miR948 | 20 | UCAGGUGUGUGGGAUGUGAG | 328 | 0 | 328 | 0 | 0.06 | 0.00 |
| miR952 | miR952 | 21 | AACGAGAAUGAGCCAUUGCUG | 13 | 787 | 13 | 787 | 0.00 | 0.25 |
| ***Rehmannia*-specific (Group III)** | | | | | | | | | |
| miR5573 | rgl-miR5573 | 21 | GAGUAGUCGACCUGAGAUGGA | 12 | 8 | 12 | 8 | 0.00 | 0.00 |
| miR5574 | rgl-miR5574 | 21 | UUUAAUCAGAAUCUAAAAGCA | 9 | 3 | 9 | 3 | 0.00 | 0.00 |
| miR5575 | rgl-miR5575 | 21 | UGGAUUUUGAACGUUUCGGUG | 36 | 16 | 36 | 16 | 0.01 | 0.01 |
| miR5576 | rgl-miR5576 | 23 | AGAAGUUGGCAUUUGCAAACACU | 10 | 0 | 10 | 0 | 0.00 | 0.00 |
| miR5577 | rgl-miR5577 | 21 | AGAAGCUGAGAAUCACUUUUU | 78 | 56 | 78 | 56 | 0.01 | 0.02 |
| miR5578 | rgl-miR5578 | 23 | CAUGUGGCAUGAUGGCAGAAAGU | 33 | 24 | 33 | 24 | 0.01 | 0.01 |

| Table D. Different expression miRNA profiles from the FP and SP *R. glutinosa*. | | | | | | | | | |
| --- | --- | --- | --- | --- | --- | --- | --- | --- | --- |
| **miRNA family** | **miR-name** | **Sequences** | **Reads number** | | **Normalized values(TPM)** | | **Fold-change(log_2_SP/FP)** | **P-value** | **Sig-lable** |
|  |  |  | FP | SP | FP | SP |  |  |  |
| miR1027 | miR1027 | UUUUAUUUUCUAUUCCAUC | 0 | 73 | 0.01 | 4.67 | 8.87 | 1.21E-21 | ** |
| miR1028 | miR1028 | UGCAUUGUAGGUUUAAUAGAGG | 7 | 58 | 0.48 | 3.71 | 2.95 | 1.32E-10 | ** |
| miR1030 | miR1030a | UCUGCAUUUGCACCUGCACCU | 46 | 15 | 3.14 | 0.96 | -1.71 | 1.86E-05 | ** |
| miR1039 | miR1039 | GUGCGAGACGGUCUCAAGGAU | 3,805 | 329 | 260.07 | 21.03 | -3.63 | 0 | ** |
| miR1040 | miR1040 | CGAACACUAAAUGAACAUGUUCA | 6 | 62 | 0.41 | 3.96 | 3.27 | 2.92E-12 | ** |
| miR1042 | miR1042a | GGUGAUGCAGGAACUAACAGG | 272 | 1 | 18.59 | 0.06 | -8.18 | 1.72E-84 | ** |
| miR1051 | miR1051 | GGUUAAGUGAAGAAGAAGUA | 75 | 0 | 5.13 | 0.01 | -9.00 | 1.99E-24 | ** |
| miR1052 | miR1052 | UUACUUUUAUGAUUGGUUGUA | 28 | 3 | 1.91 | 0.19 | -3.32 | 1.04E-06 | ** |
| miR1060 | miR1060 | UUUGCCAAGGAUUACAAG | 43 | 0 | 2.94 | 0.01 | -8.20 | 2.54E-14 | ** |
| miR1063 | miR1063 | AUAUUGAGAAUACUGCAUCUU | 764 | 0 | 52.22 | 0.01 | -12.35 | 5.03E-242 | ** |
| miR1070 | miR1070 | UGGCUUCUAAAAAACUUGC | 0 | 106 | 0.01 | 6.78 | 9.40 | 4.18E-31 | ** |
| miR1073 | miR1073 | AGGGACUGUUAAUACAUUUCAU | 1,903 | 28 | 130.07 | 1.79 | -6.18 | 0 | ** |
| miR1074 | miR1074 | AGGGAUGUGAUGUUGUGUUGA | 54 | 1,100 | 3.69 | 70.31 | 4.25 | 1.12E-239 | ** |
| miR1077 | miR1077 | AGGGAAGGACGUAACAGAC | 94 | 0 | 6.42 | 0.01 | -9.33 | 1.99E-30 | ** |
| miR1082 | miR1082 | GUGUUGGGCUGGGCCGGCA | 330 | 1 | 22.56 | 0.06 | -8.46 | 1.00E-102 | ** |
| miR1083 | miR1083 | AGCCUGGAUCGAAGCACGCCGU | 104 | 0 | 7.11 | 0.01 | -9.47 | 1.38E-33 | ** |
| miR1087 | miR1087 | UACAGAUUUUGUAGUGCAUU | 11 | 180 | 0.75 | 11.51 | 3.94 | 2.22E-38 | ** |
| miR1097 | miR1097 | GUAGCAUUGUUGUAUGUUGGAUGA | 20 | 45 | 1.37 | 2.88 | 1.07 | 4.47E-04 | ** |
| miR1105 | miR1105 | UUUGGAUGUGGAAGACGCUC | 127 | 0 | 8.68 | 0.01 | -9.76 | 7.52E-41 | ** |
| miR1106 | miR1106 | UUUAGAAGGUGGAUGUGUGA | 184 | 0 | 12.58 | 0.01 | -10.30 | 7.50E-59 | ** |
| miR1109 | miR1109 | UAGAGGGAGGAUUUUGUGCUAA | 57 | 3,243 | 3.90 | 207.30 | 5.73 | 0 | ** |
| miR1114 | miR1114 | AAGGGACAAAAAAGGAGCAG | 37 | 168 | 2.53 | 10.74 | 2.09 | 1.78E-19 | ** |
| miR1115 | miR1115 | UGAGCUCGGCACUUUGGGAAG | 49 | 10 | 3.35 | 0.64 | -2.39 | 4.03E-08 | ** |
| miR1118 | miR1118a | CACUGACUUAUGAUAUUGGAGGGA | 262 | 41 | 17.91 | 2.62 | -2.77 | 3.97E-44 | ** |
| miR1122 | miR1122 | UAAAAACAUCCAUGUAUCUAGA | 50 | 0 | 3.42 | 0.01 | -8.42 | 1.56E-16 | ** |
| miR1125 | miR1125 | AACAACUGAACCAACUAGCGGCUG | 0 | 120 | 0.01 | 7.67 | 9.58 | 4.04E-35 | ** |
| miR1126 | miR1126 | UCAAAUCUGGACUAAUACGGAGAG | 10 | 40 | 0.68 | 2.56 | 1.90 | 4.13E-05 | ** |
| miR1137 | miR1137 | UUGUCACGAAGUUGAGUCAU | 159 | 0 | 10.87 | 0.01 | -10.09 | 5.89E-51 | ** |
| miR1138 | miR1138 | GCUUAGACGGACAUCCUCUAAAUA | 2 | 45 | 0.14 | 2.88 | 4.40 | 3.56E-11 | ** |
| miR1144 | miR1144 | UGGAACCGGGCAGCUCGGAUG | 2,315 | 192 | 158.23 | 12.27 | -3.69 | 0 | ** |
| miR1147 | miR1147 | AUAUCGGCCAAGUGGCAGA | 0 | 141 | 0.01 | 9.01 | 9.82 | 3.85E-41 | ** |
| miR1153 | miR1153 | AGAUUGUAUAUCAGAUGGCUC | 153 | 11 | 10.46 | 0.70 | -3.89 | 1.53E-35 | ** |
| miR1154 | miR1154 | AUUAGUCAUCGCCAAGGCUA | 4 | 44 | 0.27 | 2.81 | 3.36 | 3.18E-09 | ** |
| miR1156 | miR1156 | UUUUCACGGGAGCUUCAGGCAC | 88 | 38 | 6.01 | 2.43 | -1.31 | 1.13E-06 | ** |
| miR1158 | miR1158 | ACUUGGAGAGGUCACUUG | 0 | 50 | 0.01 | 3.20 | 8.32 | 4.76E-15 | ** |
| miR1159 | miR1159 | AAAUGCGAAUGGAGAUGGA | 659 | 50 | 45.04 | 3.20 | -3.82 | 7.41E-146 | ** |
| miR1160 | miR1160 | CGAGAAGGAAGACAGACGGAU | 4,297 | 5 | 293.69 | 0.32 | -9.84 | 0 | ** |
| miR1162 | miR1162 | CGGCUUAAUUUGACUCAACACG | 0 | 131 | 0.01 | 8.37 | 9.71 | 2.84E-38 | ** |
| miR1163 | miR1163 | GGGGCAUGUGCAUGCCAGG | 299 | 2 | 20.44 | 0.13 | -7.32 | 4.39E-91 | ** |
| miR1164 | miR1164 | UGGUGCAAAGGACCUGAGUGGUGU | 55 | 0 | 3.76 | 0.01 | -8.55 | 4.12E-18 | ** |
| miR1166 | miR1166 | AUAGGUCCAGACUUCAUGGG | 0 | 16 | 0.01 | 1.02 | 6.68 | 2.67E-05 | ** |
| miR1169 | miR1169 | UGUGGAUGCUUGCUUUGAUGGCU | 22 | 0 | 1.50 | 0.01 | -7.23 | 1.09E-07 | ** |
| miR1172 | miR1172 | AGGAUUGCACAGCAGAGGAGGC | 50 | 0 | 3.42 | 0.01 | -8.42 | 1.56E-16 | ** |
| miR1217 | miR1217 | AAUUUGAAGAUGAUGAACAAG | 264 | 4 | 18.04 | 0.26 | -6.14 | 6.28E-77 | ** |
| miR1222 | miR1222a | GUUGAGAGUUCGAUUGGUAUA | 9 | 382 | 0.62 | 24.42 | 5.31 | 2.46E-95 | ** |
| miR1223 | miR1223b | UUGUAGACGUCAUGUACUCGG | 0 | 35 | 0.01 | 2.24 | 7.81 | 9.52E-11 | ** |
| miR1309 | miR1309 | UGUGAGCCCUUUUGAGAUGGACA | 0 | 104 | 0.01 | 6.65 | 9.38 | 1.56E-30 | ** |
| miR1310 | miR1310 | GGCAUCGGGGGCGCAACGCC | 531 | 185 | 36.29 | 11.83 | -1.62 | 5.48E-45 | ** |
| miR1313 | miR1313 | UACACCUGAAUUAUUGUCUCG | 33 | 0 | 2.26 | 0.01 | -7.82 | 3.66E-11 | ** |
| miR1315 | miR1315 | UGGAGGCUGCACGUUGCCA | 0 | 17 | 0.01 | 1.09 | 6.76 | 1.38E-05 | ** |
| miR1318 | miR1318 | UCAGGAAGGAGACACCGA | 650 | 38 | 44.43 | 2.43 | -4.19 | 3.28E-154 | ** |
| miR1319 | miR1319 | AGAACCGGCUCUGAAUAUAUAUA | 3 | 17 | 0.21 | 1.09 | 2.41 | 0.002460556 | ** |
| miR1428 | miR1428a | AAGACAAUGGCCAUGAAUCUG | 229 | 15 | 15.65 | 0.96 | -4.03 | 8.33E-54 | ** |
| miR1432 | miR1432 | GAGGUGUCAACUGCUCUGAAGGCA | 59 | 0 | 4.03 | 0.01 | -8.66 | 2.25E-19 | ** |
| miR1435 | miR1435 | AUACUUAUCAAACUUUUU | 97 | 7 | 6.63 | 0.45 | -3.89 | 5.38E-23 | ** |
| miR1436 | miR1436 | ACUAUAUAUUUGGGACGGAGGGAG | 906 | 182 | 61.92 | 11.63 | -2.41 | 5.14E-127 | ** |
| miR1438 | miR1438 | AGGGAUACAUUUUAUAAUUUUGAA | 14 | 36 | 0.96 | 2.30 | 1.27 | 0.003848138 | ** |
| miR1439 | miR1439 | UAAUUGGGACGGAGUGAGUAUUA | 117 | 269 | 8.00 | 17.19 | 1.10 | 6.94E-13 | ** |
| miR1441 | miR1441 | CGGAUGUAGGAAAAGGUUU | 0 | 76 | 0.01 | 4.86 | 8.92 | 1.67E-22 | ** |
| miR1444 | miR1444 | UCGCAAUUCGUCAAAUGUUC | 194 | 11 | 13.26 | 0.70 | -4.24 | 2.09E-47 | ** |
| miR1446 | miR1446 | UUCCUGAACUGCUUCCCUGAA | 0 | 64 | 0.01 | 4.09 | 8.68 | 4.61E-19 | ** |
| miR1449 | miR1449 | AUGAGGGCACGAUCAAAAUAACUC | 7 | 126 | 0.48 | 8.05 | 4.07 | 6.60E-28 | ** |
| miR1507 | miR1507 | AGGUUGUGAUUGGAUACGAAAGAA | 25 | 7 | 1.71 | 0.45 | -1.93 | 0.000671815 | ** |
| miR1508 | miR1508a | UAGACAGAGGGAAUAAAGUUG | 574 | 79 | 39.23 | 5.05 | -2.96 | 2.88E-101 | ** |
| miR1510 | miR1510a | AAGAGGAUUAGGUAUAAACAAAC | 20 | 2 | 1.37 | 0.13 | -3.42 | 3.43E-05 | ** |
|  | miR1510b | UGUGUUUUACUCUAUUUCCCC | 16 | 1 | 1.09 | 0.06 | -4.10 | 8.20E-04 | ** |
| miR1512 | miR1512 | CAAUCUGAAAAUUCCAAAGUAU | 42 | 6 | 2.87 | 0.38 | -2.90 | 1.60E-08 | ** |
| miR1515 | miR1515 | UCAUUUUUGCGUGCAGUGAUCC | 21 | 5 | 1.44 | 0.32 | -2.17 | 0.000830718 | ** |
| miR1518 | miR1518 | UGUGUUGUAAAGAUGAAAUGACUC | 7 | 45 | 0.48 | 2.88 | 2.59 | 1.45E-07 | ** |
| miR1519 | miR1519 | UAGGAGUGUUACAAAAUUAGUCAU | 21 | 0 | 1.44 | 0.01 | -7.17 | 2.25E-07 | ** |
| miR1522 | miR1522 | UUGUAUGGCUUAAUGAAAU | 677 | 95 | 46.27 | 6.07 | -2.93 | 5.55E-118 | ** |
| miR1523 | miR1523 | AGGGGAUAAAGUGAGACUA | 319 | 147 | 21.80 | 9.40 | -1.21 | 1.81E-18 | ** |
| miR1528 | miR1528 | AUUCAUUAGAUCGACAUAUAUUAU | 40 | 5 | 2.73 | 0.32 | -3.10 | 1.28E-08 | ** |
| miR1531 | miR1531 | UCUCAAUCUGGAAGACUUGUC | 0 | 37 | 0.01 | 2.37 | 7.89 | 2.54E-11 | ** |
| miR1533 | miR1533 | AUAAUAAAUAUAGAUAUA | 200 | 66 | 13.67 | 4.22 | -1.70 | 4.50E-19 | ** |
| miR1534 | miR1534 | UAAUUUUCGGUAAAUUGCAU | 65 | 14 | 4.44 | 0.89 | -2.31 | 5.17E-10 | ** |
| miR1536 | miR1536 | AAGUAGAGAGAAAUGUGUUUUAG | 37 | 0 | 2.53 | 0.01 | -7.98 | 2.00E-12 | ** |
| miR156 | miR156a | UGACAGAAGAGAGUGAGCAU | 13,778 | 6,893 | 941.71 | 440.61 | -1.10 | 0 | ** |
|  | miR156b | CACGACAGAUAGAAAGCACAAU | 860 | 123 | 58.75 | 7.80 | -2.82 | 0 | ** |
|  | miR156d | UGCUCAUUUCUCUUUCUGUCAG | 44 | 10 | 3.01 | 0.63 | -2.04 | 0 | ** |
|  | miR156g | UGACAGAAGAGAGAGAGCAU | 22 | 7 | 1.50 | 0.45 | -1.75 | 0.0029386 | ** |
| miR157 | miR157a | UUGACAGAAGAUAGAGAGCAC | 136,153 | 27,475 | 9440.91 | 1769.35 | -2.42 | 0 | ** |
|  | miR157b | UGACAGAAGAUAGAGAGCAC | 2,049 | 408 | 139.96 | 26.01 | -2.80 | 0 | ** |
|  | miR157c | GCUCUCUAUGCUUCUGUCAUCA | 446 | 177 | 83.45 | 11.32 | -2.88 | 0.000008 | ** |
| miR158 | miR158a | UCCCAAAUGUAGACAAAGCA | 25 | 140 | 1.71 | 8.95 | 2.39 | 3.56E-19 | ** |
| miR160 | miR160a | GCGUAUGAGGAGCGAAGCAUA | 328 | 1,476 | 21.95 | 82.37 | 1.91 | 6.91E-191 | ** |
|  | miR160c | GUGUACGAGGAGCCAAGCAUG | 28 | 126 | 1.86 | 7.89 | 2.09 | 9.53E-18 | ** |
| miR163 | miR163b | UGAAGAGGGACUGCAUCUCGAUCA | 5 | 63 | 0.34 | 4.03 | 3.56 | 2.87E-13 | ** |
| miR165 | miR165a | UCGGACCAGGCUUCAUCCCCC | 91 | 39 | 6.22 | 2.49 | -1.32 | 6.27E-07 | ** |
|  | miR165b | GAAGUGUUCGGAUCGAGGC | 27 | 9 | 1.85 | 0.58 | -1.68 | 0.001296047 | ** |
| miR167 | miR167a | UGAAGCUGCCAGCAUGAUCUA | 18,234 | 6,686 | 1246.16 | 427.37 | -1.54 | 3.66E-177 |  |
|  | miR167b | GGUCAUGCUCUGACAGCAUCACU | 968 | 402 | 66.16 | 25.70 | -1.36 | 5.58E-161 |  |
|  | miR167d | GUUCUAGUACGACCGUCGAAU | 498 | 237 | 34.03 | 15.15 | -1.16 | 1.24e-315 |  |
|  | miR167e | GAUCAUGUGGUAGCUUCAUC | 445 | 58 | 30.41 | 3.71 | -3.36 | 5.89E-28 |  |
| miR168 | miR168 | UCGCUUGGUGCAGGUCGGGAA | 5,613 | 1,188 | 383.64 | 75.94 | -2.34 | 0 | ** |
| miR170 | miR170 | AUAUAGGCCUAGUUCACUCAG | 145 | 438 | 9.91 | 28.00 | 1.50 | 3.53E-31 | ** |
| miR1846 | miR1846a | UAUCCGACGUACGCAGGGAGGC | 1 | 27 | 0.07 | 1.73 | 4.66 | 2.72E-07 | ** |
| miR1850 | miR1850a | UGCAAAGGUUUGGAGAUUGGG | 56 | 11 | 3.83 | 0.70 | -2.44 | 2.59E-09 | ** |
| miR1851 | miR1851 | GGGUCUGGGAUGGAUUUGGC | 0 | 80 | 0.01 | 5.11 | 9.00 | 1.19E-23 | ** |
| miR1855 | miR1855 | AGCACUGUGAGAUACACCAAGAGA | 90 | 344 | 6.15 | 21.99 | 1.84 | 1.21E-32 | ** |
| miR1856 | miR1856 | UAUGCGAAGACGGACGAA | 88 | 331 | 6.01 | 21.16 | 1.81 | 5.88E-31 | ** |
| miR1858 | miR1858 | AGAGGAGGAGGAGGAGGGGC | 135 | 0 | 9.23 | 0.01 | -9.85 | 2.24E-43 | ** |
| miR1860 | miR1860 | AGAAAACAAGCAUUCCGAACU | 0 | 95 | 0.01 | 6.07 | 9.25 | 5.96E-28 | ** |
| miR1861 | miR1861b | CGAGUGUUGUGGCAAGAAUGAC | 27 | 90 | 1.85 | 5.75 | 1.64 | 2.21E-08 | ** |
| miR1862 | miR1862 | AUAAGUUUGUAUUAUUUUUGGGAC | 5 | 28 | 0.34 | 1.79 | 2.39 | 8.56E-05 | ** |
| miR1867 | miR1867 | UGUUUUUUUGUAGAGAGAGGGGU | 0 | 23 | 0.01 | 1.47 | 7.20 | 2.63E-07 | ** |
| miR1870 | miR1870 | AUUGGGCUAACUUCAGAUGACCA | 141 | 24 | 9.64 | 1.53 | -2.65 | 2.39E-23 | ** |
| miR1871 | miR1871 | AGGCUCUGAUACCAGUUGGGGUU | 757 | 6 | 51.74 | 0.38 | -7.08 | 4.24E-227 | ** |
| miR1872 | miR1872 | GAACUGUAGUAUGGACAGGUAUA | 68 | 7 | 4.65 | 0.45 | -3.38 | 7.66E-15 | ** |
| miR1873 | miR1873 | UCAACUGGUAUCAGAGCCUGAA | 60 | 29 | 4.10 | 1.85 | -1.15 | 0.000301621 | ** |
| miR1875 | miR1875 | AACAAUGUAGAUGUGCAACAGAA | 725 | 37 | 49.55 | 2.37 | -4.39 | 3.63E-177 | ** |
| miR1878 | miR1878 | AACUUAACUAGGACACUUAGAAGA | 20 | 192 | 1.37 | 12.27 | 3.17 | 3.00E-34 | ** |
| miR1888 | miR1888 | UAAGUUAGAUUUAUGAAGGAU | 0 | 559 | 0.01 | 35.73 | 11.80 | 5.40E-161 | ** |
| miR1917 | miR1917 | UUAAUAAAAUUGUAAAGU | 451 | 6 | 30.83 | 0.38 | -6.33 | 8.52E-132 | ** |
| miR2079 | miR2079 | AGAGUAUGAUGUUAUGACG | 9 | 21 | 0.62 | 1.34 | 1.13 | 0.046362242 | ** |
| miR2080 | miR2080 | UAUACAAUUUGCAGAUGC | 119 | 0 | 8.13 | 0.01 | -9.67 | 2.53E-38 | ** |
| miR2083 | miR2083 | AGAUUGACGACGUGUACAAGAUGA | 31 | 68 | 2.12 | 4.35 | 1.04 | 0.000650028 | ** |
| miR2090 | miR2090 | AACUCUGAUCUAGAAGUCUUGUGU | 35 | 8 | 2.39 | 0.51 | -2.23 | 9.57E-06 | ** |
| miR2095 | miR2095 | CUGAAAUUUUACUAGAUGAACAUG | 0 | 23 | 0.01 | 1.47 | 7.20 | 2.63E-07 | ** |
| miR2099 | miR2099 | UGAAGAUGUUUGUAAAGCUUAUAA | 24 | 8 | 1.64 | 0.51 | -1.68 | 2.47E-03 | ** |
| miR2101 | miR2101 | AGUUUGUAACUCAAGUGGUAUUGU | 1,334 | 77 | 91.18 | 4.92 | -4.21 | 1.23e-315 | ** |
| miR2105 | miR2105 | UGUGUGUUGUGAAUGAUUGUAU | 55 | 6 | 3.76 | 0.38 | -3.29 | 5.34E-12 | ** |
| miR2108 | miR2108 | AUUAAUGUGUAUGUGUUUGGGUCG | 3 | 32 | 0.21 | 2.05 | 3.32 | 6.12E-07 | ** |
| miR2118 | miR2118a | UUCCAACUCCACCCAUUCCUA | 148 | 1 | 10.12 | 0.06 | -7.31 | 1.37E-45 | ** |
|  | miR2118b | UUCCUGAGCCUUCUGUUUCCUA | 49 | 12 | 3.35 | 0.77 | -2.12 | 7.90E-04 | ** |
| miR2119 | miR2119 | UACAAUGGGAGAUGGUAGGGGAA | 51 | 0 | 3.49 | 0.01 | -8.45 | 7.56E-17 | ** |
| miR2120 | miR2120 | AAAGACUUUAGUGACGUGUUGUU | 552 | 0 | 37.73 | 0.01 | -11.88 | 4.51E-175 | ** |
| miR2123 | miR2123 | AAGAAGAUCAACGGUGUGCAACAC | 24 | 0 | 1.64 | 0.01 | -7.36 | 2.54E-08 | ** |
| miR2586 | miR2586 | CGAGGAAUGUCGUGCUUGCAUC | 3 | 39 | 0.21 | 2.49 | 3.60 | 1.02E-08 | ** |
| miR2592 | miR2592 | AAAUGACUUGAGUGAUGUGUGCUU | 135 | 3,413 | 9.23 | 218.16 | 4.56 | 0 | ** |
| miR2593 | miR2593 | UUAGAAUAGAAGAUGAACCUAAAU | 100 | 16 | 6.83 | 1.02 | -2.74 | 1.54E-17 | ** |
| miR2595 | miR2595 | CAUUUUCUUCUUUAUACU | 190 | 48 | 12.99 | 3.07 | -2.08 | 1.58E-23 | ** |
| miR2600 | miR2600 | AAUUGCAAUCACAAGGCC | 74 | 0 | 5.06 | 0.01 | -8.98 | 4.12E-24 | ** |
| miR2605 | miR2605 | ACUUAUUUAUAUGAAAUA | 59 | 0 | 4.03 | 0.01 | -8.66 | 2.25E-19 | ** |
| miR2607 | miR2607 | AUGUGAUUAUUGAUUGUG | 72 | 233 | 4.92 | 14.89 | 1.60 | 7.49E-19 | ** |
| miR2608 | miR2608 | AGUUGACAUAUAUCAUUACCUCAU | 23 | 0 | 1.57 | 0.01 | -7.30 | 5.27E-08 | ** |
| miR2611 | miR2611 | UAUUUGUCUGUUGGAUCAA | 12 | 117 | 0.82 | 7.48 | 3.19 | 1.53E-21 | ** |
| miR2616 | miR2616 | AGUUCGGUAUGGUUCGGGACGGAU | 0 | 436 | 0.01 | 27.87 | 11.44 | 1.00E-125 | ** |
| miR2619 | miR2619 | AACAUGAGGAGGCUUUUUGUAAUU | 140 | 0 | 9.57 | 0.01 | -9.90 | 5.90E-45 | ** |
| miR2620 | miR2620 | UCUGAAGACACCAGCUCUGAC | 0 | 87 | 0.01 | 5.56 | 9.12 | 1.17E-25 | ** |
| miR2621 | miR2621 | AGCUGGGCUAGAAAUUGUC | 36 | 0 | 2.46 | 0.01 | -7.94 | 4.13E-12 | ** |
| miR2630 | miR2630 | UGGUUUUGCCUUAUAUUU | 49 | 2 | 3.35 | 0.13 | -4.71 | 1.19E-13 | ** |
| miR2635 | miR2635 | AUAUUCUCACAUGACUAG | 315 | 0 | 21.53 | 0.01 | -11.07 | 3.18E-100 | ** |
| miR2637 | miR2637 | AAAUAUCUUCCUCAUGACUCCUGA | 0 | 155 | 0.01 | 9.91 | 9.95 | 3.72E-45 | ** |
| miR2638 | miR2638 | AUAUAAUAUGUGCAGUGG | 91 | 4 | 6.22 | 0.26 | -4.60 | 4.35E-24 | ** |
| miR2639 | miR2639 | UUGUCGGCUUACGUAAUUG | 17 | 0 | 1.16 | 0.01 | -6.86 | 4.13E-06 | ** |
| miR2641 | miR2641 | UUUUGAUCUUUUCGUUUA | 0 | 123 | 0.01 | 7.86 | 9.62 | 5.58E-36 | ** |
| miR2642 | miR2642 | AGAGUAUCUUCAAAUCAGU | 37 | 0 | 2.53 | 0.01 | -7.98 | 2.00E-12 | ** |
| miR2654 | miR2654 | AUUGAGGGACGAAAGUGUG | 0 | 132 | 0.01 | 8.44 | 9.72 | 1.47E-38 | ** |
| miR2655 | miR2655 | AGUUUAGGUCCUUUAACUUUUGA | 0 | 23 | 0.01 | 1.47 | 7.20 | 2.63E-07 | ** |
| miR2658 | miR2658 | AUGGACAUUGUAUAUGAGAC | 126 | 28 | 8.61 | 1.79 | -2.27 | 9.53E-18 | ** |
| miR2660 | miR2660 | UAAGAUCAUCAGCAUAAACCA | 0 | 138 | 0.01 | 8.82 | 9.78 | 2.79E-40 | ** |
| miR2661 | miR2661 | UGGGUUUGAGAAAGUGGGC | 83 | 33 | 5.67 | 2.11 | -1.43 | 4.37E-07 | ** |
| miR2662 | miR2662 | GAGAAAAAUGUAAGACGGAAU | 6,008 | 0 | 410.64 | 0.01 | -15.33 | 0 | ** |
| miR2663 | miR2663 | UUAAGAGGGCGUUUCAAAUU | 68 | 3 | 4.65 | 0.19 | -4.60 | 2.77E-18 | ** |
| miR2666 | miR2666 | UAAAGUCAGGAUUAUCAAGGA | 68 | 152 | 4.65 | 9.72 | 1.06 | 1.71E-07 | ** |
| miR2672 | miR2672 | UUAAGUCGAAACAAUGGGUACUA | 68 | 0 | 4.65 | 0.01 | -8.86 | 3.23E-22 | ** |
| miR2675 | miR2675 | CGAGGCAUACAUUGAAGGAUU | 28 | 5 | 1.91 | 0.32 | -2.58 | 1.67E-05 | ** |
| miR2678 | miR2678 | UGGAAAUUGUCGCAGUGUCUC | 4 | 93 | 0.27 | 5.94 | 4.44 | 4.60E-22 | ** |
| miR2862 | miR2862 | UAACGGCUUAGAUUUGUCC | 103 | 0 | 7.04 | 0.01 | -9.46 | 2.86E-33 | ** |
| miR2868 | miR2868 | UUCUUUGUUUAGUAGAAA | 68 | 31 | 4.65 | 1.98 | -1.23 | 4.64E-05 | ** |
| miR2870 | miR2870 | AAUACAUUUCGGGAGACAAA | 0 | 137 | 0.01 | 8.76 | 9.77 | 5.40E-40 | ** |
| miR2875 | miR2875 | AUUUUCAGUCAUUACUAGUUAUA | 32 | 0 | 2.19 | 0.01 | -7.77 | 7.57E-11 | ** |
| miR2878 | miR2878 | AUACAUGUACAAAUUUCGAGGAUG | 28 | 3 | 1.91 | 0.19 | -3.32 | 1.04E-06 | ** |
| miR2905 | miR2905 | UACUGUAGUGACACAGCGCA | 4 | 317 | 0.27 | 20.26 | 6.21 | 3.21E-84 | ** |
| miR2916 | miR2916 | UGGGGGCUCGAAGACGAUCAGA | 671 | 334 | 45.86 | 21.35 | -1.10 | 4.90E-32 | ** |
| miR2920 | miR2920 | AAGAUCAACAAUUAAAUUUCAA | 174 | 5 | 11.89 | 0.32 | -5.22 | 6.08E-48 | ** |
| miR2922 | miR2922 | AAAUAAGUGAUGACGAAAUC | 0 | 32 | 0.01 | 2.05 | 7.68 | 6.90E-10 | ** |
| miR2925 | miR2925 | AUGGGCGGCCGCGUGGCUUGGU | 19 | 0 | 1.30 | 0.01 | -7.02 | 9.65E-07 | ** |
| miR2926 | miR2926 | AGGACUCGACGUUGGUGA | 24 | 10 | 1.64 | 0.64 | -1.36 | 0.00957462 | ** |
| miR2927 | miR2927 | UGUGUCACGUCGACGGAGCCCUG | 422 | 3 | 28.84 | 0.19 | -7.23 | 9.13E-128 | ** |
| miR2931 | miR2931 | AUUUAUUGUUCGAUGAAAA | 13 | 541 | 0.89 | 34.58 | 5.28 | 4.16E-134 | ** |
| miR2936 | miR2936 | CUGUGAGAAGAAGAGAACCAGAUG | 36 | 9 | 2.46 | 0.58 | -2.10 | 1.52E-05 | ** |
| miR2937 | miR2937 | AGAAGAAGCUGUUGAAGGAGGAC | 0 | 43 | 0.01 | 2.75 | 8.10 | 4.84E-13 | ** |
| miR319 | miR319 | UUGGACUGAAGGGAGCUCCC | 132 | 63 | 9.02 | 4.03 | -1.16 | 5.19E-08 | ** |
| miR3438 | miR3438 | UACAAGGAUUUAGCUAAAGC | 0 | 55 | 0.01 | 3.52 | 8.46 | 1.75E-16 | ** |
| miR3440 | miR3440 | UGGAUUGGUUCAAAUGAGACCGCA | 211 | 38 | 14.42 | 2.43 | -2.57 | 4.43E-33 | ** |
| miR3443 | miR3443 | UUGCCUUGGAGAUGGUAGAGUU | 0 | 19 | 0.01 | 1.21 | 6.92 | 3.68E-06 | ** |
| miR3445 | miR3445 | UUUGGAGGUGAGUUGUUUGC | 1 | 2,128 | 0.07 | 136.02 | 10.96 | 0 | ** |
| miR3446 | miR3446 | AUGAAGCUAGCACUGUGGCAGG | 0 | 208 | 0.01 | 13.30 | 10.38 | 2.37E-60 | ** |
| miR3447 | miR3447 | CGUGACGGACAAAUAAAG | 438 | 5 | 29.94 | 0.32 | -6.55 | 2.38E-129 | ** |
| miR3449 | miR3449 | AGAUAGUAAACGAAUACUGGAUA | 191 | 98 | 13.05 | 6.26 | -1.06 | 1.24E-09 | ** |
| miR3451 | miR3451 | CGCGAUCCGGGAGGUUCACAA | 285 | 0 | 19.48 | 0.01 | -10.93 | 9.49E-91 | ** |
| miR3461 | miR3461 | UCUGACUCGCUGAGCAGAA | 1,171 | 0 | 80.04 | 0.01 | -12.97 | 0 | ** |
| miR3463 | miR3463 | AGCAGCGGAGCUGGAAGGGG | 101 | 52 | 6.90 | 3.32 | -1.05 | 1.11E-05 | ** |
| miR3464 | miR3464 | UGGGUAUGGGUUGGAGAUGG | 686 | 44 | 46.89 | 2.81 | -4.06 | 5.75E-159 | ** |
| miR3465 | miR3465 | CAACUGAAGACUAGAAGGA | 11,671 | 77 | 797.70 | 4.92 | -7.34 | 0 | ** |
| miR3467 | miR3467 | UGCGGCUGAGAAGACCAGA | 231 | 0 | 15.79 | 0.01 | -10.62 | 1.08E-73 | ** |
| miR3509 | miR3509 | AUACUUGAAUCCAGACUUAGAUGA | 0 | 20 | 0.01 | 1.28 | 7.00 | 1.90E-06 | ** |
| miR3512 | miR3512 | UGGCAAAUGAUGACAAAAUAG | 16 | 103 | 1.09 | 6.58 | 2.59 | 9.91E-16 | ** |
| miR3513 | miR3513 | UAAAUUUCUGAGCUUGAUGCAUC | 15 | 111 | 1.03 | 7.10 | 2.79 | 3.82E-18 | ** |
| miR3520 | miR3520 | AGGGAUGGUGAAUAUGCUUCUCAU | 46 | 5 | 3.14 | 0.32 | -3.30 | 3.04E-10 | ** |
| miR3521 | miR3521 | UGGUGACUUGUAUACAUAUG | 27 | 9 | 1.85 | 0.58 | -1.68 | 0.001296047 | ** |
| miR3623 | miR3623 | UGGAUGGCUUCGGAGAAUUUGCAA | 1,148 | 3 | 78.46 | 0.19 | -8.68 | 0 | ** |
| miR3625 | miR3625 | CGGGAGAUGAUGAUACUGUAAGC | 247 | 98 | 16.88 | 6.26 | -1.43 | 2.04E-18 | ** |
| miR3627 | miR3627 | UCUGUCCAGGAGAGCACGGCCU | 0 | 24 | 0.01 | 1.53 | 7.26 | 1.36E-07 | ** |
| miR3629 | miR3629 | CCAUUUUCUCGAGCCAAC | 187 | 481 | 12.78 | 30.75 | 1.27 | 8.97E-27 | ** |
| miR3633 | miR3633 | AGAAUGAUGGUUAGAAGG | 266 | 23 | 18.18 | 1.47 | -3.63 | 1.79E-57 | ** |
| miR3637 | miR3637 | AUUUUUCGUAUUGUGUUUUGUCAG | 3 | 93 | 0.21 | 5.94 | 4.86 | 3.84E-23 | ** |
| miR3638 | miR3638 | ACAACAAGACAUGAUAAGGACACC | 0 | 28 | 0.01 | 1.79 | 7.48 | 9.68E-09 | ** |
| miR3640 | miR3640 | CACAUGAUUGGUAUGCUUUUUU | 77 | 0 | 5.26 | 0.01 | -9.04 | 4.65E-25 | ** |
| miR3693 | miR3693 | UAGGGUUGCUCAUGAACUAGCUC | 43 | 0 | 2.94 | 0.01 | -8.20 | 2.54E-14 | ** |
| miR3704 | miR3704 | AGGAUCUAGGCGGGUUGGAAUAAA | 61 | 0 | 4.17 | 0.01 | -8.70 | 5.25E-20 | ** |
| miR3705 | miR3705 | GUCAGUGUUUGUCUGGAC | 96 | 0 | 6.56 | 0.01 | -9.36 | 4.64E-31 | ** |
| miR3706 | miR3706 | UAUAGAGAAAUGGUAAGA | 0 | 574 | 0.01 | 36.69 | 11.84 | 2.70E-165 | ** |
| miR3711 | miR3711 | CGGCGCUAGAAGGAGGAUCCAU | 8 | 65 | 0.55 | 4.15 | 2.93 | 1.22E-11 | ** |
| miR3932 | miR3932 | AACUUUGUGACUGAAAAAGAG | 496 | 44 | 33.90 | 2.81 | -3.59 | 6.56E-105 | ** |
| miR3933 | miR3933 | AGAGAGACAAAAGUGACGACUCAG | 36 |  | 2.46 | 0.38 | -2.68 | 5.59E-07 | ** |
| miR3946 | miR3946 | UGAGAAAAAGAGAAGAAGAGCA | 79 | 14 | 5.40 | 0.89 | -2.59 | 2.18E-13 | ** |
| miR3948 | miR3948 | UGGAUGUGGGAGGGGUAGGUG | 62 | 5 | 4.24 | 0.32 | -3.73 | 1.05E-14 | ** |
| miR3949 | miR3949 | UGAUGAUGAGGCAGAAAAUGAG | 37 | 1,200 | 2.53 | 76.71 | 4.92 | 2.11E-285 | ** |
| miR3951 | miR3951 | UAGAUAAUCAGAUAGAGAAAACAG | 166 | 1,835 | 11.35 | 117.29 | 3.37 | 0 | ** |
| miR3954 | miR3954 | UGGACAAACUCACGGUCA | 190 | 18 | 12.99 | 1.15 | -3.50 | 3.07E-40 | ** |
| miR397 | miR397a | UUGAGUGCAGCGUUGAUGAUA | 99 | 2 | 6.77 | 0.13 | -5.73 | 7.34E-29 | ** |
|  | miR397b | UCAUUGAGUGCAGCGUUGAUG | 52 | 12 | 3.55 | 0.77 | -2.21 | 0.00034 | ** |
| miR398 | miR398a | GGGGCAACAUGAGAACAUAUA | 329 | 21 | 22.49 | 1.34 | -4.07 | 3.67E-77 | ** |
|  | miR398d | GCAGUAGUCAUAUGAGAACACGGA | 23 | 0 | 1.57 | 0.01 | -7.30 | 5.60E-06 | ** |
| miR400 | miR400 | GACUGGUAUAAUAAUUCAUUGAAG | 15 | 4 | 1.03 | 0.26 | -2.00 | 0.007690881 | ** |
| miR407 | miR407 | UUUAUUAUGCAUAUCUUUUGGU | 9 | 100 | 0.62 | 6.39 | 3.38 | 1.62E-19 | ** |
| miR408 | miR408a | ACAGAGACGAGACAGAGCAUG | 12,495 | 408 | 854.02 | 26.08 | -5.03 | 0 | ** |
|  | miR408d | AUGCACUGCCUCUUCCCUGGC | 65 | 27 | 2.93 | 1.16 | -1.33 | 9.70E-06 | ** |
| miR413 | miR413 | AUAGUUUCUUUUGUUUAC | 60 | 16 | 4.10 | 1.02 | -2.00 | 5.02E-08 | ** |
| miR414 | miR414 | UCAUCAUCAUCAUCAUCUGC | 29 | 0 | 1.98 | 0.01 | -7.63 | 6.71E-10 | ** |
| miR415 | miR415a | AACUGAGCAGCAACCAGAACA | 1,618 | 790 | 110.59 | 50.50 | -1.13 | 8.47E-78 | ** |
| miR419 | miR419a | UGAUGAAGACGACGAUGA | 2,091 | 274 | 142.92 | 17.51 | -3.03 | 0 | ** |
| miR420 | miR420 | AAACUAGAUCACGGAAGCA | 38 | 12 | 2.60 | 0.77 | -1.76 | 7.60E-05 | ** |
| miR4223 | miR4223 | ACUGGAAUUUGAUUAGAAGAAACA | 139 | 15 | 9.50 | 0.96 | -3.31 | 1.84E-28 | ** |
| miR4225 | miR4225 | AAUGCGAUGGUUAAAACGAACGAC | 9 | 22 | 0.62 | 1.41 | 1.19 | 0.032580912 | ** |
| miR4226 | miR4226 | AUACAAGAUGAUGCAUGAGCAAUA | 15 | 238 | 1.03 | 15.21 | 3.89 | 6.89E-50 | ** |
| miR4235 | miR4235 | ACGACAGCAGCUACAACCCCU | 41 | 114 | 2.80 | 7.29 | 1.38 | 2.90E-08 | ** |
| miR4243 | miR4243 | UGAAAUUGUGUAUUUGUAC | 0 | 75 | 0.01 | 4.79 | 8.91 | 3.23E-22 | ** |
| miR4245 | miR4245 | ACAACCUUUUAUCUGACAAAU | 156 | 0 | 10.66 | 0.01 | -10.06 | 5.22E-50 | ** |
| miR4248 | miR4248 | AAAUUUUAUUUUUGGACAAAUC | 0 | 95 | 0.01 | 6.07 | 9.25 | 5.96E-28 | ** |
| miR4250 | miR4250 | UCCAAAGGCAAGAACAAA | 1,081 | 31 | 73.88 | 1.98 | -5.22 | 1.13E-290 | ** |
| miR4341 | miR4341 | GGUGGAAAGUUUAACAAUGACGGA | 15 | 0 | 1.03 | 0.01 | -6.68 | 1.77E-05 | ** |
| miR4342 | miR4342 | AAGGUGAUUAGAAUUAGGAUGGU | 5 | 20 | 0.34 | 1.28 | 1.90 | 0.004274687 | ** |
| miR4347 | miR4347 | AAGACUGUCUUACGGAUCAGGAU | 43 | 0 | 2.94 | 0.01 | -8.20 | 2.54E-14 | ** |
| miR4348 | miR4348 | AAAACUGUGUAAGAUGGUCUCAUU | 30 | 13 | 2.05 | 0.83 | -1.30 | 0.00492173 | ** |
| miR4350 | miR4350 | UGCAAAGGCACAAGAGACAGACA | 0 | 103 | 0.01 | 6.58 | 9.36 | 3.03E-30 | ** |
| miR4351 | miR4351 | UUUGGGAUUCAGUCUGGAGAUG | 3,453 | 0 | 236.01 | 0.01 | -14.53 | 0 | ** |
| miR4358 | miR4358 | AGUGCAUGAACUAUAUUGGCCUAG | 0 | 25 | 0.01 | 1.60 | 7.32 | 7.02E-08 | ** |
| miR4360 | miR4360 | CAGUUGCACGGUGCGACGGAUUGC | 101 | 3 | 6.90 | 0.19 | -5.17 | 3.25E-28 | ** |
| miR4366 | miR4366 | CAUAUUAUGUAGUAGAUUUGAUGG | 1 | 139 | 0.07 | 8.89 | 7.02 | 9.90E-39 | ** |
| miR4369 | miR4369 | GGUCAAGCUGAUCAAGGAACGGA | 0 | 18 | 0.01 | 1.15 | 6.85 | 7.13E-06 | ** |
| miR4371 | miR4371a | GAUUUGAAGACGGAAUAUCCAU | 167 | 18 | 11.41 | 1.15 | -3.31 | 6.37E-34 | ** |
|  | miR4371b | AAUGAUGCAGAUGACAAGGAAGG | 43 | 6 | 2.94 | 0.38 | -2.87 | 5.29E-08 | ** |
| miR4374 | miR4374 | UAAGACGGUCGCAUGAUUCAACGA | 0 | 27 | 0.01 | 1.73 | 7.43 | 1.87E-08 | ** |
| miR4379 | miR4379 | UAGAUGUGACUGGUGAGAGGCC | 85 | 16 | 5.81 | 1.02 | -2.51 | 8.16E-14 | ** |
| miR4382 | miR4382 | UAUUUACACAUAGAUUUCAUGCAU | 7 | 24 | 0.48 | 1.53 | 1.68 | 0.003857872 | ** |
| miR4384 | miR4384 | AAUCAGACACAUGCAUCGAGACGA | 13 | 82 | 0.89 | 5.24 | 2.56 | 1.20E-12 | ** |
| miR4386 | miR4386 | UGAAGAGUUCUGGAAGAUCUGCA | 0 | 219 | 0.01 | 14.00 | 10.45 | 1.66E-63 | ** |
| miR4388 | miR4388 | AGAACUUAUGGGACCAAAUUGCAC | 42 | 16 | 2.87 | 1.02 | -1.49 | 0.000221621 | ** |
| miR4391 | miR4391 | CUGGCAAAGAACCAAGAGGAGAAG | 67 | 159 | 4.58 | 10.16 | 1.15 | 1.23E-08 | ** |
| miR4393 | miR4393a | UGACGAAAAGGAGCGGAAAACC | 14 | 54 | 0.96 | 3.45 | 1.85 | 2.69E-06 | ** |
| miR4397 | miR4397 | UGCAAGAUGUGGCGAAUU | 0 | 414 | 0.01 | 26.46 | 11.37 | 2.03E-119 | ** |
| miR4404 | miR4404 | GAUUCUGGAAGACUGAAGGAUAA | 104 | 0 | 7.11 | 0.01 | -9.47 | 1.38E-33 | ** |
| miR4406 | miR4406 | UUUAAUUUCUGGGAACCGGUGUA | 7 | 38 | 0.48 | 2.43 | 2.34 | 5.28E-06 | ** |
| miR4408 | miR4408 | UAACCAUUGGAUUAAGGUUGGUA | 0 | 21 | 0.01 | 1.34 | 7.07 | 9.84E-07 | ** |
| miR4412 | miR4412 | AGCGGCAGAUAGAUACCCACAAC | 143 | 957 | 9.77 | 61.17 | 2.65 | 5.33E-137 | ** |
| miR4414 | miR4414 | AGCUGCCGACUCGUUGGUUC | 2 | 16 | 0.14 | 1.02 | 2.90 | 0.001200392 | ** |
| miR4415 | miR4415 | CAGUUGUGAUGCGUCAAUG | 15 | 60 | 1.03 | 3.84 | 1.90 | 4.49E-07 | ** |
| miR443 | miR443 | AUCCAAUACAAUAGAAUAUGAGAU | 57 | 183 | 3.90 | 11.70 | 1.59 | 5.60E-15 | ** |
| miR444 | miR444 | UUCAGUUGUUGGUCCAAGCAU | 40 | 6 | 2.73 | 0.38 | -2.83 | 5.29E-08 | ** |
| miR447 | miR447 | AGGGGACGAGAUAUGUUUGUUG | 601 | 3 | 41.08 | 0.19 | -7.74 | 7.69E-184 | ** |
| miR472 | miR472 | ACGGUCUAAUAGGCAAAAAUC | 148 | 0 | 10.12 | 0.01 | -9.98 | 1.76E-47 | ** |
| miR474 | miR474 | AAAAGUUGCGGUUUUGGUCCUGGG | 0 | 46 | 0.01 | 2.94 | 8.20 | 6.68E-14 | ** |
| miR475 | miR475a | UGACAAUGUCCAAGAUUAAUG | 22 | 7 | 1.50 | 0.45 | -1.75 | 0.002938585 | ** |
| miR476 | miR476a | UAGUAAUCGUUCUUACAAA | 0 | 20 | 0.01 | 1.28 | 7.00 | 1.90E-06 | ** |
| miR477 | miR477a | AGAAGCCCUUUGGGGGAGAGG | 151 | 52 | 10.32 | 3.32 | -1.63 | 4.46E-14 | ** |
|  | miR477c | CCCUCAAAGGCUUCCAAUACUC | 32 | 12 | 2.05 | 0.77 | -1.41 | 9.60E-05 | ** |
| miR480 | miR480 | AUCUCUACAACAUGACGUUAAC | 533 | 5 | 36.43 | 0.32 | -6.83 | 6.25E-159 | ** |
| miR482 | miR482a | UUUCCAACUCCACCCAUUCCUA | 157 | 42 | 10.73 | 2.68 | -2.00 | 9.17E-19 | ** |
|  | miR482d | AGUGGGAGGUGGUGUACUAAGAAG | 24 | 12 | 1.57 | 0.38 | -2.04 | 6.50E-05 | ** |
| miR530 | miR530a | UGCAUUUGCACCUGCACCUUC | 240 | 119 | 16.40 | 7.61 | -1.11 | 1.57E-12 | ** |
| miR531 | miR531 | CUCGAGGGGCUGGUACCG | 0 | 178 | 0.01 | 11.38 | 10.15 | 9.47E-52 | ** |
| miR535 | miR535 | UGACAAGAGACAGAGCACGUU | 240 | 13 | 16.40 | 0.83 | -4.30 | 6.56E-59 | ** |
| miR536 | miR536 | UCAAGUCAGCUGUGUGAUC | 861 | 122 | 58.85 | 7.80 | -2.92 | 7.90E-149 | ** |
| miR773 | miR773 | GAUCGUUUGACAAACAGUUCA | 34 | 5 | 2.32 | 0.32 | -2.86 | 4.90E-07 | ** |
| miR778 | miR778 | UGGCUUGGUUGUGAAGUACACG | 92 | 0 | 6.29 | 0.01 | -9.30 | 8.51E-30 | ** |
| miR782 | miR782 | ACAACUCUUUGGAUGUUUU | 0 | 32 | 0.01 | 2.05 | 7.68 | 6.90E-10 | ** |
| miR808 | miR808 | AUGAAGUGGGAAAAUGUAAGACGG | 983 | 190 | 67.19 | 12.15 | -2.47 | 2.31E-141 | ** |
| miR812 | miR812 | ACGGAAUUAAAGUUCGUGAGACGG | 60 | 15 | 4.10 | 0.96 | -2.10 | 1.98E-08 | ** |
| miR815 | miR815 | AAGCGGAGACUGAGCGAGAUUGGG | 23 | 59 | 1.57 | 3.77 | 1.26 | 0.000204585 | ** |
| miR816 | miR816 | AGUGACAUAAUAUUAUACAAC | 111 | 0 | 7.59 | 0.01 | -9.57 | 8.50E-36 | ** |
| miR819 | miR819 | GAGGUUAAAGACUUUCAAGC | 42 | 0 | 2.87 | 0.01 | -8.17 | 5.26E-14 | ** |
| miR821 | miR821a | AAGUAUAACAUAAAUGUG | 44 | 11 | 3.01 | 0.70 | -2.10 | 1.64E-06 | ** |
| miR823 | miR823 | AGGUCUGUUGAUCAUAUUAGGUAU | 22 | 269 | 1.50 | 17.19 | 3.52 | 6.46E-52 | ** |
| miR824 | miR824 | AGGCUCAUUUGGGAGAAGGGA | 64 | 950 | 4.37 | 60.72 | 3.80 | 7.41E-191 | ** |
| miR830 | miR830 | UACUAUUUUGAAAAACUG | 148 | 12 | 10.12 | 0.77 | -3.72 | 2.86E-33 | ** |
| miR838 | miR838 | UCCAAGAAUGGAAUGCAAGGC | 1 | 36 | 0.07 | 2.30 | 5.07 | 9.29E-10 | ** |
| miR842 | miR842 | AUAUGAAUCCGACCUGACG | 397 | 6 | 27.13 | 0.38 | -6.14 | 4.53E-115 | ** |
| miR846 | miR846a | UUUGAAUUGAAGAGUUGAAUU | 193 | 72 | 13.19 | 4.60 | -1.52 | 5.85E-16 | ** |
| miR847 | miR847 | UCGAUGAAGAAGGAAUGAA | 29 | 0 | 1.98 | 0.01 | -7.63 | 6.71E-10 | ** |
| miR852 | miR852 | AGGAAUCAAAGGCAGCUUAUCAUU | 3 | 418 | 0.21 | 26.72 | 7.03 | 2.05E-114 | ** |
| miR855 | miR855 | AGAAAGGCGAAGGAAAAUGGAA | 15 | 5 | 1.03 | 0.32 | -1.68 | 0.017800967 | ** |
| miR859 | miR859 | UGAUUUUACCAAAGAUGAUGA | 37 | 90 | 2.53 | 5.75 | 1.19 | 1.19E-05 | ** |
| miR862 | miR862 | UCCAAUAGUCAGACGCGAUGUGCU | 404 | 184 | 27.61 | 11.76 | -1.23 | 1.89E-23 | ** |
| miR863 | miR863 | UAUGUCUUCGUUGAUUCUUCGAAU | 1 | 126 | 0.07 | 8.05 | 6.88 | 4.80E-35 | ** |
| miR865 | miR865 | AUGAAUUGAAUACGAAUUGAG | 33 | 13 | 2.26 | 0.83 | -1.44 | 0.001441016 | ** |
| miR866 | miR866a | ACAACACCACUUUGAAGA | 364 | 0 | 24.88 | 0.01 | -11.28 | 1.07E-115 | ** |
| miR867 | miR867 | UUGAACAUGGAUGUAUGGAA | 14 | 136 | 0.96 | 8.69 | 3.18 | 8.76E-25 | ** |
| miR869 | miR869 | CCUGAGGUUUGAGCUAAUUAC | 278 | 1 | 19.00 | 0.06 | -8.22 | 2.24E-86 | ** |
| miR898 | miR898 | UUCUGUGCAUACGUAGUA | 0 | 61 | 0.01 | 3.90 | 8.61 | 3.34E-18 | ** |
| miR900 | miR900 | UUAAUCUUAGUACCUGGGAAG | 90 | 48 | 6.15 | 3.07 | -1.00 | 6.86E-05 | ** |
| miR902 | miR902a | AGACAGUCUGCAUAUAGC | 1,057 | 519 | 72.65 | 33.62 | -1.11 | 2.32E-50 | ** |
|  | miR902d | ACGAAGGUCUGCAUUUCAUGGUA | 43 | 16 | 2.93 | 1.02 | -1.51 | 0.000221621 | ** |
| miR905 | miR905 | AGGUUCCCUAGGAUAUGGCC | 21 | 0 | 1.44 | 0.01 | -7.17 | 2.25E-07 | ** |
| miR911 | miR911 | ACAAUGUAGUACGGUUCCUUU | 0 | 28 | 0.01 | 1.79 | 7.48 | 9.68E-09 | ** |
| miR913 | miR913 | AGGGACUCGCAGGCUGCGCAAGA | 18 | 0 | 1.23 | 0.01 | -6.94 | 2.00E-06 | ** |
| miR916 | miR916 | AGAGGUCAUCGGUUCGAUC | 20 | 119 | 1.37 | 7.61 | 2.48 | 3.78E-17 | ** |
| miR918 | miR918 | UACCUGAAUAGCGGACAUCGUGA | 0 | 23 | 0.01 | 1.47 | 7.20 | 2.63E-07 | ** |
| miR919 | miR919 | AAUCGAGGCUGAACGGAGAU | 380 | 0 | 25.97 | 0.01 | -11.34 | 9.44E-121 | ** |
| miR948 | miR948 | UCAGGUGUGUGGGAUGUGAG | 328 | 0 | 22.42 | 0.01 | -11.13 | 2.50E-104 | ** |
| miR952 | miR952 | AACGAGAAUGAGCCAUUGCUG | 13 | 787 | 0.89 | 50.31 | 5.82 | 1.48E-202 | ** |
| rgl-miR7797 | rgl-miR7797b | UUUGAUUUCGUCUUACAUUUUUC | 0 | 33 | 0.01 | 2.11 | 7.72 | 2.37E-07 | ** |
| rgl-miR7800 | rgl-miR7800 | UAUUUUUGUGUCGUUAUGGUC | 21 | 0 | 1.44 | 0.01 | -7.17 | 4.75E-07 | ** |
| rgl-miR7801 | rgl-miR7801 | UACGAGAUGAAACACAGUUUG | 18 | 0 | 1.23 | 0.01 | -6.94 | 3.63E-05 | ** |
| rgl-miR7804 | rgl-miR7804-5p | AGGGGUGUUCAUCGAAUCGAAUU | 24 | 0 | 1.64 | 0.01 | -7.36 | 5.47E-07 | ** |
| rgl-miR7807 | rgl-miR7807a-3p | AACUAUAUGAAAAUCUCAAUU | 64 | 24 | 4.37 | 1.53 | -1.51 | 3.15E-04 | ** |
|  | rgl-miR7807b-3p | UUGAGAUUUUCAUAUAGUUACU | 13 | 0 | 0.89 | 0.01 | -6.47 | 7.89E-07 | ** |
| rgl-miR7808 | rgl-miR7808 | AAGGAUGCUCGAUUCAGAAGAA | 26 | 0 | 1.78 | 0.01 | -7.48 | 3.15E-06 | ** |
| rgl-miR7809 | rgl-miR7809 | UCCCAUUGCAUCAGCGGACACA | 0 | 108 | 0.01 | 6.90 | 9.43 | 3.24E-06 | ** |
| rgl-miR7811 | rgl-miR7811 | UGAAUGGAGAUACGGAAUGAAGC | 0 | 369 | 0.01 | 23.59 | 11.20 | 1.21E-07 | ** |

| Table E. Summary of reads produced by degradome sequencing in FP and SP *R. glutinosa*. | | |
| --- | --- | --- |
| **Types of reads** | **Number of reads** | |
|  | **FP** | **SP** |
| total reads | 14,633,167 | 21,149,584 |
| high quality | 14,587,504 | 21,087,010 |
| adaptor3 null | 18,136 | 37,955 |
| insert null | 2,117 | 5,689 |
| adaptor 5 contaminants | 43,887 | 55,249 |
| smaller than 18nt | 2,026,115 | 995,274 |
| clean reads (unique) | 12,497,249(982,398) | 19,992,843(1,984,865) |
| Mapping to transcriptome reads (unique) | 4,738,853(367,290) | 8,415,733(769,336) |

| Table F. Target for the different expression miRNA familes from FP and SP *R. glutinosa*. | | | | | | |  |  |  |
| --- | --- | --- | --- | --- | --- | --- | --- | --- | --- |
| **miRNA families** | **miRNA fold-change(SP/FP)** | **Target gene ID** | **Score** | **Cleavage site** | **FP** | | **SP** | | **Nr-annotation** |
|  |  |  |  |  | **Category** | **Reads at cleavage(TP10M)** | **Category** | **Reads at cleavage(TP10M)** |  |
| **Up-regulated** | | | | | | | | | |
| miR1027 | 8.87 | Unigene53502_All | 2 | 79 |  | no | II | 10.02 | tRNA (adenine-N1-)-methyltransferase |
| miR1028 | 2.95 | Unigene44897_All | 3 | 61 | III | 2.21 | II | 6.93 | dentin sialophosphoprotein-related |
| miR1074 | 4.25 | Unigene56511_All | 4 | 48 |  | no | I | 7.56 | polyphenol oxidase |
| miR1087 | 3.94 | Unigene32990_All | 3 | 712 |  | no | I | 82.96 | sec23/sec24 transport family protein |
| miR1147 | 9.82 | Unigene68408_All | 3.5 | 82 | I | 12.30 | I | 31.21 | transducin family protein// WD-40 repeat family protein |
| miR1154 | 3.36 | Unigene38482_All | 3 | 13 | I | 0.12 | I | 2.83 | Cupin, RmlC-type |
| miR1222 | 5.31 | Unigene29778_All | 3 | 80 | I | 1.94 | I | 11.94 | CBL-interacting protein kinase 18 |
| miR1223 | 7.81 | Unigene27493_All | 3.5 | 496 |  | no | III | 12.77 | structural molecule |
| miR1319 | 2.41 | Unigene20180_All | 4 | 300 | III | 0.56 | III | 3.45 | lateral organ boundaries domain protein |
| miR1438 | 1.27 | Unigene413_All | 4 | 735 | III | 1.89 |  | no | type II homeodomain-leucine zipper protein |
| miR1441 | 8.92 | Unigene44745_All | 0 | 149 |  | no | I | 4.5 | XT1 (XYLOSYLTRANSFERASE 1) |
| miR1531 | 7.89 | Unigene27539_All | 3 | 130 |  | no | I | 28.4 | nbs-lrr resistance protein |
| miR160 | 2. 07 | Unigene27735_All | 2 | 386 | I | 22.89 | I | 19.65 | auxin response factor 3 |
|  |  | Unigene38897_All | 2.5 | 254 | I | 48.02 | II | 22.75 | auxin response factor 10 |
|  |  | Unigene667_All | 1 | 546 | I | 32.03 | I | 8.53 | auxin response factor 10 |
|  |  | Unigene2294_All | 1 | 1175 | I | 45.86 | I | 1.74 | auxin response factor 10 |
|  |  | Unigene9362_All | 1.5 | 1367 | I | 11.96 | I | 3.59 | auxin response factor 10 |
|  |  | Unigene11206_All | 1.5 | 373 | I | 23.65 | I | 2.63 | auxin response factor 10 |
|  |  | Unigene27735_All | 2 | 386 | III | 3.84 | III | 0.55 | auxin response factor 3 |
|  |  | Unigene65531_All | 2 | 426 | I | 2.67 | I | 21.73 | auxin response factor 10 |
| miR1851 | 9 | Unigene27779_All | 2.5 | 854 | III | 19.76 | III | 61.61 | ALY protein |
|  |  | Unigene44335_All | 4 | 10 |  | no | II | 23.95 | glycyl-tRNA synthetase |
|  |  | Unigene13825_All | 4 | 10 |  | no | II | 31.72 | glycyl-tRNA synthetase |
| miR1855 | 1.84 | Unigene40903_All | 4 | 161 | III | 2.67 |  | no | caffeoyl-CoA-O-methyltransferase |
| miR1856 | 1.81 | Unigene18207_All | 3 | 357 |  | no | I | 22.31 | unknown protein |
|  |  | Unigene26929_All | 3.5 | 570 |  | no | I | 2.1 | ACI13 |
| miR1861 | 1.64 | Unigene26519_All | 3 | 2992 |  | no | I | 39.96 | potassium ion transmembrane transporter 7 |
|  |  | Unigene35754_All | 4 | 741 |  | no | I | 2.59 | NOL1/NOP2/sun family protein |
| miR2079 | 1.13 | Unigene11294_All | 0 | 128 | III | 0.19 |  | no | carbonyl reductase -like protein |
| miR2083 | 1.04 | Unigene62142_All | 3.5 | 87 |  | no | I | 29.61 | vacuolar processing enzyme-1b |
| miR2592 | 4.56 | Unigene1844_All | 2.5 | 210 | I | 6.09 | I | 8.56 | unkown protein |
| miR2611 | 3.19 | Unigene35784_All | 2 | 258 |  | no | III | 22.33 | arginine methyltransferease |
|  |  | Unigene45133_All | 4 | 289 |  | no | I | 0.78 | unknown protein |
| miR2931 | 5.28 | Unigene44304_All | 2.5 | 32 |  | no | III | 45.61 | histidine kinase 3B |
|  |  | Unigene28866_All | 2 | 457 | I | 1.38 | I | 21.6 | EDGP precursor |
| miR3512 | 2.59 | Unigene35293_All | 3.5 | 2801 |  | no | I | 88.02 | ABC transporter family |
| miR3706 | 11.84 | Unigene36053_All | 2.5 | 706 |  | no | III | 7.23 | emb1923 (embryo defective 1923) |
| miR3951 | 3.37 | Unigene17868_All | 3 | 661 |  | no | III | 102.71 | F-box family protein |
|  |  | Unigene58241_All | 4 | 58 |  | no | I | 10.77 | Calreticulin |
|  |  | Unigene28585_All | 3 | 216 |  | no | II | 21.09 | homeobox-leucine zipper family protein |
|  |  | Unigene27303_All | 3 | 134 |  | no | II | 0.34 | Protein transport protein Sec24-like |
|  |  | Unigene32627_All | 3 | 447 |  | no | I | 12.56 | unknown protein |
|  |  | Unigene9547_All | 3 | 155 |  | no | I | 2.76 | unknown protein |
|  |  | Unigene49393_All | 2 | 121 |  | no | III | 25.98 | ER33 protein |
|  |  | Unigene10972_All | 3 | 91 |  | no | I | 6.63 | unknown protein |
|  |  | Unigene9165_All | 3 | 1814 |  | no | III | 118.95 | exonuclease family protein |
|  |  | Unigene11404_All | 3 | 182 |  | no | I | 48.01 | MYB transcription factor MYB127 |
|  |  | Unigene82055_All | 3 | 47 |  | no | II | 1.99 | hydroxyproline-rich glycoprotein family protein |
|  |  | Unigene1992_All | 3 | 86 |  | no | III | 0.23 | Unknown protein |
|  |  | Unigene24058_All | 4 | 1634 |  | no | I | 365.4 | oxidoreductase |
|  |  | Unigene36169_All | 3 | 12 | II | 13.80 | II | 0.23 | hydrolase, acting on ester bonds |
| miR4226 | 3.89 | Unigene24638_All | 2.5 | 333 | I | 0.77 |  | no | TPR Domain containing protein, expressed |
|  |  | Unigene29735_All | 2 | 341 | I | 1.95 | I | 2.34 | anthocyanin 5-O-glucoside-4'''-O-malonyltransferase |
| miR4235 | 1.38 | Unigene81195_All | 4 | 203 |  | no | I | 1.88 | aspartic protease |
| miR4366 | 7.02 | Unigene74675_All | 2 | 141 | II | 4.78 | II | 13.76 | Lipolytic enzyme, G-D-S-L |
|  |  | Unigene33370_All | 2 | 705 | II | 26.96 | II | 27.83 | Lipolytic enzyme, G-D-S-L |
| miR4369 | 6.85 | Unigene20144_All | 4 | 331 | I | 0.23 | I | 1.44 | neutral invertase |
| miR782 | 7.86 | Unigene13192_All | 3.5 | 170 |  | no | I | 0.23 | ALY protein |
|  |  | Unigene6973_All | 3.5 | 88 |  | no | I | 1.97 | unknown protein |
|  |  | Unigene7561_All | 4 | 33 |  | no | I | 12.96 | pollen-specific C2 domain containing protein |
| miR815 | 1.26 | Unigene76964_All | 2.5 | 95 |  | no | I | 0.75 | PAF1 complex component |
|  |  | Unigene74018_All | 4 | 556 |  | no | II | 0.43 | unknown protein |
|  |  | Unigene84077_All | 2.5 | 238 | II | 2.97 |  | no | Unknown protein |
| miR859 | 1.19 | Unigene6378_All | 2 | 338 | I | 12.53 |  | no | arm repeat-containing protein |
| rgl-miR7811 | 7.72 | Unigene74608_All | 4 | 1911 |  | no | II | 49.61 | structural maintenance of chromosomes family protein |
| **Down-regulated** | | | | | | | | | |
| miR1051 | -9 | Unigene66083_All | 4 | 178 |  | no | I | 1.04 | metal ion binding |
| miR1063 | -12.35 | Unigene9897_All | 3 | 131 | II | 17.98 |  | 5.87 | unknown protein |
| miR1077 | -8.46 | Unigene40733_All | 2.5 | 771 |  | no | I | 1.64 | phox (PX) domain-containing protein |
| miR1115 | -2.39 | Unigene5576_All | 3 | 103 | I | 17.51 | I | 6.92 | Probable thiol methyltransferase 2 |
|  |  | Unigene13216_All | 3.5 | 289 | I | 2.30 |  | 2.88 | transporter-related |
| miR1153 | -3.89 | Unigene20781_All | 0.5 | 82 |  | no | III | 0.42 | RNA-directed DNA polymerase |
| miR1156 | -1.31 | Unigene2469_All | 3 | 503 |  | no | III | 1.77 | similar to A. thaliana protein BAB01483 similar to kinesin light chain |
|  |  | Unigene40199_All | 3.5 | 201 |  | no | II | 0.32 | GCN5-related N-acetyltransferase (GNAT) family protein |
| miR1163 | -7.32 | Unigene32677_All | 4 | 70 |  | no | I | 1.86 | unknown protein |
|  |  | Unigene28846_All | 4 | 470 | II | 14.92 |  | no | unknown protein |
| miR1217 | -6.14 | Unigene35968_All | 4 | 255 |  | no | I | 21.65 | 2-oxoglutarate dehydrogenase E2 subunit |
|  |  | Unigene1953_All | 4 | 144 |  | no | I | 12.39 | esterase/lipase/thioesterase family protein |
| miR1310 | -1.62 | Unigene35463_All | 3.5 | 849 | I | 33.75 |  | no | f-box family protein |
| miR1428 | -4.03 | Unigene74496_All | 2 | 283 |  | no | I | 0.67 | armadillo/beta-catenin repeat family protein |
|  |  | Unigene35446_All | 2 | 1026 |  | no | III | 3.02 | Met-10+ like family protein / kelch repeat-containing protein |
| miR1436 | -2.41 | Unigene1295_All | 4 | 545 |  | no | III | 1.13 | carbohydrate kinase-like protein |
| miR1507 | -1.93 | Unigene44831_All | 4 | 188 |  | no | II | 30.01 | uridine cytidine kinase |
| miR1508 | -2.96 | Unigene1653_All | 4 | 293 |  | no | I | 4.13 | CPRD2 |
|  |  | Unigene9202_All | 2.5 | 23 |  | no | II | 5.32 | unknown protein |
| miR1528 | -3.1 | Unigene27981_All | 4 | 454 |  | no | I | 0.26 | CLP protease regulatory subunit CLPX |
|  |  | Unigene20916_All | 4 | 187 | I | 41.69 |  | no | ATB' BETA; protein phosphatase type 2A regulator |
| miR1536 | -7.98 | Unigene65645_All | 3.5 | 682 |  | no | III | 0.75 | transferase, transferring glycosyl groups |
|  |  | Unigene74304_All | 3.5 | 707 |  | no | III | 0.38 | transferase, transferring glycosyl groups |
|  |  | Unigene32537_All | 4 | 290 |  | no | I | 1.5 | oxidoreductase |
| miR156 | -1.1 | Unigene39392_All | 3 | 220 |  | no | I | 0.38 | vacuolar H(+)-ATPase subunit c |
|  |  | Unigene732_All | 4 | 292 |  | no | III | 40.32 | heat shock protein binding |
|  |  | Unigene16389_All | 3.5 | 625 |  | no | I | 6.89 | 5'-3' exonuclease family protein |
|  |  | Unigene35337_All | 2 | 953 | III | 74.03 | I | 8.94 | squamosa promoter-binding protein |
|  |  | Unigene16389_All | 2 | 625 |  | no | I | 12.75 | 5'-3' exonuclease family protein |
|  |  | Unigene65615_All | 2 | 390 |  | no | II | 45.06 | Squamosa promoter-binding-like protein 7 |
| miR157 | -2.42 | Unigene35337_All | 3 | 953 | II | 74.03 | II | 8.94 | squamosa promoter-binding protein |
|  |  | Unigene43500_All | 3.5 | 450 |  | no | I | 1.88 | CYP71D175 |
|  |  | Unigene716_All | 3 | 593 |  | no | I | 38.79 | SQUAMOSA-promoter binding protein 1 |
|  |  | Unigene18390_All | 3.5 | 907 |  | no | II | 11.31 | promoter-binding protein SPL9 |
|  |  | Unigene730_All | 2 | 987 |  | no | III | 1.94 | squamosa promoter binding protein-homologue 5 |
|  |  | Unigene40778_All | 3 | 176 |  | no | II | 1.43 | CTP synthase |
|  |  | Unigene65615_All | 1.5 | 390 |  | no | I | 17.39 | Squamosa promoter-binding-like protein 7 |
|  |  | Unigene18019_All | 3 | 522 |  | no | I | 9.86 | Ribose-phosphate pyrophosphokinase 1 |
|  |  | Unigene28753_All | 4 | 235 |  | no | III | 0.32 | regulator of chromosome condensation family protein |
| miR165 | -1.32 | Unigene9429_All | 2.5 | 793 |  | no | I | 14.96 | PHAVOLUTA-like HD-ZIPIII protein |
|  |  | Unigene19246_All | 2.5 | 883 | I | 25.01 | I | 0.52 | class III HD-Zip protein 8 |
|  |  | Unigene17711_All | 2.5 | 732 |  | no | II | 0.44 | class III HD-Zip protein 1 |
|  |  | Unigene21369_All | 3.5 | 94 |  | no | I | 7.02 | class III HD-Zip protein 5 |
| miR167 | -1.41 | Unigene6351_All | 2.5 | 434 |  | no | I | 63.52 | auxin response factor 8 |
|  |  | Unigene40790_All | 3.5 | 102 |  | no | II | 21.03 | binding protein |
|  |  | Unigene25498_All | 3 | 110 | II | 18.75 | II | 31.77 | unknown protein |
|  |  | Unigene43250_All | 3 | 368 | II | 0.24 | II | 17.89 | auxin response factor 6 |
|  |  | Unigene18276_All | 4 | 168 |  | no | III | 22.54 | ABC transporter C family member 9 |
|  |  | Unigene65770_All | 2.5 | 796 | III | 4.76 | III | 12.3 | 3-dehydroquinate dehydratase |
|  |  | Unigene21829_All | 4 | 479 |  | no | III | 4.56 | unknown protein |
| miR168 | -2.34 | Unigene45023_All | 3 | 18 | I | 30.51 | I | no | AGO1-1 |
|  |  | Unigene1196_All | 4 | 18 |  | no | I | 0.65 | AGO1-1 |
| miR1858 | -9.85 | Unigene2408_All | 2.5 | 854 |  | no | II | 1.03 | Ribonuclease H |
| miR1871 | -7.08 | Unigene14_All | 4 | 1363 |  | no | I | 2.76 | phosphoinositide binding |
| miR2105 | -3.29 | Unigene32771_All | 1 | 621 |  | no | I | 1.32 | nucleic acid binding |
| miR2118 | -7.31 | Unigene48758_All | 4 | 33 | III | 12.76 |  | no | unknown protein |
| miR2593 | -2.74 | Unigene37543_All | 0 | 397 | I | 2.95 |  | no | unknown protein |
| miR2605 | -8.66 | Unigene36320_All | 2 | 499 |  | no | III | 6.63 | EMB1025 |
| miR2619 | -9.9 | Unigene37264_All | 3 | 719 |  | no | III | 0.39 | nodulin MtN21 family protein |
| miR2621 | -7.94 | Unigene42275_All | 3.5 | 366 |  | no | I | 7.04 | f-box family protein |
| miR2663 | -4.6 | Unigene7358_All | 4 | 182 | I | 14.51 |  | no | endonuclease |
| miR2862 | -9.46 | Unigene17444_All | 4 | 117 | III | 0.21 |  | no | unknown protein |
| miR319 | -1.16 | Unigene33748_All | 3 | 78 |  | no | I | 5.98 | MdTCP2B |
|  |  | Unigene59602_All | 3 | 47 |  | no | I | 0.34 | MdTCP2B |
|  |  | Unigene63815_All | 2 | 323 | III | 28.53 | III | 11.87 | hypothetical protein |
|  |  | Unigene69675_All | 2 | 187 |  | no | III | 2.43 | GAMyb protein |
|  |  | Unigene80484_All | 3 | 16 |  | no | II | 0.77 | retrofit |
| miR3440 | -2.57 | Unigene1471_All | 3.5 | 2509 |  | no | I | 4.33 | pentatricopeptide repeat-containing protein |
|  |  | Unigene52840_All | 3.5 | 1002 |  | no | I | 0.26 | unknown protein |
| miR3449 | -1.06 | Unigene85440_All | 3.5 | 172 | II | 3.74 |  | no | receptor-like kinase |
|  |  | Unigene9806_All | 3.5 | 1463 | I | 31.86 |  | no | integral membrane transporter family protein |
| miR3461 | -12.97 | Unigene19678_All | 3 | 18 |  | no | I | 4.75 | polyphenoloxidase |
| miR3463 | -1.05 | Unigene21164_All | 3.5 | 300 | I | 1.33 |  | no | unknown protein |
| miR3464 | -4.06 | Unigene30739_All | 4 | 137 | I | 3.52 |  | no | EIL2 |
| miR3933 | -2.68 | Unigene26755_All | 2.5 | 3906 |  | no | III | 10.34 | RAPTOR1B |
| miR408 | -5.03 | Unigene26610_All | 4 | 1386 |  | no | I | 0.52 | unknown protein |
|  |  | Unigene40796_All | 3.5 | 29 | II | 14.51 |  | no | lateral organ boundaries domain protein |
| miR419 | -3.03 | Unigene37385_All | 3 | 20 |  | no | I | 0.69 | unknown protein |
|  |  | Unigene27051_All | 3.5 | 1024 | III | 23.91 |  | no | BURP domain protein |
| miR4223 | -3.31 | Unigene7543_All | 0 | 254 |  | no | II | 11.1 | CTV.15 |
|  |  | Unigene63301_All | 0 | 121 |  | no | I | 4.81 | CTV.15 |
| miR4347 | -8.2 | Unigene42636_All | 4 | 257 | II | 7.12 |  | no | PHAVOLUTA-like HD-ZIPIII protein |
| miR4388 | -1.49 | Unigene310_All | 3.5 | 2432 |  | no | I | 9.68 | F-box family protein |
| miR472 | -9.98 | Unigene70853_All | 2.5 | 137 |  | no | III | 3.64 | sulfotransferase family protein |
|  |  | Unigene26618_All | 2 | 346 |  | no | II | 0.48 | protein binding |
| miR477 | -1.63 | Unigene63464_All | 2 | 53 | I | 13.50 | I | 0.23 | RNA helicase |
|  |  | Unigene82778_All | 3 | 40 |  | no | I | 0.59 | DEAD/DEAH box helicase family protein |
|  |  | Unigene11619_All | 3.5 | 182 | I | 33.92 |  | no | vitamin C permease family protein |
| miR482 | -2 | Unigene70853_All | 2.5 | 137 |  | no | I | 14.44 | sulfotransferase family protein |
|  |  | Unigene17658_All | 3.5 | 2496 |  | no | II | 1.85 | enoyl-CoA hydratase |
| miR812 | -2.1 | Unigene14695_All | 4 | 203 |  | no | II | 0.83 | ATP-dependent RNA helicase-like protein DB10 |
|  |  | Unigene10950_All | 4 | 989 | II | 4.72 |  | no | nucleoporin 98 |
| miR821 | -2.1 | Unigene50971_All | 3.5 | 522 |  | no | II | 2.98 | similar to somatic embryogenesis receptor-like kinase 3 |
| miR846 | -1.52 | Unigene45879_All | 4 | 10 | I | 0.69 |  | no | diaminopimelate epimerase family protein |
| miR847 | -7.63 | Unigene40565_All | 2.5 | 696 |  | no | I | 1.72 | Asparagine synthetase |
|  |  | Unigene19495_All | 3.5 | 392 | I | 15.38 |  | no | similar to mRNA capping enzyme family protein |
| miR902 | -1.11 | Unigene43414_All | 4 | 360 |  | no | I | 1.65 | protein kinase MK5 |
|  |  | Unigene24097_All | 4 | 141 | I | 42.62 |  | no | Alcohol dehydrogenase-like 7 |
| miR916 | -2.48 | Unigene1653_All | 2.5 | 133 |  | no | III | 0.36 | CPRD2 |
| miR948 | -11.3 | Unigene21330_All | 2.5 | 14 | I | 3.93 | I | 2.44 | 2,3-bisphosphoglycerate-independent phosphoglycerate mutase |

Note: “TP10M” represents “tags per 10 million”.

| Table G. Forward primer sequences of 24 novel miRNAs using qRT-PCR verfication. | |
| --- | --- |
| **miRNA** | **Primer sequence** |
| rgl-miR7797a | AAGACGGAATCAAACCTCA |
| rgl-miR7797b | TTGATTTCGTCTTACATTTTTC |
| rgl-miR7798 | AGGGAGTGTTTGCAAAAACT |
| rgl-miR7799 | AGTGGAATAGGAGATCTCA |
| rgl-miR7800 | TATTTTTGTGTCGTTATGGTC |
| rgl-miR7801 | TACGAGATGAAACACAGTTTG |
| rgl-miR7802 | AGGGAGTGTTTGCAATCACTA |
| rgl-miR7803a | TACGGATAATTGACACGTGTAT |
| rgl-miR7803b-5p | GGATGATTGCCACGTGTAT |
| rgl-miR7803b-3p | TACACGTGTCAATCATCTAT |
| rgl-miR7804-5p | GGGTGTTCATCGAATCGAATT |
| rgl-miR7804-3p | TTTAATCGAATGAACATTTT |
| rgl-miR7805-5p | AAATTTGGTGTAGTGAATAGT |
| rgl-miR7805-3p | TATTCATTTACACCAAATTTGG |
| rgl-miR7806 | TAGAAGATGTCCACATGAGC |
| rgl-miR7807a-5p | CTATATGAAAATCTCAATT |
| rgl-miR7807a -3p | TTGGGATTTGCATACAGTTAC |
| rgl-miR7807b -5p | TAACTATATGAAAATCTCAAT |
| rgl-miR7807b -3p | TGAGATTTTCATATAGTTACT |
| rgl-miR7808 | AAGGATGCTCGATTCAGAAG |
| rgl-miR7809 | TCCCATTGCATCAGCGGACAC |
| rgl-miR7810 | AGAGGAAGAGTTTTCTGGCTC |
| rgl-miR7811 | TGAATGGAGATACGGAATGAAGC |
| rgl-miR7972 | TTGTCAGGCTTGTTATTCTCC |
| 18S | GAGCTAATACGTGCAACAAACC |

| Table H. Forward primer sequences of 20 confirmed Solexa sequencing different expression miRNA using qRT-PCR anaysis. | |
| --- | --- |
| **miRNA** | **Primer sequence** |
| miR1039 | GTGCGAGACGGTCTCAAGGAT |
| miR1144 | TGGAACCGGGCAGCTCGGATG |
| miR1160 | CGAGAAGGAAGACAGACGGAT |
| miR1439 | TAATTGGGACGGAGTGAGTATT |
| miR156b | CACGACAGATAGAAAGCACAAT |
| miR168 | TCGCTTGGTGCAGGTCGGGA |
| miR1888 | TAAGTTAGATTTATGAAGGAT |
| miR2592 | ATGACTTGAGTGATGTGTGCTT |
| miR2607 | ATGTGATTATTGATTGTG |
| miR2927 | GTGTCACGTCGACGGAGCCCTG |
| miR3445 | TTTGGAGGTGAGTTGTTTGC |
| miR3629 | CCATTTTCTCGAGCCAAC |
| miR3706 | TATAGAGAAATGGTAAGA |
| miR3949 | TGATGATGAGGCAGAAAATGAG |
| miR415a | ACTGAGCAGCAACCAGAAC |
| miR4397 | TGCAAGATGTGGCGAATT |
| miR852 | GGAATCAAAGGCAGCTTATCAT |
| miR902a | AGACAGTCTGCATATAGC |
| miR7800 | TATTTTTGTGTCGTTATGGTC |
| miR7811 | TGAATGGAGATACGGAATGAAGC |
| 18S | GAGCTAATACGTGCAACAAACC |

| Table I. Forward primer sequences of 16 miRNA different expression patterns in FP and SP roots during developmental processes using qRT-PCR analysis. | |
| --- | --- |
| **miRNA-name** | **Primer sequence** |
| miR1147 | ATATCGGCCAAGTGGCAGA |
| miR160c | TGCCTGGCTCCCTGTATGCC |
| miR1851 | GGGTCTGGGATGGATTTGGC |
| miR1861b | CGAACTTGAACAAGAACTGCAG |
| miR2931 | ATTTATTGTTCGATGAAAA |
| miR3512 | GCAAATGATGACAAAATAG |
| miR3951 | AGAGACAGAGAGAGAAAA |
| miR7811 | GAATGGAGATACGGAATGAAGC |
| miR1115 | TGAGCTCGGCACTTTGGGAAGG |
| miR157a | TTGACAGAAGATAGCGAGCCC |
| miR165a | TCGGACCAGGCTTCATCCCCC |
| miR167d | GTTCTAGTACGACCGTCGAAT |
| miR168a | TCGCTTGGTGCAGGTCGGGAA |
| miR2663 | TTAAGAGGGCGTTTCAAATT |
| miR408a | ACAGAGACGAGACAGAGCATG |
| miR477c | AATCTCCCTCAAGGGCTTCTG |
| 18S | GAGCTAATACGTGCAACAAACC |
