# Supplementary material for: Transcriptome/Degradome-Wide Identification of R. glutinosa miRNAs and Their Targets: The Role of miRNA Activity in the Replanting Disease
Source: PLoS One. 2013 Jul 5;8(7):e68531. doi: 10.1371/journal.pone.0068531 (PMC3702588; doi:10.1371/journal.pone.0068531)

**File S2.** Additional figures


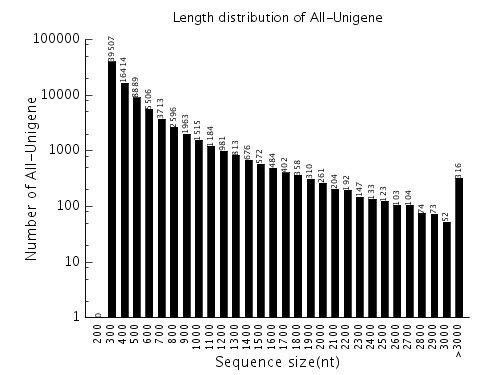
Figure A. Length distribution of All-unigenes in *R. glutinosa*.

Figure B. Venn diagrams for analysis of total (A) and unique (B) sRNAs between FP and SP *R. glutinosa*.


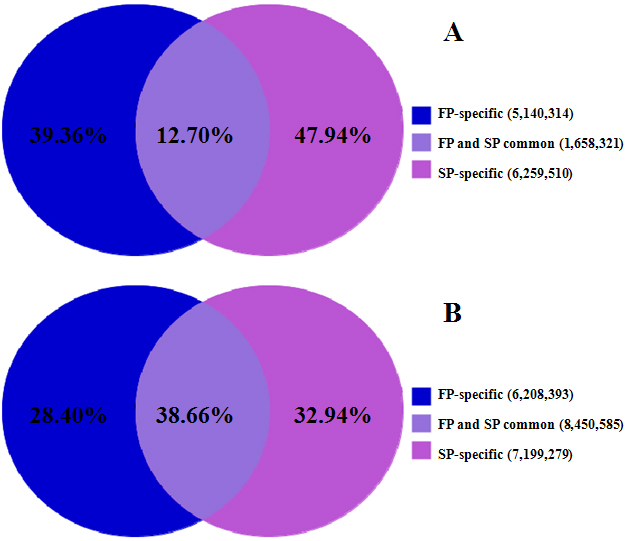


Figure C. Electrophoresis of identified 24 novel miRNA qRT-PCR products in FP or SP *R. glutinosa*. Note: 24 lanes represtent orderly as follows: lane 1, miR7797a; lane 2, miR7798; lane 3, miR7799; lane 4, miR7800; lane 5, miR7801; lane 6, miR7802; lane 7, miR7803a; lane 8, miR7804-5p; lane 9, miR7804-3p; lane 10, miR7805-5p; lane 11, miR7805-3p; lane 12, miR7806; lane 13, miR7807a-5p; lane 14, miR7807a-3p; lane 15, miR7803b-5p; lane 16, miR7803b-3p; lane 17, miR7808; lane 18, miR7809; lane 19, miR7810; lane 20, miR7972; lane 21, miR7811; lane 22, miR7807b-5p; lane 22, lane 23, miR7807b-3p; lane 23, miR7797b.

DL2000 1 2 3 4 5 6 7 8 9 10 11 12 13 14 15 16 17 18 19 20 21 22 23 24


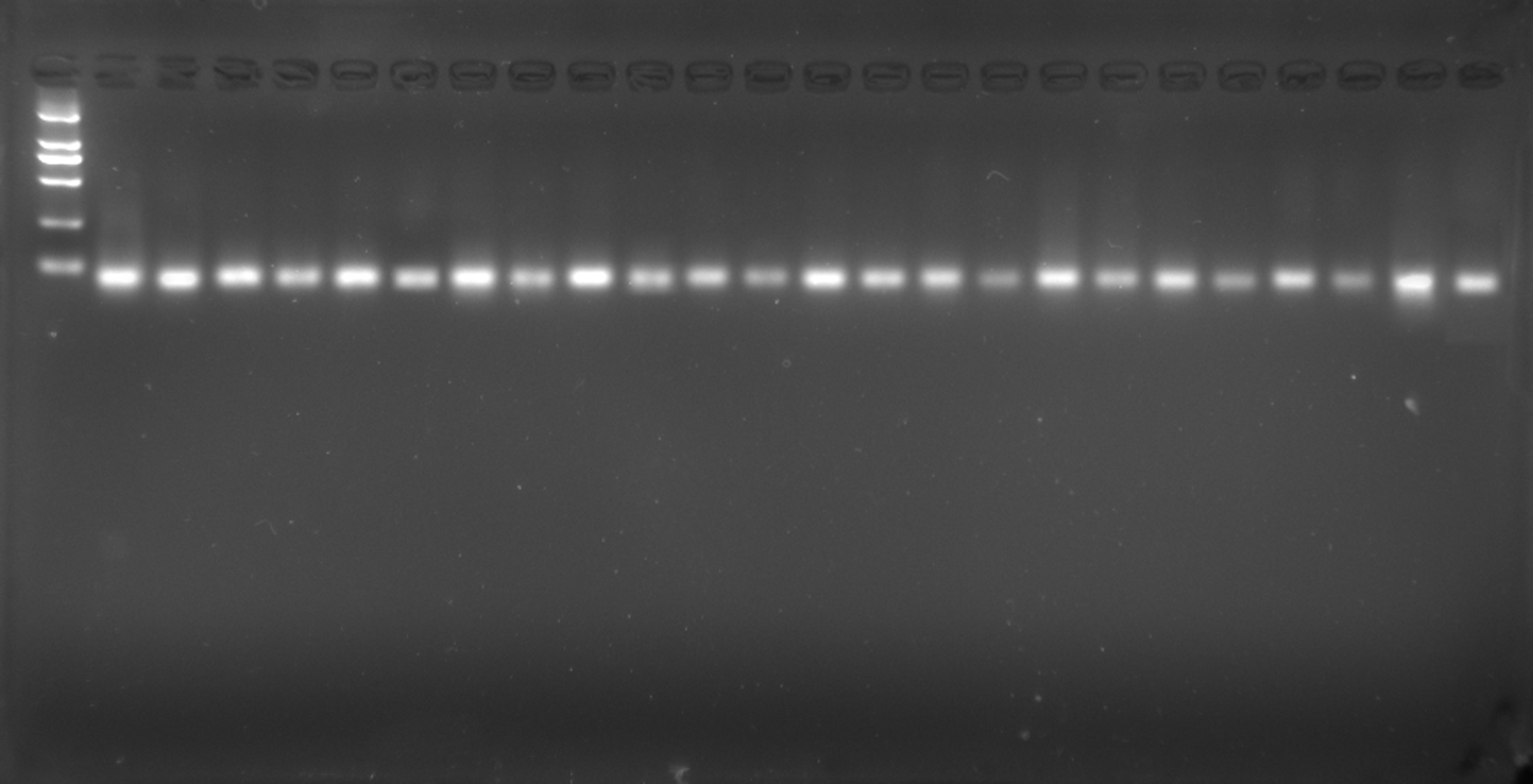

Supplement: File S2 — Additional figures. (DOC) [file pone.0068531.s002.doc]
