# Supplementary material for: Transcriptome/Degradome-Wide Identification of R. glutinosa miRNAs and Their Targets: The Role of miRNA Activity in the Replanting Disease
Source: PLoS One. 2013 Jul 5;8(7):e68531. doi: 10.1371/journal.pone.0068531 (PMC3702588; doi:10.1371/journal.pone.0068531)

### File S3. Secondary structure of novel miRNAs from *R. glutinosa*.

Secondary structure for “rgl-miR7797a”


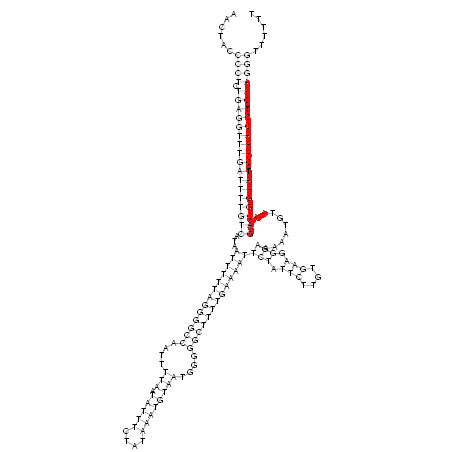


Secondary structure for “rgl-miR7797b”


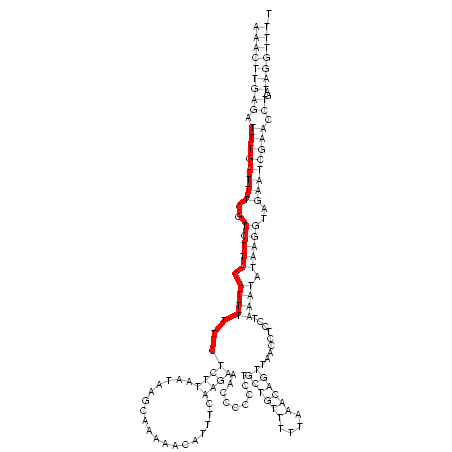


Secondary structure for ‘‘rgl-miR7798”


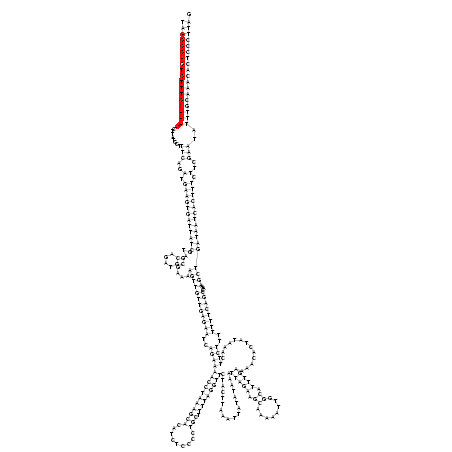


Secondary structure for “rgl-miR7799”


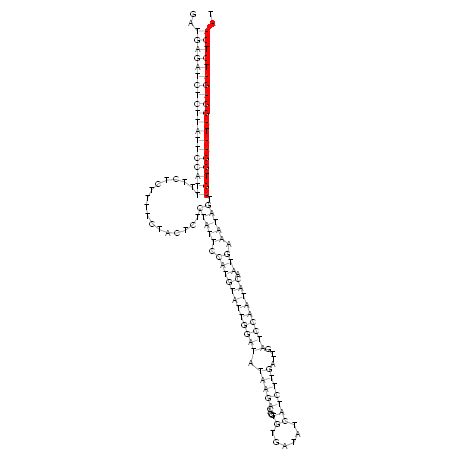


Secondary structure for “rgl-miR7800”


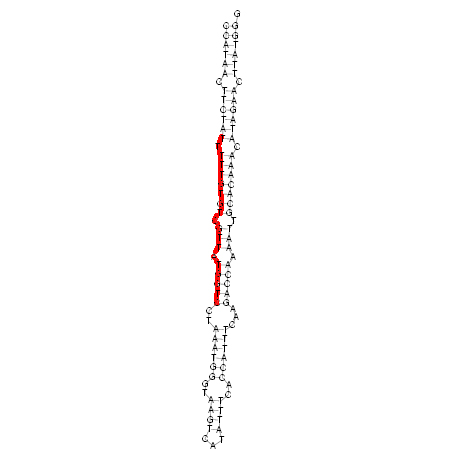


Secondary structure for “rgl-miR7801”


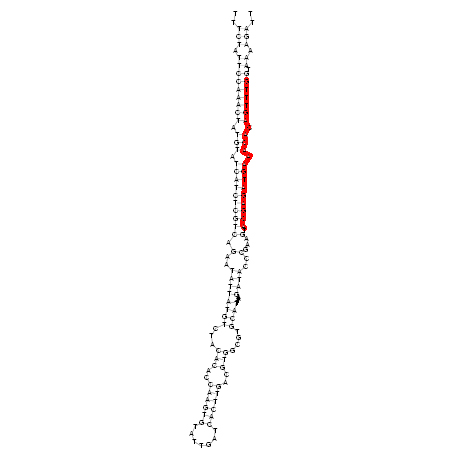


Secondary structure for “rgl-miR7802”


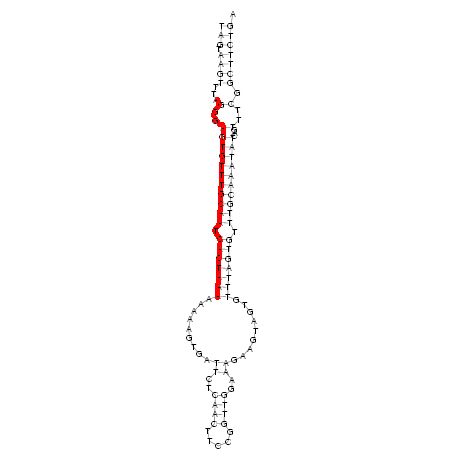


Secondary structure for “rgl-miR7803a”


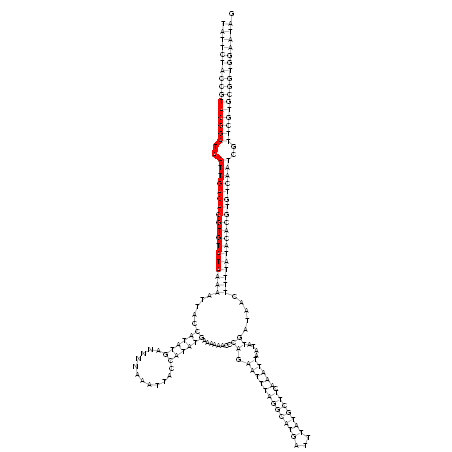


Secondary structure for “rgl-miR7803b-5p and rgl-miR7803b-3p”


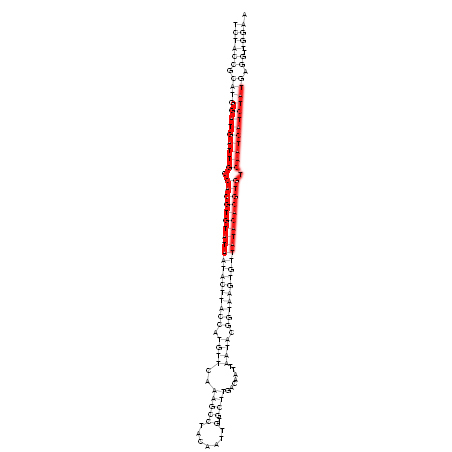


Secondary structure for “rgl-miR7804-5p and rgl-miR7804-3p”


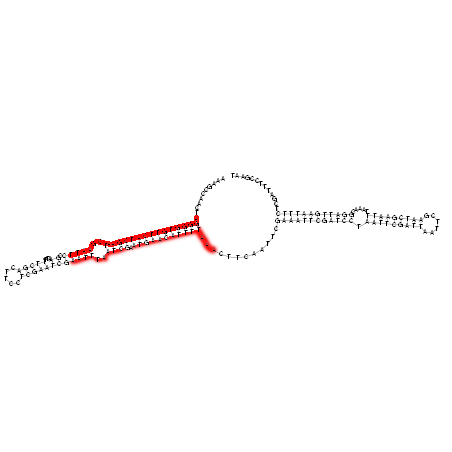


Secondary structure for “rgl-miR7805-5p and rgl-miR7805-3p”


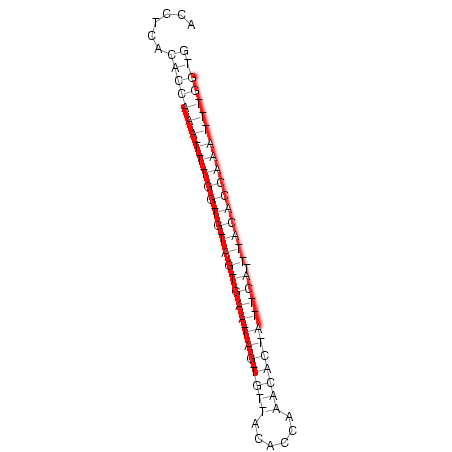


Secondary structure for “rgl-miR7806”


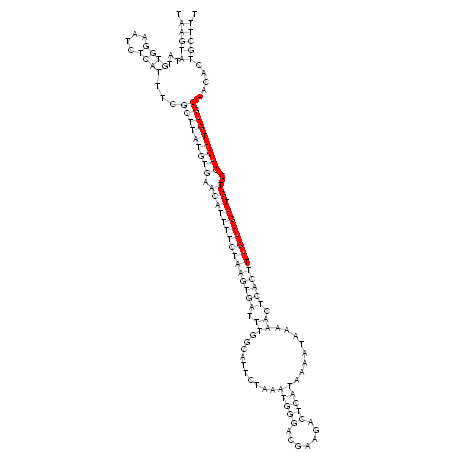


Secondary structure for “rgl-miR7807a-5p and rgl-miR7807a-3p”


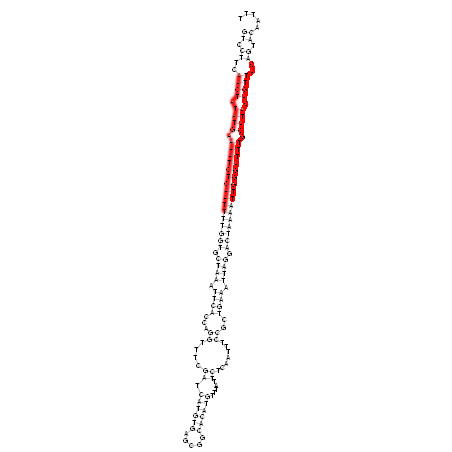


Secondary structure for “rgl-miR7807b-5p and rgl-miR7807b-3p”


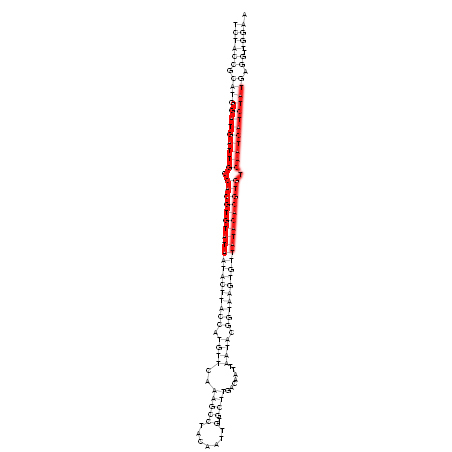


Secondary structure for “rgl-miR7808”


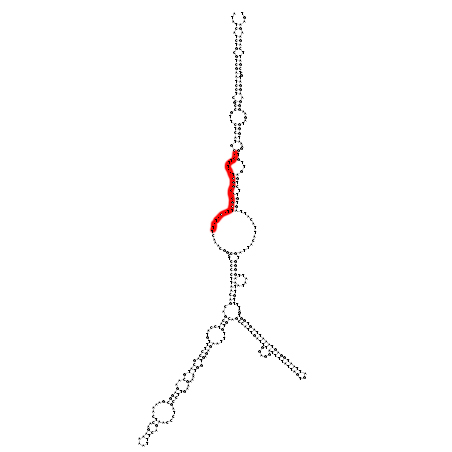


Secondary structure for “rgl-miR7809”


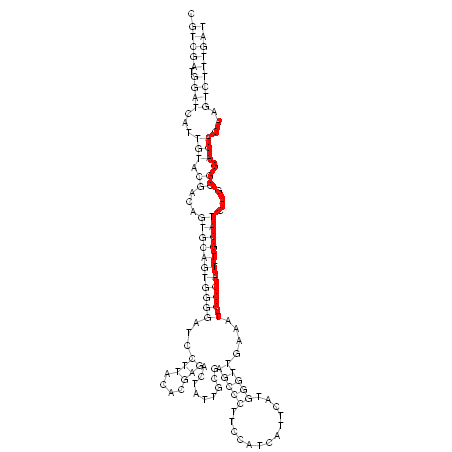


Sencondary sturcture for “rgl-miR7810”


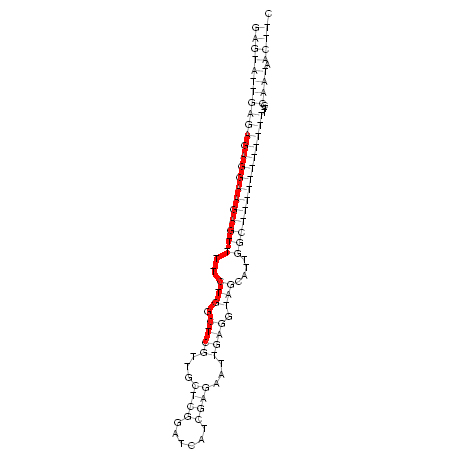


Secondary structure for “rgl-miR7811”


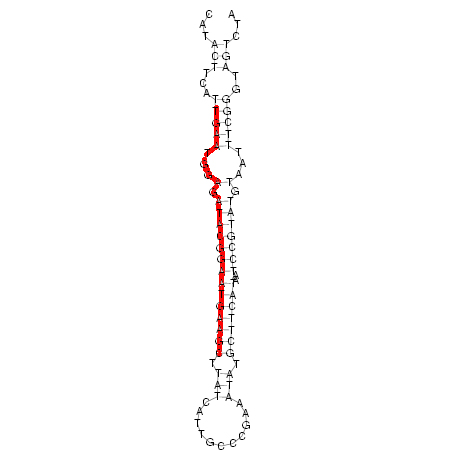


Secondary structure for “rgl-miR7972”


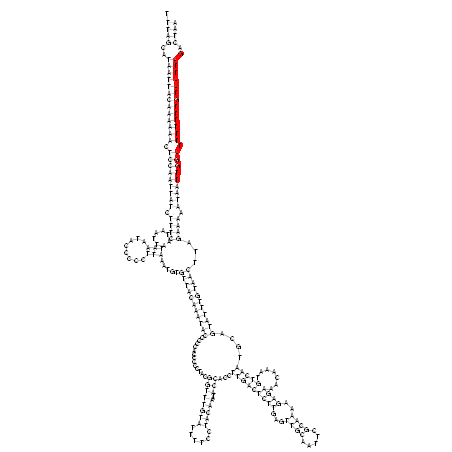

Supplement: File S3 — Secondary structure of novel miRNAs from R. glutinosa . (DOC) [file pone.0068531.s003.doc]
