# Supplementary material for: Transcriptome/Degradome-Wide Identification of R. glutinosa miRNAs and Their Targets: The Role of miRNA Activity in the Replanting Disease
Source: PLoS One. 2013 Jul 5;8(7):e68531. doi: 10.1371/journal.pone.0068531 (PMC3702588; doi:10.1371/journal.pone.0068531)
Supplement: File S4 — Target plots (t-plots) of selected 8 up- (A–H) and 8 down-regulated (I–P) miRNAs confirmed from the SP and FP degradome libraries, respectively. Note: T-plot (top) and miRNA: mRNA alignments (bottom). (DOC) [file pone.0068531.s004.doc]

**File S4.** Target plots (t-plots) of selected 8 up- (A-H) and 8 down-regulated (I-P) miRNAs confirmed from the SP and FP degradome libraries, respectively. Note: T-plot (top) and miRNA: mRNA alignments (bottom).

(A) miR1147 target (Unigene68408_All)

**Transducin family protein**

5' AUAUCGGCCAAGUGGCAGA 3' **miR1147**

||||||o||||| ||| |

3' GUUCUAUAGCUGGUUCUCCGAGUUAUAGUGGA 5' **Unigene68408_All**

(B) miR160 target (Unigene38897_All)

**Auxin response factor 10**

5’ UGCCUGGCUCCCUGUAUGCCA 3’ **miR160c**

||||||||||||||||oo||

3’ UCGUACGGACCGAGGGACAUGUGGACGUCUUU 5’ **Unigene38897_All**

(C) miR1851 target (Unigene27779_All)

**ALY protein**

5' GGGUCUG-GGAUGGAUUUGGC 3' **miR1851**

|| |||| |||||||o|||||

3' GAACCAAGACGCCUACCUGAACCGUUAAGUUU 5' **Unigene27779_All**

(D) miR1861 target (Unigene26519_All)

**Potassium ion transmembrane transporUer**

5' CGAACUUGAACAAGAACUGCAG 3' **miR1861b**

| ||||o||||||||| |o|||

3' GAUUG-UUGAGCUUGUUCUUAAUGUCCCAGUU 5' **Unigene26519_All**

(E) miR2931 target (Unigene44304_All)

**Histidine kinase 3B**

| 5' AUUUAUUGUUCGAUGAAAA 3'  **miR2931**  |||| || |||||||||||  3' GGUCUAAAGAAGAAGCUACUUUUGGUACACAU 5'  **Unigene44304_All** |
| --- |

(F) miR3512 target (Unigene35293_All)

**ABC transporter family**

5' GCAAAUGAUGACAAAAUAGA 3'  **miR3512**

|||||o| |||||||| |||

3' GUCACGUUUGCAACUGUUUUCUCUUGUGGUAC 5' **Unigene35293**

(G) miR3951 target (Unigene11404_All)

**MYB transcription factor MYB127**

5' AGAGACAGAGAGAGAAAAA 3' **miR3951**

||||| ||||||||| | |

3' UCUCUCUCUCUCUCUCUCUCUCUCUCUCUCGA 5' **Unigene11404**

(H) miR7811 target (Unigene74608_All)

**Structural maintenance of chromosomes family protein**

5' UGAAUGGAGAUACGGAAUGAAGC 3' **miR7811**

o|||||||||||o|| |||||

3' GAAGCUUUACCUCUAUGUCU-ACUUCUUCCAU 5' **Unigene74608_All**

(I) miR1115 target (Unigene5576_All)

**Probable thiol methyltransferase 2**

5' UGAGCUCGGCACUUUGGGAAGG 3' **miR1115**

|||||||o||||||||||||||

3' UUGUACUCGAGACGUGAAACCCUUCCCUAGG 5' **Unigene5576_All**

(J) miR156/157 target (Unigene35337_All)

**Squamosa promoter-binding protein**

5' UUGACAGAAGAUAGCGAGCCC 3' **miR157a** |||||||||||||o|||| |

3' CGGCUACUGUCUUCUAUCUCUCGUGCUAGUCU 5' **Unigene35337_All**

(K) miR165 target (Unigene19246_All)

**Class III HD-Zip protein 8**

5’ UCGGACCAGGCUUCAUCCCCC 3’ **miR165a**

o||||||||||||||||||

3’ CUUAGGCCUGGUCCGAAGUAGGGUCCGUAAAC 5’ **Unigene19246_All**

**(**L) miR167 target (Unigene6351_All)


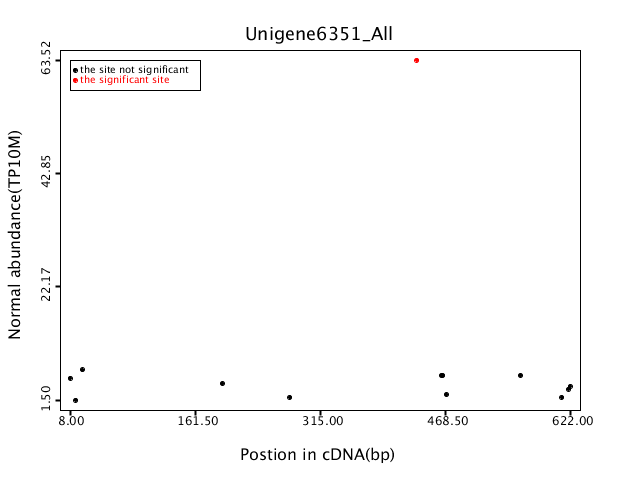


**Auxin response factor 8**

5' GUUCUAGUACGACCGUCGAAU 3' **miR167d**

o||||||||||||| |||||

3' TCAAAGAUCAUGCUGGGAGCUUGUAUUCGUC 5' **Unigene6351_All**

(M) miR168 target (Unigene45023_All)

**AGO1-1**

5' UCGCUUGGUGCAGGUCGGGAA 3' **miR168a**

| ||||||||||| ||||||
3' AGCCAUCGAACCACGUCGAGCCCUCGACCAC 5' **Unigene45023_All**

(N) miR2663 target (Unigene7358_All)

**Endonuclease**

5' UUAAGAGGGCGUUUCAAAUU 3' **miR2663**

oo||| ||||||||| ||||

3' AGUGGUUCCCCCGCAAAGAUUAACGGGGUGAC 5' **Unigene7358_All**

(O) miR408 target (Unigene40796_All)

**Lateral organ boundaries domain protein**

5' ACAGAGAC-GAGACAGAGCAUG 3' **miR408a**

|||||||| |||| |||||| |

3' UGUUGUCUCUGACUCUUUCUCGUCCAGAAACU 5' **Unigene40796_All**

(P) miR477 target (Unigene63464_All)

**RNA helicase**

5' AAUCUCCCUCAAGGGCUUCUG 3'  **miR477c**

o||||||||||| |||||| |

3' AAUUGUAGAGGGAGUUGCCGAAG-CUUUACGC 5' **Unigene63464_All**
